# Establishing a reference array for the CS-αβ superfamily of defensive peptides

**DOI:** 10.1186/s13104-016-2291-0

**Published:** 2016-11-18

**Authors:** D. Ellen K. Tarr

**Affiliations:** Department of Microbiology and Immunology, Arizona College of Osteopathic Medicine, Midwestern University, Glendale, AZ USA

**Keywords:** Antimicrobial peptide, CS-αβ superfamily, Fungal defensin, Invertebrate defensin, Invertebrate immunity, Plant defensin, Scorpion toxin

## Abstract

**Background:**

“Invertebrate defensins” belong to the cysteine-stabilized alpha-beta (CS-αβ), also known as the scorpion toxin-like, superfamily. Some other peptides belonging to this superfamily of defensive peptides are indistinguishable from “defensins,” but have been assigned other names, making it unclear what, if any, criteria must be met to qualify as an “invertebrate defensin.” In addition, there are other groups of defensins in invertebrates and vertebrates that are considered to be evolutionarily unrelated to those in the CS-αβ superfamily. This complicates analyses and discussions of this peptide group. This paper investigates the criteria for classifying a peptide as an invertebrate defensin, suggests a reference cysteine array that may be helpful in discussing peptides in this superfamily, and proposes that the superfamily (rather than the name “defensin”) is the appropriate context for studying the evolution of invertebrate defensins with the CS-αβ fold.

**Methods:**

CS-αβ superfamily sequences were identified from previous literature and BLAST searches of public databases. Sequences were retrieved from databases, and the relevant motifs were identified and used to create a conceptual alignment to a ten-cysteine reference array. Amino acid sequences were aligned in MEGA6 with manual adjustments to ensure accurate alignment of cysteines. Phylogenetic analyses were performed in MEGA6 (maximum likelihood) and MrBayes (Bayesian).

**Results:**

Across invertebrate taxa, the term “defensin” is not consistently applied based on number of cysteines, cysteine spacing pattern, spectrum of antimicrobial activity, or phylogenetic relationship. The analyses failed to reveal any criteria that unify “invertebrate defensins” and differentiate them from other defensive peptides in the CS-αβ superfamily. Sequences from various groups within the CS-αβ superfamily of defensive peptides can be described by a ten-cysteine reference array that aligns their defining structural motifs.

**Conclusions:**

The proposed ten-cysteine reference array can be used in addition to current nomenclature to compare sequences in the CS-αβ superfamily and clarify their features relative to one another. This will facilitate analysis and discussion of “invertebrate defensins” in an appropriate evolutionary context, rather than relying on nomenclature.

**Electronic supplementary material:**

The online version of this article (doi:10.1186/s13104-016-2291-0) contains supplementary material, which is available to authorized users.

## Background

Defensin nomenclature has a complex history (Table 1). “Defensins” originally referred to a set of three human neutrophil peptides that show activity against *Staphylococcus aureus, Pseudomonas aeruginosa, Escherichia coli, Cryptococcus neoformans,* and herpes simplex virus, type 1 [1]. The general term “defensin” seemed appropriate due to the broad spectrum of activity. These peptides are 29–30 amino acids long, contain six cysteines that form three disulfide bonds, and are homologous to a group of six peptides from rabbit neutrophils [2, 3].

**Table 1:** Landmark papers in identification and establishment of the CS-αβ superfamily

Year identified	Peptide name, source, and significance	#C	Antimicrobial activity	References
1985	Charybdotoxin from *Leiurus quinquestriatus* (deathstalker, Palestine/Israeli yellow scorpion), inhibits Ca^2+^-activated K^+^ channels	6	G+, G−, Y	[13, 17, 18]
1985	Defensins from human neutrophils, similar to peptides isolated from rabbit neutrophils	6	G+, G−, Y, V	[1]
1988	Sapecins from *Sarcophaga peregrina* (flesh fly), similarity to mammalian defensins noted, but the name “defensin” was not applied to these peptides	6	*G+*, G−	[5, 72, 99]
1989	Phormicins/*Phormia* defensins from *Protophormia terraenovae* (northern blow fly, blue-bottle fly), proposal of term “insect defensin”	6	*G+*, G−, F (Y)	[4, 46, 47]
1991	Establishment of CSH motif in arthropod neurotoxic peptides	4		[12]
1992	RsAFP1/RsAFP2–antifungal peptides from *Raphanus sativus* (radish), noted that based on structure, RsAFPs belonged to a superfamily of small, basic, cysteine-rich proteins with antibacterial activity (including plant thionins, and mammalian and insect defensins), but that RsAFPs were unique due to their specific activity against filamentous fungi; “plant defensin” term proposed in 1995	8	RsAFP1: *F* (G+, G−, Y, C, H) RsAFP2: *F*, G+ (G−, Y, C, H)	[8, 9, 61]
1993	Scorpion defensin from *Leiurus quinquestriatus* (deathstalker, Palestine/Israeli yellow scorpion), similarity to both insect defensins and scorpion toxins noted as well as the ability of the scorpion to produce both a toxin and a defensin	6	G+ (G−)	[28]
1994	Defensin from *Drosophila melanogaster* (fruit fly)	6	G+	[50]
1994	Drosomycin from *Drosophila melanogaster* (fruit fly), noted similarity to plant antifungal peptides	8	*F*, Y, P (G+, G−, H)	[10, 100]
1995	Establishment of CS-αβ fold by adding third disulphide bond to the CSH motif (study used *Phormia* defensin A)			[11]
1996	MGD-1–defensin 1 from *Mytilus galloprovincialis* (Mediterranean mussel), considered to be part of arthropod defensin group with two additional cysteines	8	G+, G−, F (C), some fragments active against Y and P	[34, 54, 55, 101, 102]
1996	Defensins and mytilins from *Mytilus edulis* (blue mussel), some sequences incomplete, mytilins proposed as a different group based on position of cysteines in primary structure	6–8	*G+*, G−	[57]
1996	ASABF–antibacterial factor from *Ascaris suum* (large roundworm of pigs), noted similarity to plant defensins and drosomycin	8	G+, G− (F)	[59]
1999	Myticins from *Mytilus galloprovincialis* (Mediterranean mussel), myticins proposed as a different group based on position of cysteines in primary structure	8	G+, G−, F (P)	[56]
2002	Ce-ABF2–antibacterial factor 2 from *Caenorhabditis elegans*	8	G+, G−, Y	[60]
2004	Theromacin from *Theromyzon tessulatum* (duck leech), cysteine array originally thought to not be similar to arrays of other C-rich peptides	10	G+ (G−, F)	[39]
2005	Plectasin–fungal defensin from *Pseudoplectania nigrella* (ebony cup)	6	G+ (G−)	[33]
2007	AdDLP–defensin-like peptide from *Anaeromyxobacter dehalogenans* (bacteria) hypothesized ancestor of group, has only the CSH motif	4	P (G+, G−, F, Y, H)	[19, 20]
2009	Hydramacin from *Hydra magnipapillata,* noted similarity to scorpion toxin superfamily	8	G+, G−	[40, 41]
2011	ASABF-related peptide from *Suberites domuncula* (sponge)	8	G+, G−, F, Y, H	[36]
2012	Neuromacin and theromacin from *Hirudo medicinalis* (medicinal leech)	8–10	G+, G−	[40]
2012	Micasin–defensin-like peptide from *Arthroderma otae/Microsporum canis*	6	G+, G− (F, Y, H)	[24]
2013	Mytimacin -AF from *Achatina fulica *(giant African snail)	10	G+, G−, Y (H)	[44]
2014	Cremycins–drosomycin-like antifungal peptides from *Caenorhabditis remanei,* cysteine number and spacing not consistent with drosomycin, not all have antifungal activity	6	Cremycin 5: F, Y (G+, G−, H) Cremycin-15: G+, G− (F, Y)	[21]

Peptides are listed in order of initial identification and description. The activity column lists activity against Gram-positive bacteria (G+), Gram-negative bacteria (G−), filamentous fungi (F), yeast (Y), viruses (V), and protozoa (P), as well as cytotoxic (C) and hemolytic (H) activity. The peptide has the activity shown if the abbreviation is shown without parentheses, and has been tested but not shown to have the activity if shown in parentheses. If a dominant activity has been determined, the abbreviation is shown in italics; any activity not shown has not been tested for that peptide. Additional references that establish activity or structure are included

The term “insect defensin” was proposed by Lambert et al. in their description of two small cysteine-rich peptides from *Phormia terranovae* (phormicins) [4]. These peptides, along with sapecins identified a year earlier, showed activity primarily against Gram-positive bacteria and appeared to have some sequence homology to the mammalian defensins [4, 5]. It is now clear that observed similarities between insect and mammalian defensins are most likely due to convergence, but the name “defensin” has been retained [6, 7]. Two antifungal peptides with similarity to defensins were isolated from radish [8], and the term “plant defensin” was proposed after cloning of the full-length sequences for these peptides, which have eight cysteines instead of six [9]. While invertebrate peptides are the focus of this study, plant defensins are part of the same superfamily and the similarity of drosomycin from *Drosophila* to plant peptides has been acknowledged since it was first described [10].

The structure that unifies the invertebrate and plant defensins is the cysteine-stabilized alpha-beta (CS-αβ) motif established by Cornet et al. for *Phormia* defensin A (phormicin A), which has an alpha helix followed by two antiparallel beta sheets, and is stabilized by three disulfide bonds [11]. Two of the three bonds correspond to a smaller structural motif that had been previously described in toxic peptides from arthropods, the cysteine-stabilized α-helix (CSH) [12]. Sequences with this fold also tend to have the γ-core motif, an enantiomeric motif of 8–16 amino acids generally containing a conserved GXC or CXG and forming a β-hairpin structure [13]. This motif is found not only in sequences with the CSH and CS-αβ motifs, but in nearly all groups of cysteine-containing defense peptides [13, 14].

Invertebrate defensins and other peptides containing the CS-αβ fold form the CS-αβ superfamily of proteins, also known as the scorpion toxin-like superfamily in the SCOP [15] and new SCOP2 [16] databases. This superfamily includes five families of defensive peptides: long-chain scorpion toxins, short-chain scorpion toxins, defensin MGD-1, insect defensins, and plant defensins [15, 16]. Charybdotoxin from the deathstalker scorpion was identified and described around the same time as mammalian and insect defensins [17, 18], but its antimicrobial activity wasn’t tested until much later [13]. The superfamily may have originated from myxobacterial sequences that contain the CSH motif [19]. Although the GXC/CXG of the γ-core motif is missing, *Anaeromyxobacter dehalogenans* defensin-like peptide (AdDLP) has a defensin-like structure and activity against *Plasmodium berghei,* in spite of showing no other antimicrobial or hemolytic activity thus far [20].

A protein’s nomenclature generally reflects its characteristics and how it is related to other proteins. Ideally, proteins named as part of a group share important characteristics and/or a common evolutionary history not shared with other proteins. As additional members of the CS-αβ superfamily have been identified from fungi as well as mollusks, nematodes, annelids, and other invertebrate taxa, the nomenclature and associated criteria have become confusing at best. A peptide named as a “defensin” may have six or eight cysteines with varying antimicrobial activities. Depending on the taxonomic group, a peptide with the characteristics of “invertebrate defensins” may have 4–12 cysteines and be called a mycin, macin, mytilin, myticin, antibacterial factor, defensin-like peptide/protein, or drosomycin-like antifungal peptide (Table 1). The clearest demonstration of the inconsistent and confusing nomenclature is the cremycins from *Caenorhabditis remanei.* These peptides are described as drosomycin-like antifungal peptides, but their sequences are not particularly drosomycin-like and only one of the two tested (of 15 total) has antifungal activity [21]. To further confuse the nomenclature, invertebrate big defensins are not part of the CS-αβ superfamily, but are more likely related to vertebrate defensins [22]. This paper investigates the criteria for classifying a peptide as an invertebrate defensin, suggests a reference cysteine array that may be helpful in discussing peptides in the CS-αβ superfamily, and proposes that the superfamily is the appropriate context for studying the evolution of invertebrate defensins with the CS-αβ fold.

## Results and discussion

### CS-αβ reference array

**Fig. 1:**
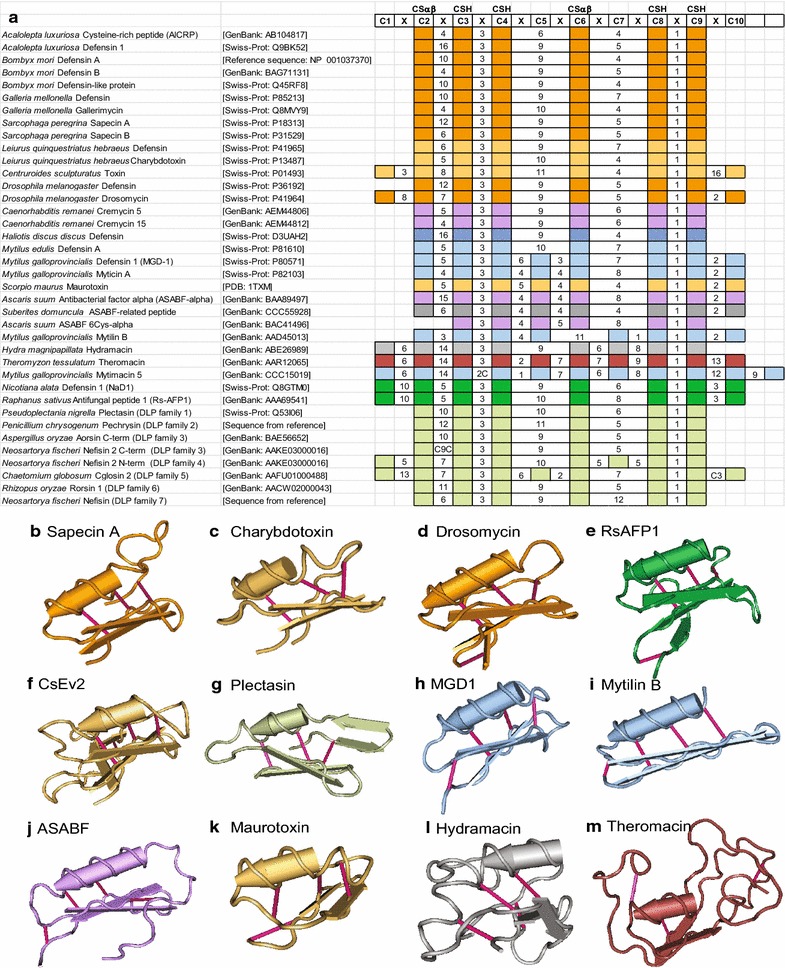
Names, cysteine patterns, and structures of representative CS-αβ peptides. **a** Names of representative sequences with accession numbers and alignment of mature peptide to reference array. Cysteines 3, 4, 8, and 9 form the cysteine-stabilized helix (CSH) motif, and cysteines 2 and 6 form a third bond to complete the CS-αβ fold. Alignment of all retrieved sequences to the reference array can be found in Additional file 1: Figure S1. **b**–**m** Structures of representative peptides with disulfide bonds shown in bright pink: **b** *Sarcophaga peregrina* Sapecin A [PDB: 1L4V], **c** *Leiurus quinquestriatus hebraeus* Charybdotoxin [PDB: 2CRD], **d** *Drosophila melanogaster* Drosomycin [PDB: 1MYN], **e** *Raphanus sativus* RsAFP1 [PDB: 1AYJ], **f** *Centruroides sculpturatus* CsEv2 [PDB: 1JZB], **g** *Pseudoplectania nigrella* Plectasin [PDB: 1ZFU], **h** *Mytilus galloprovincialis* MGD1 [PDB: 1FJN], **i** *Mytilus edulis* Mytilin B [PDB: 2EEM], **j** *Ascaris suum* ASABF [PDB: 2D56], **k** *Scorpio maurus* Maurotoxin [PDB: 1TXM], **l** *Hydra magnipapillata* Hydramacin [PDB: 2K35], and **m** *Hirudo medicinalis* Theromacin [PDB: 2LN8]. Major taxonomic groups are color-coded: Annelida (*dark rose*), Arachnida (*light orange*), Bivalvia (*light blue*), Cnidaria (*light grey*), Fungi (*light green*), Hexapoda (*orange*), Nematoda (*lavender*), Plantae (*green*), and Porifera (*dark grey*)

It is often the case that the first, and possibly only, information available for a CS-αβ peptide is its sequence, with activity and structure studied later or not at all. While sequence comparison may seem straightforward, different members of this superfamily have different numbers and bonding patterns of cysteines. For example, insect defensins are described as having the pattern C1–C4, C2–C5, C3–C6; nematode ABFs have C1–C5, C2–C6, C3–C7, C4–C8. From these descriptions, it isn’t clear that the first three disulfide bonds of nematode ABFs are structurally the same as the three found in insect defensins (i.e., C4 of insect defensins aligns with C5 of nematode ABFs). Most CS-αβ peptides have 6–10 cysteines, so I aligned sequences to a ten-cysteine array. C3, C4, C8, and C9 correspond to the CSH motif [12]; the addition of C2 and C6 completes the CS-αβ fold [11]. The C of the GXC in the γ-core motif is generally C6. CS-αβ sequences were aligned to this array using these cysteines as guides to facilitate comparison of cysteine spacing patterns (Fig. 1a; Additional file 1: Figure S1). Additional cysteines at the N or C-terminus of the conserved array are represented by additional filled boxes. In the case there are additional cysteines within the conserved array, they are represented as “C.” For example, two filled boxes with “2C” in between would be interpreted as “CXXCC,” with “C2C” in between as “CCXXCC,” and with “2C1” in between as “CXXCXC.” It is unlikely that established names for peptides will be changed for consistency, and revising names will make reading previous literature confusing. A reference array for comparing these sequences that can be used in addition to current nomenclature is a reasonable solution.

### Nomenclature is not consistent with cysteine pattern

Figure 1a shows the names and cysteine patterns of selected members of the CS-αβ superfamily aligned to the proposed reference array. The representative sequences were chosen to highlight the inconsistency in naming of these peptides, and a more complete alignment can be found in Additional file 1: Figure S1. The structures for several of these have been reported and are shown in Fig. 1b–m.

Sapecin A and other typical insect defensins have six cysteines corresponding to C2–C4, C6, C8, and C9 of the reference array (Fig. 1a, b). The n-loop is variable, with 4-16 amino acids separating C2 from C3. Some previous work proposed three categories of insect defensins: (1) “classical insect-type defensins” (CITDs) with longer n-loops restricted primarily to phylogenetically recent insect orders, (2) “ancient invertebrate-type defensins” (AITDs) with shorter n-loops found in primitive insect taxa as well as other invertebrates, and (3) “plant/insect-type defensins” (PITDs) that have a fourth disulfide bond found in plants and *Drosophila* [6, 23, 24]. Given that a single insect species may have both CITDs and AITDs, this classification is confusing and of limited utility. Examples show that “defensin” is not consistently applied to either long or short n-loop insect sequences (Fig. 1a: *Acalolepta luxuriosa, Bombyx mori, Galleria mellonella,* and *Sarcophaga peregrina*). A recent review [25] combined CITDs and AITDs into “arthropod and mollusk-type six-cysteine defensins,” but a combination of literature and database searches shows sequences from nematodes, tardigrades, velvet worms, crustaceans, and fungi with cysteine arrays consistent with this spacing (Additional file 1: Figure S1). Charybdotoxin and other short-chain scorpion toxins in the CS-αβ superfamily also have this cysteine pattern, and the structure of charybdotoxin is similar to that of sapecin (Fig. 1a–c) [26, 27]. The scorpion *Leiurus quinquestriatus* produces both charbydotoxin and a defensin with a very similar cysteine pattern (Fig. 1a) [17, 18, 28]. Therefore, it is not possible to determine whether a six-cysteine CS-αβ sequence with the typical insect spacing is a toxin or an antimicrobial peptide, let alone whether it is called a defensin, defensin-like peptide/protein, cysteine-rich peptide/protein, or a name derived from the species (gallerimycin, sapecin, etc.).

The additional cysteines in drosomycin (Fig. 1d) [29] and most plant defensins (represented by RsAFP1, Fig. 1e) [30] correspond to C1 and C10 of the reference array. Other than the drosomycin family in *Drosophila* and plant defensins, only one nematode sequence seems consistent with this spacing (NEMBASE: PSC02929). Zhu and Gao reported a family of drosomycin-type antifungal peptides (DTAFPs) from *Caenorhabditis remanei* called “cremycins” [21]. However, all 15 cremycins have only six cysteines (instead of the eight found in drosomycin), and their spacing is consistent with insect defensins (Fig. 1a; Additional file 1: Figure S1) [21]. Long-chain scorpion toxins, such as from *Centruroides sculpturatus,* also have additional cysteines corresponding to C1 and C10 that form a fourth disulfide bond, but the sequence spacing is characterized by a long C-terminal extension between C9 and C10 that is not present in drosomycin and plant defensins (Fig. 1a, d–f) [31, 32]. Two *Hypsibius* (tardigrade) and four *Schistosoma* (trematode) sequences fit this pattern (Additional file 1: Figure S1), suggesting they might have toxic activity instead of or in addition to antimicrobial activity.

In contrast to the relative homogeneity of plant defensins, seven families of fungal defensins/defensin-like peptides (DLPs) have been identified [23, 24]. The cysteine number and spacing of families 1, 2, 6, 7, and some of 3 is consistent with the insect spacing, while the patterns for most members of 3, and families 4 and 5 are found almost exclusively in fungi (Fig. 1a; Additional file 1: Figure S1). Plectasin (in fDLP family 1) has an n-loop similar in length to sapecin A, but may form additional β-sheets (Fig. 1b, g) [33].

Mollusks and nematodes both express CS-αβ sequences with eight cysteines corresponding to C2–C6, C8, C9, and C10. In mollusks, most work has focused on mussels and oysters, leading to three groups that fit this pattern (defensins, myticins, and mytilins; Fig. 1a; Additional file 1: Figure S1). The nearly identical spacing for mollusk defensins and myticins makes this an ineffective means of differentiation; however, mytilin B has longer β-sheets than MGD-1 (Fig. 1h, i) [34, 35] and the GXC motif aligns with C7 of the reference array instead of C6. Nematode sequences with a similar cysteine pattern and structure to mollusk defensins with eight cysteines have been traditionally called “antibacterial factors” (ABFs) instead of “nematode defensins” (Fig. 1a, h, j; Additional file 1: Figure S1). Nematode CS-αβ peptides tend to have a longer n-loop, but this is not always the case (Fig. 1a; Additional file 1: Figure S1). A sequence from the sponge *Suberites domuncula* is referred to as an ASABF-type antimicrobial peptide [36], but is arguably just as similar to mollusk defensins and myticins (Fig. 1a). Some eight-cysteine potassium-channel toxins from scorpions are also consistent with the mollusk/nematode cysteine pattern and structure (represented by Maurotoxin, Fig. 1a, k) [37]. Since there doesn’t seem to be a consensus that “defensin” should apply only to six-cysteine sequences, there seems to be no reason that nematode “antibacterial factors” could not be referred to as “nematode defensins.”

In contrast to the majority of nematode sequences, ASABF 6-Cys-alpha has only six cysteines; however, the cysteines correspond to C3–C6, C8, and C9 of the reference array instead of the six found in typical insect defensins. The missing cysteines do not correspond to a disulfide bond-forming pair, so the authors suggest the bonding pattern may be different compared to most invertebrate defensins [38]. The structure will have to be experimentally determined to address this possibility.

The macins are a family of peptides that have not usually been included in analyses of defensins and defensin-like peptides, but clearly have the CS-αβ fold. Macins were originally described from annelids [39, 40] and have been reported from the cnidarian *Hydra magnipapillata* [40, 41], the mussels *Hyriopsis cumingii* [42] and *Mytilus galloprovincialis* [43], and the giant African land snail, *Achatina fulica* [44]. The addition of a fourth bond formed by C1 and C7 as seen in hydramacin (Fig. 1a, l) [41] may be a defining characteristic of macins. In ten-cysteine macins such as theromacin, the fifth bond is formed by C5 and C10 (Fig. 1a, m) [40]. Diverse invertebrate taxa have sequences with 8–12 cysteines consistent with the macin pattern (Additional file 1: Figure S1) [43]. Due to uncertainty regarding the presence of pro-peptides, some of these may have nine cysteines (Additional file 1: Figure S1). These peptides may act as dimers, as has been suggested for the scorpion lipolysis activating peptide LVP1 (a peptide similar to scorpion sodium-channel toxins; Additional file 1: Figure S1) [45].

### Nomenclature is not consistent with specific antimicrobial activity

It is reasonable to suggest that invertebrate defensins and related peptides be named based on their spectrum of antimicrobial activity rather than by features of their primary sequence. A barrier to classification and naming of CS-αβ sequences by function is that not all peptides are tested for activity prior to reporting. Of those that are, there is a great deal of variability in the extent of antimicrobial activity testing. Some peptides are tested against a wide variety of organisms, but others are only tested against a representative species in the pathogen group the peptide is suspected to be active against. Representative peptides used to illustrate the lack of nomenclature consistency are shown in Table 2; Additional file 2: Table S1 summarizes available antimicrobial activity for the CS-αβ peptides considered in this study.

**Table 2:** Antimicrobial activity of representative CS-αβ peptides

Species	Name [accession number]	#C	γ-core	Activity	References
*Sarcophaga peregrina*	Sapecin, Sapecin A [Swiss-Prot: P18313]	6	Yes	*G+*, G−	[5, 72, 73, 99]
	Sapecin B [Swiss-Prot: P31529]	6	No	*G+* (G−, Y)	
*Protophormia terraenovae*	Phormicin, defensin A [Swiss-Prot: P10891]	6	Yes	*G+*, G−, F (Y)	[4, 46, 47]
*Apis mellifera*	Royalisin [Swiss-Prot: P17722]	6	Yes	*G+*, G−	[49, 103]
*Drosophila melanogaster*	Defensin [Swiss-Prot: P36192]	6	Yes	G+	[50]
	Drosomycin [Swiss-Prot: P41964]	8	Yes	*F*, Y, P (G+, G−, H)	[10, 100]
*Heliothis virescens*	Heliomicin [GenBank: ACR78445]	6	Yes	*F*, Y (G+, G−)	[46]
*Galleria mellonella*	Gallerimycin [Swiss-Prot: Q8MVY9]	6	Yes	F (G+, G−, Y)	[52]
	Defensin [Swiss-Prot: P85213]	6	Yes	F, Y (G+, G−)	[51]
*Ixodes scapularis*	Scapularisin (Scapularisin 6) [GenBank: AAV74387]	6	Yes	G+, F (G−)	[53, 104]
*Ixodes scapularis*	Scapularisin 3 [GenBank: EEC13914]	6	Reverse	F (G+, G−)	[53, 70]
*Leiurus quinquestriatus*	Charybdotoxin [Swiss-Prot: P13487]	6	Yes	G+, G−, Y	[13, 17, 18]
*Leiurus quinquestriatus*	Defensin [Swiss-Prot: P41965]	6	Yes	G+ (G−)	[28]
*Mytilus galloprovincialis*	MGD-1 [Swiss-Prot: P80571]	8	Yes	G+, G−, F (C)	[34, 54, 55, 101]
*Mytilus galloprovincialis*	Myticin A [Swiss-Prot: P82103]	8	No	G+ (G−, F, P)	[56]
	Myticin B [Swiss-Prot: P82102]	8	No	G+, G−, F (P)	
*Mytilus edulis*	Mytilin A [Swiss-Prot: P81612]	8	Yes	*G+*, G−	[57]
*Mytilus galloprovincialis*	Mytilin B [GenBank: AAD45013]	8	Yes	G+, G−, F	[58, 105]
	Mytilin C [sequence from reference]	7	Yes	G+, G− (F, P)	
	Mytilin D [GenBank: ACF21701]	8	Reverse?	G+, G−, F	
	Mytilin G1 [sequence from reference]	8	Yes	G+ (G−, F)	
*Ascaris suum*	ASABF-α [GenBank: BAA89497]	8	Yes	G+, G− (F)	[59]
*Caenorhabditis elegans*	Ce-ABF2 [NCBI Reference Sequence: NP_491252]	8	Yes	G+, G−, Y	[60]
*Suberites domuncula*	ASABF-related peptide [GenBank: CCC55928]	8	Yes	G+, G−, F, Y, H	[36]
*Caenorhabditis remanei*	Cremycin 5 [GenBank: AEM44806]	6	Yes	F, Y (G+, G−, H)	[21]
	Cremycin 15 [GenBank: AEM44812]	6	Yes	G+, G− (F, Y)	
*Hydra magnipapillata*	Hydramacin [GenBank: ABE26989]	8	Yes	G+, G−	[40, 41]
*Hirudo medicinalis*	Neuromacin [Swiss-Prot: A8V0B3]	8	Yes	G+, G−	[40]
	Theromacin [Swiss-Prot: A8I0L8]	10	Yes	G+, G−	
*Theromyzon tessulatum*	Theromacin [GenBank: AAR12065]	10	Yes	G+ (G−, F)	[39]
*Achatina fulica*	Mytimacin-AF [GenBank: AFR36920]	10	Yes	G+, G−, Y (H)	[44]
*Raphanus sativus*	RsAFP1 [GenBank: AAA69541]	8	Yes	*F* (G+, G−, Y, C, H)	[8, 61]
*Zea mays*	Gamma-2-zeathionin, PDC-1 [Swiss-Prot: P81009]	8	Yes	F	[62, 63]
*Medicago sativa*	MsDEF1 [GenBank: AAG40321]	8	Yes	F	[64–66]
*Nicotiana alata*	NaD1 [Swiss-Prot: Q8GTM0]	8	Yes	F	[67]
*Pentadiplandra brazzeana*	Brazzein [Swiss-Prot: P56552]	8	Yes	G+, G−, Y	[13, 68]
*Pseudoplectania nigrella*	Plectasin [Swiss-Prot: Q53I06]	6	Yes	G+ (G−)	[33]
*Arthroderma otae/ Microsporum canis*	Micasin [GenBank: JN014007]	6	Yes	G+, G− (F, Y, H)	[24]
*Allomyrina dichotoma*	Defensin [Swiss-Prot: Q10745]	6	Yes	G+ (G−)	[74]
*Oryctes rhinoceros*	*O. rhinocerus* defensin [Swiss-Prot: O96049]	6	Yes	G+	[75]
*Copris tripartitus*	Coprisin [GenBank: ABP97087]	6	Yes	G+, G−, Y (H, C)	[76, 106, 107]
*Tenebrio moliter*	Tenecin 1 [Swiss-Prot: Q27023]	6	Yes	*G+* (G−, Y)	[69, 108]
*Haemaphysalis longicornis*	Longicin [Swiss-Prot: Q58A47]	6	Yes	G+, G−, Y, P (H)	[78, 109]
*Nasonia vitripennis*	Navidefensin 2-2 [Sequence from reference]	6	Yes	G+ (G−, F, Y)	[71]
*Homo sapiens*	DLD [GenBank: AK024601]	6	No	F (G+, G−, Y)	[80]

Peptides are listed in the order they are discussed in the text. The activity column lists activity against Gram-positive bacteria (G+), Gram-negative bacteria (G−), filamentous fungi (F), yeast (Y), and protozoa (P), as well as cytotoxic (C) and hemolytic (H) activity. The peptide has the activity shown if the abbreviation is shown without parentheses, and has been tested but not shown to have the activity if shown in parentheses. If a dominant activity has been determined, the abbreviation is shown in italics; any activity not shown has not been tested for that peptide

The first insect defensins reported (sapecin A, phormicin, and royalisin from *Apis mellifera* royal jelly) had six cysteines and were primarily active against Gram-positive bacteria, although results from assays with yeast and fungi were only reported for phormicin [4, 5, 46–49]. *Drosophila* expresses both a six-cysteine defensin with activity against Gram-positive bacteria [50] and the eight-cysteine drosomycin with antifungal activity and similarity to plant defensins (which are predominantly antifungal) [10]. Since insect defensins were thought to be characterized by activity against Gram-positive bacteria, an antifungal peptide from *Heliothis virescens* was named “heliomicin” [46]. However, both gallerimycin and *Galleria* defensin from *Galleria mellonella* show antifungal activity and no antibacterial activity [51, 52]. The situation in arachnids is similar; both Scapularisin 3 and Scapularisin 6 from *Ixodes scapularis* have antifungal activity, but Scapularisin 6 also has activity against Gram-positive bacteria [53]. A defensin from the scorpion *Leiurus quinquestriatus* has activity against Gram-positive but not Gram-negative bacteria [28], while charybdotoxin from the same species has been shown to be active against Gram-positive and Gram-negative bacteria as well as yeast [13]. Therefore, one can deduce little regarding the antimicrobial activity of an arthropod CS-αβ peptide based on the name.

Mollusk peptides also show little correlation between nomenclature and antimicrobial activity. Mollusk defensins, myticins, and mytilins tend to have predominantly Gram-positive activity, but MGD-1 and Myticin B also show some activity against Gram-negative bacteria and fungi [34, 54–56], while Myticin A has shown no additional antimicrobial activity [56]. Mytilins all seem to show activity against Gram-positive bacteria, with mytilins A–D also active against Gram-negative bacteria, and mytilins B and D showing antifungal activity [57, 58]. To the best of my knowledge, antimicrobial activities of mytimacins from mussels have not been published yet. Other macins (hydramacin, neuromcain, theromacin, and mytimacin-AF) have shown primarily antibacterial activity, with antifungal testing being rather limited [39–41, 44].

In nematodes, *Ascaris suum* antibacterial factor (ASABF) has activity against Gram-positive and Gram-negative bacteria [59], while *Caenorhabditis elegans* antibacterial factor 2 (Ce-ABF2) also has activity against yeast [60]. The activity of several additional ABFs in each species has not been reported, including that for the six-cysteine peptide with proposed disulfide bond rearrangement (ASABF-6Cys-α) [38]. The sponge ASABF-like peptide has activity against Gram-positive and Gram-negative bacteria, fungi, yeast, and is hemolytic [36]. Antimicrobial activity has been tested for two of the fifteen cremycins, reported to be drosomycin-type antifungal peptides: cremycin-5 showed antifungal activity, but cremycin-15 showed antibacterial activity without any antifungal activity [21].

Although the primary concern of this study is invertebrate defensins, some invertebrate sequences most closely resemble CS-αβ peptides from plants or fungi. The cysteine number and spacing is much more consistent in plants than in invertebrates and most plant defensins studied have shown antifungal activity; however these peptides are not all called defensins (Additional file 2: Table S1). For example, *Raphanus sativus* antifungal peptide (RsAFP1), *Zea mays* gamma-2-zeathionin (also called PDC-1), *Medicago sativa* defensin 1 (MsDEF1), and *Nicotiana alata* defensin 1 (NaD1) all have antifungal activity [8, 61–67]. Some plant defensins have additional activities against bacteria, oomycetes, or bruchid larvae (Additional file 2: Table S1). Brazzein, initially identified as a sweet-tasting protein from *Pentadiplandra brazzeana* [68], has been shown to have activity against Gram-positive and Gram-negative bacteria as well as yeast [13]. The antimicrobial activity of fungal defensins has only been reported for plectasin and micasin; both have activity against Gram-positive bacteria and micasin is also active against Gram-negative bacteria [24, 33].

If nomenclature based on activity is desirable, then each peptide needs to either be tested extensively prior to reporting or specific antimicrobial activities need to be correlated with sequence features. The γ-core motif has been hypothesized to be a signature of cysteine-rich antimicrobial peptides [13]. Only a few studies have examined the γ-core in isolation, and have shown either antibacterial activity [69, 70] or both antibacterial and antifungal activity [55, 71]. Interestingly, in studies where the fragment was compared to the complete peptide, the isolated γ-core had a greater spectrum of activity than the complete peptide [55, 69, 71]. While the majority of CS-αβ peptides have a γ-core sequence, it is not absolutely necessary for activity. Sapecin B from *Sarcophaga peregrina* does not have a clear γ-core sequence, but has activity against Gram-positive bacteria [72]. An 11-amino acid fragment of sapecin B (7R-17K) upstream of the region corresponding to the γ-core shows activity against not only Gram-positive bacteria, but also Gram-negative bacteria and yeast [73]. The defensins from the beetles *Allomyrina dichotoma, Oryctes rhinoceros,* and *Copris tripartitus* have clear γ-core motifs [74–76], but the fragments studied and found to have antibacterial activity are similar to those from sapecin B [73, 75–77]. Peptides corresponding approximately to these regions of tenecin 1 and longicin do not have antimicrobial activity [69, 78]. Experimental conversion of navidefensin2-2 into a peptide with toxic activity suggested that defensins with the motif KCXN in the γ-core (with C being C6 of the reference array) were likely to have toxic activity if the n-loop is short to prevent steric hindrance during binding to the channel [79]. Consistent with this hypothesis, both charybdotoxin and defensin from *Leiurus* have short n-loops, but charybdotoxin has the sequence “GKCMN” while the defensin has “GYCAG.” Charybdotoxin also has antimicrobial activity, so while the short n-loop and KCXN motif may be sufficient to indicate toxic activity, the characteristics suggesting antimicrobial activity are less clear. Drosomycin-like defensin (DLD) from humans has activity specifically against filamentous fungi, despite the sequence not having conventional CSH, CS-αβ, or γ-core motifs [80].

### Nomenclature does not necessarily reflect phylogeny

The similarity in cysteine pattern and pre-cursor arrangement led to the suggestion that mollusk defensins and nematode ABFs might have a common ancestor [81], while an exon-shuffling mechanism was proposed to explain variability between arthropod and mollusk defensins [82]. Differences in gene structure and the large number of events that would be necessary for exon shuffling to accommodate the nematode sequences led to both the conclusion that convergent evolution was more likely [83] and that there was insufficient evidence to support either model [84]. Rodríguez de la Vega and Possani point out that the lack of defensins reported from basal taxa (such as annelids and merastomatans) and sister groups (including crustaceans, cephalopods, gastropods, and spiders) complicates establishing invertebrate defensins as orthologs [84]. More recently, complete defensin sequences have been reported from five spider species [85] and the gastropod *Haliotis discus* [86].While sequences that look like typical arthropod or mollusk defensins have not been reported from annelids, macins have been reported from the annelids *Hirudo medicinalis* [40] and *Theromyzon tessulatum* [39], as well as from the gastropod *Achatina fulica* [44]. Although not yet characterized, database searches reveal CS-αβ sequences in the crustaceans *Daphnia pulex* and *Litopenaeus vannamei*, the gastropods *Aplysia californica* and *Littorina saxatalis*, and the tardigrades *Hypsibius dujardini* and *Milnesium tardigradum*. As sequencing continues, there is a reasonable expectation that CS-αβ peptides from additional invertebrate taxa will be identified.

The scorpion-toxin like superfamily in the SCOP databases includes both short and long-chain scorpion toxins, insect defensins, plant defensins, and the mollusk defensin MGD-1 [15, 16]. A phylogenetic analysis suggests that the long-chain scorpion sodium channel toxins may have evolved from antifungal defensins [87]. Based on the conserved cysteines and structural information, nematode ABFs and macins are clearly part of this superfamily [41, 88]. Sequences from two myxobacterial species (*A. dehalogenans* and *Stigmatella aurantiaca*) have been identified that may represent the ancestor of the CS-αβ peptides [19]. These sequences have four cysteines that are consistent with the CSH motif, and there is a plausible mechanism for mutations in AdDLP generating the cysteines that form the third disulfide bond of the CS-αβ motif [19]. Testing of recombinant AdDLP has shown no antibacterial or antifungal activity, but has shown activity against *Plasmodium berghei* [20].

**Fig. 2:**
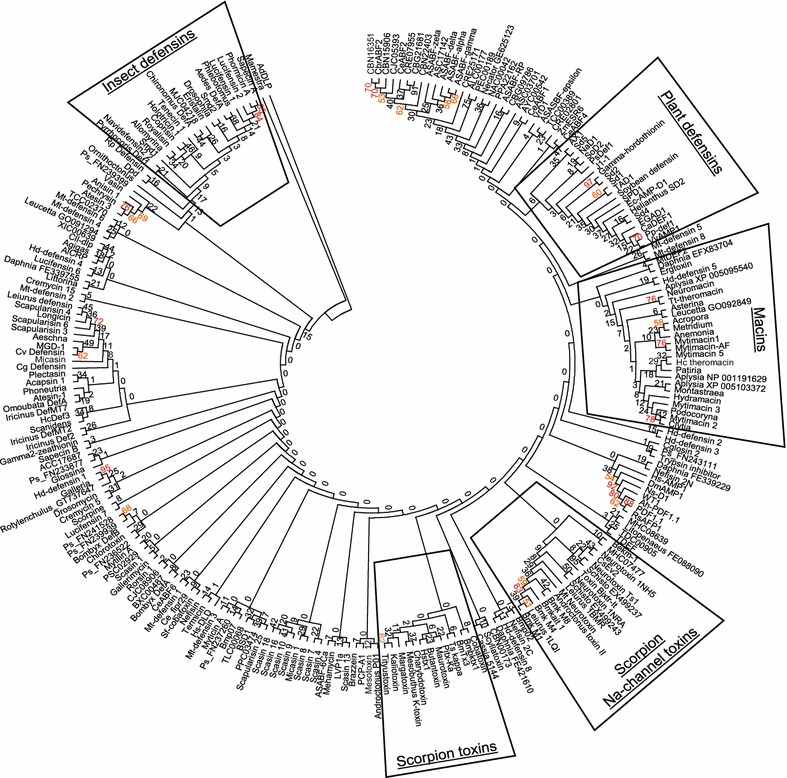
Phylogenetic analyses of 250 CS-αβ peptides. Accession numbers corresponding to the labels in the tree can be found in Additional file 3: Table S2. Bootstrap values greater than 70% are shown in *red font*; those greater than 50% are shown in *orange font*. Bootstrap consensus tree of maximum likelihood analysis in MEGA6 using the WAG + G + I model. *Numbers* reflect the percent support from 1000 bootstrap replicates

Ideally, invertebrate defensins would form a monophyletic group within the superfamily, suggesting that all sequences called “invertebrate defensin” are more closely related to each other than to sequences with other names. Alignments of CS-αβ sequences have to be manually adjusted to ensure the conserved cysteines are accurately positioned, and short sequence length as well as low levels of sequence similarity make it difficult to generate well-resolved trees with well-supported clades. A maximum likelihood phylogenetic analysis of 250 CS-αβ sequences did not produce a well-resolved tree with major clades reflecting taxonomy or nomenclature (Fig. 2, all bootstrap values retained to highlight the low degree of support for the majority of clades). A few small clades were supported at ≥70 (Fig. 2, red bootstrap values). Decreasing the cut-off to ≥50 (orange bootstrap values) added a few more small clades or an additional sequence to a clade ≥70, but did not result in clades defining major groups. There were some identifiable groupings with little to no support, but even these did not necessarily contain all group members previously identified (Fig. 2).

**Fig. 3:**
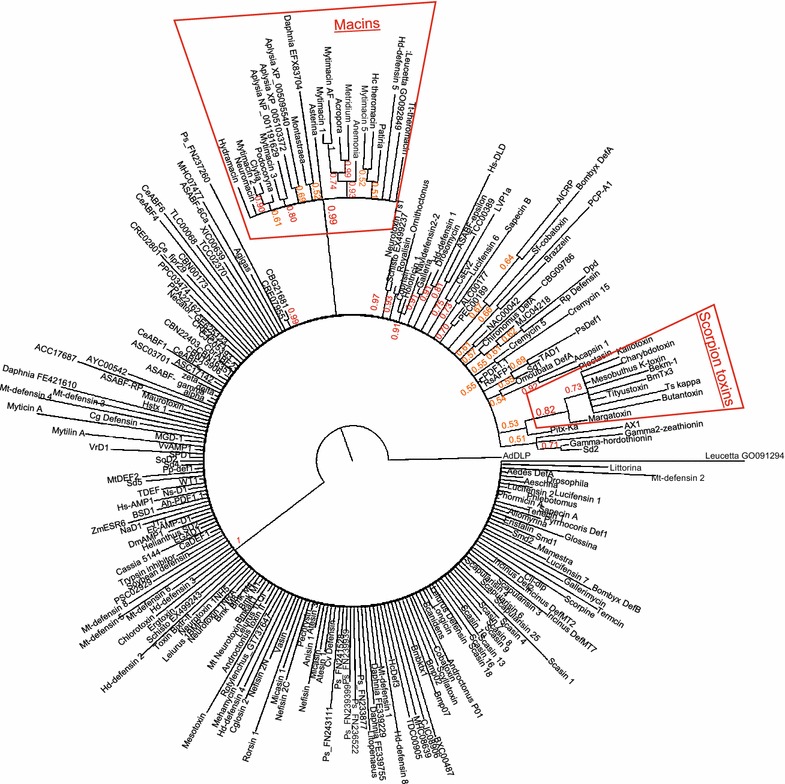
Phylogenetic analyses of 250 CS-αβ peptides. Accession numbers corresponding to the labels in the tree can be found in Additional file 3: Table S2. Posterior probabilities greater than 0.7 are shown in *red font*; those greater than 0.5 are shown in *orange font*. Bayesian analysis in MrBayes 3.2. *Numbers* represent posterior probabilities from an analysis with 2,000,000 generations at a temperature parameter of 0.5

Bayesian analyses of the same dataset also resulted in poorly resolved trees with few well-supported clades, and the runs did not converge (average standard deviation of split frequencies was >0.1; trees not shown). In an effort to increase the phylogenetic signal, a Bayesian analysis was performed using the same set of sequences with added information regarding insertions/deletions (relative to AdDLP) and pro-peptide presence or absence N-/ C-terminal to the mature peptide, an increase in the number of generations, and a decrease in the temperature parameter. These changes did not significantly improve tree resolution and the runs still did not converge (average standard deviation of split frequencies = 0.142989; Fig. 3). This analysis did support the macins as a separate group (Fig. 3, posterior probability = 0.99). The cysteine patterns of two sequences identified in the BLAST searches were most similar to the macin group *(Archispirostreptus gigas* [GenBank: FN197329] and *Peripatopsis sedwicki* [GenBank: FN237260]; Additional file 1: Figure S1); however, their cysteine spacings deviate from those of the majority of macins and the Bayesian analysis did not place them with this group (Fig. 3). The analysis also identified a group of six-cysteine scorpion toxins, although not all six-cysteine scorpion toxin sequences were placed in this clade, and several small groups contained two to four sequences (Fig. 3). Of note, the analysis supported the similarity of drosomycin and human DLD (Fig. 3, posterior probability = 0.91), despite DLD’s lack of signature motifs for this superfamily.

Many papers reporting defensins perform phylogenetic analyses, but most use a limited number of sequences from closely-related species and many do not show measures of support. The analysis arguing for convergent evolution included only ABFs from *A. suum* and *C. elegans*, two insect defensins, one tick defensin, one scorpion sequence, and MGD-1, and showed no measures of support for the resulting clades [83]. A study of defensins from *Ixodes ricinus* included a phylogenetic analysis of sequences from ticks, scorpions, insects, plants, mollusks, and snakes; clades corresponding to these major groups were fairly well supported, with the exception of one scorpion sequence placed in the tick clade and the two mollusk sequences distributed between the insect and scorpion groups [89]. However, no nematode sequences were included, and the rationale for inclusion of 34 snake defensins was unclear since these are not part of the CS-αβ superfamily and are not likely to share a common ancestor with this group.

A recent review classified invertebrate defensins into five categories: arthropod and mollusk-type 6-cysteine defensins, mollusk-type 8-cysteine defensins, nematode-type 8-cysteine defensins, invertebrate big defensins, and invertebrate β-defensin-like peptides [25]. Only the first three categories are part of the CS-αβ superfamily, and while the analysis identifies the major groups, the bootstrap values are low for major clades [25]. Unfortunately, the only nematode sequences included are the ASABFs and CeABFs, which represent only some of the diversity of nematode defensins [90]. Broad analyses such as this are likely to be biased by nomenclature—the analysis included five groups of “defensins,” two of which are not considered to be evolutionarily related, while sequences like drosomycin and the macins were omitted. When studying relationships between invertebrate defensins in the CS-αβ superfamily, sequences from other groups in the superfamily are likely to provide a more appropriate evolutionary context than sequences based on the name “defensin.”

### A reference array for the superfamily may facilitate peptide comparisons

Since there are no clear sequence or activity criteria for classification as an invertebrate defensin, it seems reasonable to consider newly identified CS-αβ sequences in the context of the superfamily. Most sequences in the superfamily have six, eight, or ten cysteines. Using a reference array with ten cysteines, the insect defensins would be described as C2–C6, C3–C8, C4–C9 and the nematode ABFs would be described as C2–C6, C3–C8, C4–C9, C5–C10, making their similarity explicit. Drosomycin and plant defensins would then be described as C2–C6, C3–C8, C4–C9, C1–C10, which clearly shows the difference in the fourth disulfide bridge compared to the nematode ABFs. The ten-cysteine theromacin can then be described as C2–C6, C3–C8, C4–C9, C1–C7, C5–C10. Since the structures of many peptides have not been experimentally verified, bonds inferred from homology can be put in parentheses or brackets. For additional cysteines or cases for which the bonding is not known or inferred, these can be listed in parentheses as well. For example, the structure of mytimacin 5 has not been reported, but from the reference array (Fig. 1) it can be described as (C2–C6, C3–C8, C4–C9, C1–C7, C5–C10, C^3/4^–C^10/^); in this case, the bonding is inferred based on similarity to theromacin, with C^3/4^ located between C3 and C4, and C^10/^ located after C10 that would be hypothesized to form an additional bond.

## Conclusions

It should be possible to clearly define the characteristics of an invertebrate defensin based on invertebrate peptides currently classified as defensins; however, this is not the case. Depending on taxonomic group, a peptide classified as an “invertebrate defensin” may have six or eight cysteines and a variety of biological activities, while a similar (sometimes nearly indistinguishable) peptide with 4–12 cysteines may be called a mycin, macin, mytilin, myticin, antibacterial factor, antifungal peptide, defensin-like peptide/protein, cysteine-rich peptide/protein, or drosomycin-type antifungal peptide. Since it is unlikely that established names of peptides will be changed even if a unified nomenclature is proposed, a reasonable alternative is to establish a system for comparing and discussing these peptides that can be used in addition to peptide names. The proposed reference array clarifies similarities between peptides within the superfamily. Researchers studying invertebrate defensins in the CS-αβ superfamily should be aware that relying exclusively on the term “defensin” to identify sequences for inclusion in phylogenetic analyses has no evolutionary basis, and should instead look at the superfamily for evolutionary context.

## Methods

### CS-αβ sequences used for analysis

CS-αβ sequences used in the analyses were identified from previous BLAST searches of invertebrate taxa performed in this lab, published analyses, and reviews of scorpion toxins [87], nematode CS-αβ sequences [90], plant defensins [91], fungal defensins/defensin-like peptides [23, 24], spider defensins [85], and insect defensins [92] identified in PubMed searches. The query sequences used in BLAST searches are shown in Additional file 4: Table S3. Amino acid sequences were obtained from GenBank and the Protein Database (PDB) available on the NCBI website, Swiss-Prot/Uni-ProtKB [93], NEMBASE4 [94], and WormBase (version WS250, http://www.wormbase.org) [95]. Sequences were viewed in EditSeq (LaserGene 12 Core Suite, DNASTAR, Madison, WI, USA) and saved in FASTA format. If only a nucleotide sequence was available, this was translated in EditSeq; sequences without database entries that were shown in a publication were entered into EditSeq manually. The complete set of sequences considered in the analyses can be found in Additional file 5: Table S4. Structures for Fig. 1 were downloaded from the Molecular Modeling Database (MMDB) [96] and viewed with the Cn3D macromolecular structure viewer (version 4.3.1).

### Phylogenetic analysis

Sequences encoding the mature peptide were aligned in MEGA6 [97] with manual adjustments to ensure accurate alignment of the cysteines. Representatives were chosen from groups of highly similar sequences to reduce the number of sequences for the analyses shown in Fig. 2 and 3. The “Find BestDNA/Protein Models” algorithm identified the WAG + G + I model as the best fit for the data. This model was used for the maximum likelihood analysis with 1000 bootstrap replicates; all sites were used (Fig. 2). Alignments were exported in Nexus (PAUP) format, and the file used as input for MrBayes 3.2 [98]. Since AdDLP is hypothesized as an ancestor of this group, insertions and deletions relative to AdDLP in the full alignment were coded for six areas: (1) AdDLP has three amino acids upstream of C2, so 3aa = 1, <3aa = 0, >3aa = 2; (2) AdDLP has a total of 17 gap spaces between C2 and C3, so 17 = 1, <17 = 0, >17 = 2; (3) AdDLP has three amino acids between C3 and C4 (standard for the motif), so 3aa = 1, <3aa = 0, >3aa = 2; (4) AdDLP has a total of 11 gap spaces between the position of C6 and C8, so 11 = 1, <11 = 0, >11 = 2; (5) AdDLP has one amino acid between C8 and C9 (standard for the motif), so 1aa = 1, <1az = 0, >1aa = 2; and (6) AdDLP has nine amino acids after C9, so 9aa = 1, <9aa = 0, >9aa = 2. N- and C-terminal propeptides were encoded as present = 1 and absent = 0. All missing or unknown data was coded as (-). These data were manually entered into the Nexus file in WordPad. The data was partitioned into protein, indel, and pro(peptide) partitions for analysis. In MrBayes, “lset rates = invgamma” and “prset = aamodel pr = mixed” was applied to the protein partition, “lset rates = gamma” was applied to the indel and pro partitions. The “ratepr” parameter was set to “variable” to allow rates to vary across partitions. For the tree shown in Fig. 3, the number of generations was increased to two million and the temperature decreased to 0.5. Analyses with only one million generations and varying temperature parameters did not give drastically different trees (not shown). The tree was visualized in FigTree1.4 (available from http://tree.bio.ed.ac.uk/software/figtree/) with levels of support shown as posterior probabilities.

## Additional files

**Additional file 1: Figure S1.** Alignment of CS-αβ superfamily sequences with reference array. Alignment to a ten-cysteine reference array based on the cysteines forming the CSH motif (C3–C8, C4–C9) and the third bond completing the CS-αβ fold (C2–C6). The GXC of the γ-core is usually at C6, except for mytilins (C7). A filled square indicates presence of the indicated cysteine and numbers in unfilled blocks represent the number of intervening amino acids. Sequences are grouped based on the basic cysteine patterns observed and within these by major taxonomic group (listed in column A). Additional cysteines at the N or C-terminus of the conserved array are represented by additional filled boxes. Additional cysteines within the conserved array are represented as “C.” Major taxonomic groups are color-coded: Annelida (dark rose), Arachnida (light orange), Bacteria (black), Bivalvia (light blue), Cnidaria (light grey), Crustacea (peach), Echinodermata (pink), Fungi (light green), Gastropoda (blue), Hexapoda (orange), Myriapoda (dark orange), Nematoda (lavender), Onychophora (light purple), Plantae (green), Platyhelminthes (light rose), Porifera (dark grey), and Tardigrada (yellow).

**Additional file 2: Table S1.** Antimicrobial activity of CS-αβ peptides. Peptides are listed in alphabetical order by species within major taxonomic groups. The activity column lists activity against Gram-positive bacteria (G+), Gram-negative bacteria (G−), filamentous fungi (F), yeast (Y), and protozoa (P), as well as cytotoxic (C) and hemolytic (H) activity. The peptide has the activity shown if the abbreviation is shown without parentheses, and has been tested but not shown to have the activity if shown in parentheses. If a dominant activity has been determined, the abbreviation is shown in bold; any activity not shown has not been tested for that peptide.

**Additional file 3: Table S2.** Representative CS-αβ sequences used for phylogenetic analysis. Accession numbers are shown for each sequence used in the analysis. The “Tree Label” column corresponds to the names shown in Fig. 2 and 3. The “Sequences Represented” column shows information for sequences excluded from the analysis based on similarity to the representative sequence. In cases where a sequence is represented by more than one accession number, the sequence used is shown in bold.

**Additional file 4: Table S3.** Accession numbers for query sequences used in BLAST searches.

**Additional file 5: Table S4.** CS-αβ sequences considered in this study. In cases where a sequence is represented by more than one accession number, the sequence used is shown in bold.

## Abbreviations

CS-αβ: cysteine-stabilized alpha-beta
CSH: cysteine-stabilized alpha-helix
