# Supplementary material for: Establishing a reference array for the CS-αβ superfamily of defensive peptides
Source: BMC Res Notes. 2016 Nov 18;9:490. doi: 10.1186/s13104-016-2291-0 (PMC5116183; doi:10.1186/s13104-016-2291-0)
Supplement: Supplementary file 2 — Additional file 2: Table S1. Antimicrobial activity of CS-αβ peptides. Peptides are listed in alphabetical order by species within major taxonomic groups. The activity column lists activity against Gram-positive bacteria (G+), Gram-negative bacteria (G−), filamentous fungi (F), yeast (Y), and protozoa (P), as well as cytotoxic (C) and hemolytic (H) activity. The peptide has the activity shown if the abbreviation is shown without parentheses, and has been tested but not shown to have the activity if shown in parentheses. If a dominant activity has been determined, the abbreviation is shown in bold; any activity not shown has not been tested for that peptide. [file 13104_2016_2291_MOESM2_ESM.pdf]

Table S1. Antimicrobial activity of CS-αβ peptides. Peptides are listed in alphabetical order by species within major taxonomic groups. The activity column lists activity against Gram-positive bacteria (G+), Gram-negative bacteria (G-), filamentous fungi (F), yeast (Y), and protozoa (P), as well as cytotoxic (C) and hemolytic (H) activity. The peptide has the activity shown if the abbreviation is shown without parentheses, and has been tested but not shown to have the activity if shown in parentheses. If a dominant activity has been determined, the abbreviation is shown in bold; any activity not shown has not been tested for that peptide.

**Species (by major taxonomic group)**	**Name [Accession number]**	**# C**	**γ-core**	**Antimicrobial activity**	**Notes**	**References**
**Bacteria**						
Anaeromyxobacter dehalogenans	AdDLP [NCBI Reference Sequence: WP_011422871]	4	No	P (G+, G-, F, Y, H)	Defensin-like peptide	[1, 2]
**Cnidaria**						
Hydra magnipapillata	Hydramacin [GenBank: ABE26989]	8	Yes	G+, G-	Macin	[3, 4]
**Porifera**						
Suberites domuncula	ASABF-related peptide [GenBank: CCC55928]	8	Yes	G+, G-, F, Y, H	Antibacterial factor	[5]
**Insect**						
Acalolepta luxuriosa	AlCRP [GenBank: AB104817]	6	Yes	G+, G-	Cysteine-rich peptide	[6]
Aedes aegypti	Defensin A [Swiss-Prot: P91793]	6	Yes	G+, G- (Y)	Insect defensins	[7-9]
	Defensin B [Swiss-Prot: P81602]	6	Yes	G+ (G-, Y)		
Aeschna cyanea	*Aeschna* defensin [Swiss-Prot: P80154]	6	Yes	**G+**, G-	Insect defensin from a Paleopteran (evolutionarily more ancient than dipterans and coleopterans)	[10]
Allomyrina dichotoma	*A. dichotoma* defensin [Swiss-Prot: Q10745]	6	Yes	G+ (G-)	Insect defensin	[11]
Anomala cuprea	Defensin A [Swiss-Prot: P83669]	6	Yes	G+ (G-)	Insect defensin	[12]
	Defensin B [Swiss-Prot: P83668]	6	Yes	G+, G-		
Anopheles gambiae	Defensin [Swiss-Prot: Q17027]	6	Yes	G+, G-, F (Y)	Insect defensin	[13, 14]

Apis mellifera	Royalisin [Swiss-Prot: P17722]	6	Yes	**G+**, G-	Initial report noted similarity to sapecin and phormicin	[15, 16]
Archeoprepona demophon	ARD1 [PDB: 1OZZ_A]	6	Yes	F, Y	Insect defensin	[17]
Bombus pascuorum	*B. pascuorum* defensin [Swiss-Prot: P81462]	6	Yes	G+, G-, F	Insect defensin	[18]
Chironomus plumosus	Defensin A [Sequence from reference]	6	Yes	G+ (G-)	Insect defensin	[19]
Copris tripartitus	Coprisin [GenBank: ABP97087]	6	Yes	G+, G-, Y (H, C)	Insect defensin	[20-22]
Drosophila melanogaster	*Drosophila* defensin [Swiss-Prot: P36192]	6	Yes	G+	Insect defensin	[23]
	Drosomycin [Swiss-Prot: P41964]	8	Yes	**F**, Y, P (G+, G-, H)	Noted similarity to plant antifungal peptides	[24, 25]
	Drosomycin-2 [GenBank: ABY84135]	8	Yes	F, Y, P (G+, G-)	Drosomycin, antifungal defensin	[25]
Formica rufa	*F. rufa* defensin [Sequence from reference]	6	Yes	G+ (G-, F)	Insect defensin	[26]
Galleria mellonella	Gallerimycin [Swiss-Prot: Q8MVY9]	6	Yes	F (G+, G-, Y)	Defensin-like peptide	[27]
	Galleria defensin [Swiss-Prot: P85213]	6	Yes	F, Y (G+, G-)	Insect defensin	[28]
Glossina morsitans	Defensin-A [Swiss-Prot: Q8WTD4]	6	Yes	G+	Insect defensin	[29, 30]
Heliothis virescens	Heliomicin [GenBank: ACR78445]	6	Yes	**F**, Y (G+, G-)	Insect defensin	[31]
Holotrichia diomphalia	Holotricin-1 [Swiss-Prot: Q7M426]	6	Yes	**G+** (G-)	Sapecin homologue (insect defensin)	[32]
Lucilia sericata	Lucifensin2 [GenBank: ADI87383]	6	Yes	G+, Y (G-)	Insect defensin	[33-36]
	Lucifensin1 [Sequence from reference]	6	Yes	(G+, G-)	Insect defensins	[36]
	Lucifensin3 [Sequence from reference]	6	Yes	G+ (G-)		
	Lucifensin4 [Sequence from reference]	6	Yes	G+ (G-)		

	Lucifensin6<br>[Sequence from reference]	6	Yes	G+ (G-)		
	Lucifensin7<br>[Sequence from reference]	6	Yes	G+ (G-)		
Mamestra brassicae	*Mamestra* defensin<br>[GenBank: AAL69980]	6	No	G+, G- (Y)	Insect defensin	[37]
Nasonia vitripennis	Navidefensin2-2<br>[Sequence from reference]	6	Yes	G+ (G-, F, Y)	Insect defensin	[38]
Oryctes rhinoceros	*O. rhinocerus* defensin<br>[Swiss-Prot: O96049]	6	Yes	G+	Insect defensin, some fragments had antibacterial activity, but no hemolytic or cytotoxic activity	[39]
Phlebotomus duboscqi	*Phlebotomus* defensin<br>[Swiss-Prot: P83404]	6	Yes	G+, F, Y, P	Insect defensin	[40]
Protophormia terraenovae	Phormicin, *Phormia* defensin A<br>[Swiss-Prot: P10891]	6	Yes	**G+**, G-, F (Y)	1989 paper proposed the term "insect defensin"	[31, 41, 42]
	Phormia defensin B<br>[Swiss-Prot: P10891 (G86R)]	6	Yes	G+		
Pseudacanthotermes spiniger	Termicin<br>[Swiss-Prot: P82321]	6	No	F, Y, G+ (G-)	Insect defensin	[42]
Pyrrhocoris apterus	*Pyrrhocoris* defensin 1<br>[Swiss-Prot: P37364]	6	Yes	**G+**, G-	Insect defensin	[43]
Sarcophaga peregrina	Sapecin, Sapecin A<br>[Swiss-Prot: P18313]	6	Yes	**G+**, G-	Similarity to mammalian defensins noted, but not called a defensin in initial report; later referred to as defensins	[44-47]
	Sapecin B<br>[Swiss-Prot: P31529]	6	No	**G+** (G-, Y)		
	Sapecin C<br>[Swiss-Prot: P31530]	6	Yes	**G+** (G-)		
Spodoptera littoralis	SpliDef<br>[GenBank: HQ603825]	6	Yes	G+, G-	Insect defensin	[48]
Stomoxys calcitrans	Smd1 [Swiss-Prot: O16136]	6	Yes	G-	Insect defensins	[49]
	Smd2 [Swiss-Prot: O16137]	6	Yes	G-		
Tenebrio moliter	Tenecin 1<br>[Swiss-Prot: Q27023]	6	Yes	**G+** (G-, Y)	Similar to sapecin	[50, 51]
Zophobas atratus	*Zophobas* Peptide B<br>[GenBank: AAB27045]	6	Yes	G+	Insect defensins from Coleoptera	[52]

	Zophobas Peptide C [GenBank: AAB27046]	6	Yes	G+, G-		
**Arachnid**						
Centruroides limpidus	Cll-dlp [Swiss-Prot: Q6GU94]	6	Yes	G+, G-	Defensin-like peptide, activity only in high concentrations and in cooperation with other fractions	[53]
Centruroides sculpturatus	CsEv2, Neurotoxin 2 [Swiss-Prot: P01493]	8	Yes	NT	Causes paralysis in crickets	[54]
Dermacentor variabilis	Varisin A1 [Swiss-Prot: Q86QI5]	6	Yes	G+, *B. burgdorferi*	Arthropod defensin	[55]
Haemaphysalis longicornis	Longicin [Swiss-Prot: Q58A47]	6	Yes	G+, G-, Y, P (H)	Arthropod defensin	[56, 57]
Ixodes ricinus	I. ricinus defensin1 [GenBank: AAP94724]	6	Yes	G+ (G-, Y, V, H)	Arthropod defensins	[58, 59]
	I. ricinus defensin2 [GenBank:ABC88432]	6	Yes	G+ (G-, Y, V, H)		
	DefMT2 [GenBank: JAA65352]	6	Yes	(G+, G-, F)		[60, 61]
	DefMT3 [GenBank: JAA71488]	6	Yes	G+, G-, F		
	DefMT5 [GenBank: JAA66832]	6	Yes	G+, G-, F		
	DefMT6 [GenBank: JAA71516]	6	Yes	G+, G-, F		
	DefMT7 [GenBank: JAA69779]	6	Yes	(G+, G-, F)		
Ixodes scapularis	Scapularisin (Scapularisin 6) [GenBank: AAV74387]	6	Yes	G+, F (G-)	Arthropod defensin	[62, 63]
	Scapularisin 3 [GenBank: EEC13914]	6	Reverse?	F (G+, G-)	Arthropod defensin	[63, 64]
Leiurus quinquestriatus	Charybdotoxin [Swiss-Prot: P13487]	6	Yes	G+, G-, Y	Inhibits calcium-activated potassium channels	[65-67]

	Scorpion defensin [Swiss-Prot: P41965]	6	Yes	G+ (G-)	Similarity to both insect defensins and scorpion toxins noted; ability of scorpion to produce both a toxin and a defensin	[68]
Mesobuthus martensii	Potassium channel toxin [Swiss-Prot: Q9NII6]	6	Yes	NT	Acts on potassium channels; neurotoxic in mice	[69]
Ornithoctonus hainana	Defensin [Sequence from reference]	6	Yes	G+, G-, Y	Arthopod defensin	[70]
Ornithodoros moubata	Defensin A [Swiss-Prot: Q9BLJ3]	6	Yes	G+ (G-, H)	Arthropod defensin	[71, 72]
Pandinus imperator	Scorpine [Swiss-Prot: P56972]	6	Yes	G+, G-, P (H)	N-term cecropin, C-term insect defensin	[73, 74]
**Nematode**						
Ascaris suum	ASABF-α [GenBank: BAA89497]	8	Yes	G+, G- (F)	Antibacterial factor	[75]
Caenorhabditis elegans	Ce-ABF2 [NCBI Reference Sequence: NP_491252]	8	Yes	G+, G-, Y	Antibacterial factor	[76]
Caenorhabditis remanei	Cremycin 5 [GenBank: AEM44806]	6	Yes	F, Y (G+, G-, H)	Drosomycin-type antifungal peptides	[77]
	Cremycin 15--mislabeled as 14 [GenBank: AEM44812]	6	Yes	G+, G- (F, Y)		
**Mollusk**						
Achatina fulica	Mytimacin-AF [GenBank: AFR36920]	10	Yes	G+, G-, Y (H)	Macin	[78]
Crassostrea gigas	Cg-defensin [GenBank: AJ565499]	8	Yes	**G+**, G-, F	Member of arthropod defensin family, mollusk defensin	[79]
Crassostrea virginica	American oyster defensin, AOD [Swiss-Prot: P85008]	6	Yes	G+, G-	Member of arthropod defensin family, mollusk defensin	[80]
Dreissena polymorpha	Dpd [GenBank: ACZ02692]	6	Yes	G+, G- (Y)	Mollusk defensin	[81]
Mytilus edulis	Mytilin A [Swiss-Prot: P81612]	8	Yes	**G+**, G-	Mytilins proposed as a different group based on position of cysteines in primary structure	[82]

Mytilus galloprovincialis	MGD-1 [Swiss-Prot: P80571]	8	Yes	G+, G-, F (C)	Member of arthropod defensin family, mollusk defensin; some fragments active against yeast or protozoa	[83-87]
	Myticin A [Swiss-Prot: P82103]	8	No	G+ (G-, F, P)	Myticins proposed as a different group based on position of cysteines in primary structure	[88]
	Myticin B [Swiss-Prot: P82102]	8	No	G+, G-, F (P)		
	Mytilin B [GenBank: AAD45013]	8	Yes	G+, G-, F	Mytilins	[89, 90]
	Mytilin C [Sequence from reference]	7	Yes	G+, G- (F, P)		
	Mytilin D [GenBank: ACF21701]	8	Reverse?	G+, G-, F		
	Mytilin G1 [Sequence from reference]	8	Yes	G+ (G-, F)		
**Annelid**						
Hirudo medicinalis	Neuromacin [Swiss-Prot: A8V0B3]	8	Yes	G+, G-	Macins	[4]
	Theromacin [Swiss-Prot: A8I0L8]	10	Yes	G+, G-		
Theromyzon tessulatum	Theromacin [GenBank: AAR12065]	10	Yes	G+ (G-, F)	Macin	[91]
**Plant**						
Aesculus hippocastanum	Ah-AMP1 [GenBank: AAB34970]	8	Yes	**F,** G+ (G-)	Plant defensin	[92]
Arabidopsis halleri	Ah-PDF1.1 [GenBank: AAY27736]	8	Yes	G+, F (G-, Y)	Plant defensin; confers zinc tolerance	[93, 94]
Arabidopsis thaliana	At-AFP, PDF1.1 [NCBI Reference Sequence: NP_565119]	8	Yes	**F**, Y (G+, G-)	New family of basic cysteine-rich plant antifungal proteins from Brassicaceae species	[95, 96]
	PDF1.2 [Swiss-Prot: Q9FI23]	8	Yes	F	Plant defensin	[97]
	PDF1.3 [NCBI Reference Sequence: NP_180171]	8	Yes	**F**, Y	Plant defensin	[96]
Beta vulgaris	AX1 [Swiss-Prot: P81493]	8	Yes	F (G+, G-)		[98]

	AX2 [Swiss-Prot: P82010]	8	Yes	F (G+, G-)	Antifungal cysteine-rich proteins, related to gamma-thionins	
Brassica campestris	BSD1 [Sequence from reference]	8	No	F, O	Plant defensin	[99]
Capsicum annuum	J1-1 [Swiss-Prot: Q43413]	8	Yes	F	Plant defensin	[100]
	CaDEF1 [GenBank: AAL35366]	8	Yes	F	Plant defensin	[101]
Capsicum chinense	gamma-thionin [GenBank: AAD21200]	8	Yes	Y, C	Plant defensin, thionin	[102]
Clitoria ternatea	Ct-AMP1 [GenBank: AAB34971]	8	Yes	**F,** G+ (G-)	Plant defensin	[92]
Dahlia merckii	Dm-AMP1 [GenBank: AAB34972]	8	Yes	**F,** G+ (G-)	Plant defensin	[92]
Echinocloa crusgalli	Ec-AMP-D1 [Swiss-Prot: P86518]	8	Yes	F, O	Plant defensin	[103]
	Ec-AMP-D2 [Sequence from reference]	8	Yes	F, O		
Hardenbergia violacea	HvAMP1 [Sequence from reference]	8	Yes	F, O (G+, G-, Y, C, H)	Plant defensin	[104]
Helianthus annuus	HaDEF1 [GenBank: AF364865]	8	Yes	F, Y, *Orobanche*	Plant defensin	[105]
Heuchera sanguinea	Hs-AMP1 [Swiss-Prot: P0C8Y5]	8	Yes	**F** (G+, G-)	Plant defensin	[92]
Ipomoea batatas	SPD1 [GenBank: AY552546]	8	Yes	G+, F (G-)	Plant defensin	[106]
Lens culinaris	LcDef [GenBank: ABP04037]	8	Yes	F	Plant defensin	[107]
Lepidium meyenii	Lm-def [GenBank: AAV85992]	8	Yes	O	Plant defensin	[108]
Medicago sativa	MsDEF1 [GenBank: AAG40321]	8	Yes	F	Plant defensin, blocks mammalian L-type Ca2+ channels; inhibits root growth	[109-111]
Mendicago trucatula	MtDEF2 [GenBank: AAQ91290]	8	Yes	F	Plant defensin, inhibits root growth, doesn't block mammalian L-type Ca2+ channels	[110, 111]
Nicotiana alata	NaD1 [Swiss-Prot: Q8GTM0]	8	Yes	F	Plant defensin	[112]
Nicotiana megalosiphon	Nm-Def02 [GenBank: ACR46857]	8	Yes	F, O	Plant defensin	[113]
Nigella sativa	Ns-D1 [Swiss-Prot: P86972]	8	Yes	G+, G-, F, O	Plant defensins	[114]

	Ns-D2 [Swiss-Prot: P86973]	8	Yes	G+, G-, F, O		
Pachyrrhizus erosus	SPE10 [GenBank: AAT80338]	8	Yes	F	Plant defensin	[115, 116]
Pentadiplandra brazzeana	Brazzein [Swiss-Prot: P56552]	8	Yes	G+, G-, Y	Sweet tasting protein	[67, 117]
Petunia hybrida	PhD1 [Swiss-Prot: Q8H6Q1]	10	Yes	F	Plant defensins	[112]
	PhD2 [Swiss-Prot: Q8H6Q0]	10	Yes	F		
Phaseolus limensis	BLBAFP [Sequence from reference]	8	Yes	F	Plant defensin	[118]
Phaseolus vulgaris	WCBAFP [Sequence from reference]	8	Yes	G+, G-, F	Plant defensin	[119]
	PvD1 [GenBank: ADR30066]	8	Yes	F, Y	Plant defensin	[120]
Pinus sylvestris	PsDef1 [Swiss-Prot: A4L7R7]	8	Yes	F, Y (G-)	Plant defensin	[121, 122]
Pisum sativum	PsD1 [Swiss-Prot: P81929]	8	Yes	F (Y)	Plant defensins	[123]
	PsD2 [Swiss-Prot: P81930]	8	Yes	F (Y)		
Prunus persica	Pp-def1 [GenBank: AAL85480]	8	Yes	F (G-)	Plant defensin	[124]
Raphanus sativus	RsAFP1 [GenBank: AAA69541]	8	Yes	**F** (G+, G-, Y, C, H)	Report noted that RsAFPs belonged to a superfamily of small, basic, cysteine-rich proteins with antibacterial activity (including insect defensins), but were unique due to their antifungal activity	[92, 125]
	RsAFP2 [GenBank: AAA69540]	8	Yes	**F**, G+ (G-, Y, C, H)		
Saccharum officinarum	Sd1 [GenBank: CA112870]	8	Yes	F (G+, G-)	Plant defensins	[126]
	Sd3 [GenBank: CA259771]	8	Yes	F (G+, G-)		
	Sd5 [GenBank: CA297803]	8	Yes	F (G+, G-)		
Solanum lycopersicum	DEF2, clone cTOB11C9 [GenBank: AW623541]	8	Yes	F	Plant defensin	[127]
Spinacea oleracea	SoD2 [Swiss-Prot: P81571]	8	Yes	G+, G-, F	Plant defensin	[128]
Stellaria media	Sm-AMP-D1 [Sequence from reference]	8	Yes	F, O	Plant defensin	[129]
Tephrosia villosa	TvD1 [GenBank: AAX86993]	8	Yes	**F**	Plant defensin	[130]
Trichosanthes kirilowii	TDEF [GenBank: ABF74600]	8	Yes	F	Plant defensin	[131]
Trigonella foenum-graecum	Tfgd1 [GenBank: AAO72632]	8	Yes	F	Plant defensin	[132]
Triticum aestivum	TAD1 [GenBank: BAC10287]	8	Yes	G-	Plant defensin	[133]

Vigna angularis	VaD1, azuki bean defensin [Sequence from reference]	8	Yes	G+, G-, F, Bruchid larvae	Plant defensin	[134]
Vigna radiata	VrCRP [Sequence from reference]	8	Yes	Bruchid larvae	Plant defensin	[135]
	VrD1, mungbean defensin [GenBank: AAR08912]	8	Yes	F, bruchid larvae (G-)	Plant defensin	[136]
Vigna unguiculata	Cp-thionin II [Sequence from reference]	8	Yes	G+, G-	Plant defensin, thionin	[137]
Vitis vinifera	Vv-AMP1 [Sequence from reference]	8	Yes	F	Plant defensin	[138]
Wasabia japonica	WT1 [GenBank: AB012871]	8	Yes	F, G-	Plant defensin	[139]
Zea mays	Gamma-2-zeathionin, PDC-1 [Swiss-Prot: P81009]	8	Yes	F	Plant defensin, thionin	[140, 141]
Zea mays	ZmESR-6 [NCBI Reference Sequence BP_001105777]	8	Yes	G+, G-, F	Plant defensin	[142]
**Fungi**						
Arthroderma otae/ Microsporum canis	Micasin [GenBank: JN014007]	6	Yes	G+, G- (F, Y, H)	Defensin-like peptide	[143]
Pseudoplectania nigrella	Plectasin [Swiss-Prot: Q53I06]	6	Yes	G+ (G-)	Fungal defensin	[144]
**Human**						
Homo sapiens	DLD [GenBank: AK024601]	6	No	F (G+, G-, Y)	Drosomycin-like defensin	[145]

References

1. Zhu S. Evidence for myxobacterial origin of eukaryotic defensins. Immunogenetics. 2007 Dec.;59(12):949-54.
2. Gao B, del Carmen Rodriguez M, Lanz-Mendoza H, Zhu S. AdDLP, a bacterial defensin-like peptide, exhibits anti-*Plasmodium* activity. Biochem Biophys Res Commun. 2009;387:393-8.
3. Jung S, Dingley AJ, Augustin R, Anton-Erxleben F, Stanisak M, Gelhaus C, et al. Hydramacin-1, Structure and Antibacterial Activity of a Protein from the Basal Metazoan *Hydra* J Biol Chem. 2009 January 16;284(3):1896-905.
4. Jung S, Sönnichsen FD, Hung C-W, Tholey A, Boidin-Wichlacz C, Haeusgen W, et al. Macin Family of Antimicrobial Proteins Combines Antimicrobial and Nerve Repair Activities. Journal of Biological Chemistry. 2012;287(17):14246-58.
5. Wiens M, Schröder HC, Korzhev M, Wang X-H, Batel R, Müller WEG. Inducible ASABF-type antimicrobial peptide from the sponge *Suberites domuncula:* Microbicidal and hemolytic activity *in vitro* and toxic effect on molluscs *in vivo.* Mar Drugs. 2011;9(10):1969-94. Epub Oct 19.

6. Saito A, Ueda K, Imamura M, Miura N, Atsumi S, Tabunoki H, et al. Purification and cDNA cloning of a novel antibacterial peptide with a cysteine-stabilized ab motif from the longicorn beetle, *Acalolepta luxuriosa*. Developmental and Comparative Immunology. 2004 January;28(1):1-7.
7. Lowenberger C, Bulet P, Charlet M, Hetru C, Hodgeman B, Christensen BM, et al. Insect Immunity: Isolation of Three Novel Inducible Antibacterial Defensins from the Vector Mosquite, *Aedes aegypti*. Insect Biochem Molec Biol. 1995 July;25(7):867-91.
8. Chalk R, Albuquerque CMR, Ham PJ, Townson H. Full sequence and characterization of two insect defensins: immune peptides from the mosquito *Aedes aegypti.* Proc R Soc Lond B. 1995 Aug. 22;261(1361):217-21.
9. Lowenberger CA, Smartt CT, Bulet P, Ferdig MT, Severson DW, Hoffmann JA, et al. Insect immunity: molecular cloning, expression, and characterization of cDNAs and genomic DNA encoding three isoforms of insect defensin in *Aedes aegypti*. Insect Molecular Biology. 1999 Feb.;8(1):107-18.
10. Bulet P, Cociancich S, Reuland M, Sauber F, Bischoff R, Hegy G, et al. A novel insect defensin mediates the inducible antibacterial activity in larvae of the dragonfly *Aeschna cyanea* (Paleoptera, Odonata). Eur J Biochem. 1992;209:977-84.
11. Miyanoshita A, Hara S, Sugiyama M, Asaoka A, Taniai K, Yukuhiro F, et al. Isolation and Characterization of a New Member of the Insect Defensin Family from a Beetle, *Allomyrina dichotoma*. Biochemical an Biophysical Research Communications. 1996;220(3):526-31.
12. Yamauchi H. Two novel insect defensins from larvae of the cupreous chafer, *Anomala cuprea:* purification, amino acid sequences and antibacterial activity. Insect Biochem Molec Biol. 2001 Dec;32(1):75-84.
13. Richman AM, Bulet P, Hetru C, Barillas-Mury C, Hoffmann JA, Kafatos FC. Inducible immune factors of the vector mosquito *Anopheles gambiae:* biochemical purification of a defensin antibacterial peptide and molecular cloning of a preprodefensin cDNA. Insect Molecular Biology. 1996 Aug.;5(3):203-10.
14. Vizioli J, Richman AM, Uttenweiler-Joseph S, Blass C, Bulet P. The defensin peptide of the malaria vector mosquito *Anopheles gambiae:* antimicrobial activities and expression in adult mosquitoes. Insect Biochem Molec Biol. 2001;31:241-8.
15. Fujiwara S, Imai J, Fujiwara M, Yaeshima T, Kawashima T, Kobayashi K. A potent antibacterial protein in royal jelly. Purification and determination of the primary structure of royalisin. Journal of Biological Chemistry. 1990;285(19):11333-7.
16. Bílikova K, Huang S-C, Lin I-P, Šimuth J, Peng C-C. Structure and antimicrobial activity relationship of royalisin, an antimicrobial peptide from royal jelly of *Apis mellifera.* Peptides. 2015.
17. Landon C, Barbault F, Legrain M, Menin L, Guenneugues M, Schott V, et al. Lead optimization of antifungal peptides with 3D NMR structures analysis. Protein Science. 2004 March;13(3):703-13.
18. Rees JA, Moniatte M, Bulet P. Novel Antibacterial Peptides Isolated from a European Bumblebee, *Bombus pascuorum* (Hymenoptera, Apoidea). Insect Biochem Molec Biol. 1997 May;27(5):413-22.
19. Lauth X, Nesin A, Briand J-P, Roussel J-P, Hetru C. Isolation, characterization and chemical synthesis of a new insect defensin from *Chironomus plumosus* (Diptera). Insect Biochem Molec Biol. 1998 December;28(12):1059-66.
20. Hwang J-S, Lee J, Kim Y-J, Bang H-S, Yun E-Y, Kim S-R, et al. Isolation and Characterization of a Defensin-Like Peptide (Coprisin) from the Dung Beetle, *Copris tripartitus*. International Journal of Peptides. 2009;2009.
21. Lee E, Kim J-K, Shin S, Jeong K-W, Shin A, Lee J, et al. Insight into the antimicrobial activities of coprisin isolated from the dung beetle, *Copris tripartitus,* revealed by structure-activity relationships. Biochimica et Biophysica Acta. 2013 Feb. ;1282(2):271-83.

22. Lee J, Lee D, Choi H, Kim HH, Kim H, Hwang JS, et al. Structure-activity relationships of the intramolecular disulfide bonds in coprisin, a defensin from the dung beetle. BMB Reports. 2014 Nov.;47(11):625-30.
23. Dimarcq J-L, Hoffmann D, Meister M, Bulet P, Lanot R, Reichhart J-M, et al. Characterization and transcriptional profiles of a *Drosophila* gene encoding an insect defensin. Eur J Biochem. 1994 April 1;221(1):201-9.
24. Fehlbaum P, Bulet P, Michaut L, Lagueux M, Broekaert WF, Hetru C, et al. Septic injury of Drosophila induces the synthesis of a potent antifungal peptide with sequence homology to plant antifungal peptides. Journal of Biological Chemistry. 1994;269(52):33159-63.
25. Tian C, Gao B, del Carmen Rodriguez M, Lanz-Mendoza H, Ma B, Zhu S. Gene expression, antiparasitic activity, and functional evolution of the drosomycin family. Molecular Immunology. 2008 Sept.;45(15):3909-16.
26. Taguchi S, Bulet P, Hoffmann JA. A novel insect defnsin from the ant *Formica rufa*. Biochimie. 1998 April;80(4):343-6.
27. Schuhmann B, Seitz V, Vilcinskas A, Podsiadlowski L. Cloning and Expression of Gallerimycin, an Antifungal Peptide Expressed in Immune Response of Greater Wax Moth Larve, *Galleria mellonella.* Archives of Insect Biochemistry and Physiology. 2003 July;53(3):125-33.
28. Lee YS, Yun EK, Jang WS, Kim I, Lee JH, Park SY, et al. Purification, cDNA cloning and expression of an insect defensin from the great wax moth, *Galleria mellonella.* Insect Molecular Biology. 2004 Feb.;13(1):65-72.
29. Hao Z, Kasumba I, Lehane MJ, Gibson WC, Kwon J, Aksoy S. Tsetse immune responses and trypanosome transmission: Implications for the development of tsetse-based strategies to reduce trypanosomiasis. PNAS. 2001 October 23, 2001;98(22):12648-53.
30. Boulanger N, Brun R, Ehret-Sabatier L, Kunz C, Bulet P. Immunopeptides in the defense reactions of *Glossina morsitans* to bacterial and *Trypanosoma brucei brucei* infections. Insect Biochem Molec Biol. 2002 April;32(4):369-75.
31. Lamberty M, Ades S, Uttenweiler-Joseph S, Brookhart G, Bushey D, Hoffmann JA, et al. Isolation from the lepidopteran *Heliothis virescens* of a novel insect defensin with potent antifungal activity. Journal of Biological Chemistry. 1999 April 2;274(14):9320-6.
32. Lee SY, Moon HJ, Kawabata S-i, Kurata S, Natori S, Lee BL. A Sapecin Homologue of *Holotrichia diomphalia:* Purification, Sequencing and Determination of Disulfide Pairs. Biological and Pharmaceutical Bulletin. 1995 March;18(3):457-9.
33. Andersen AS, Sandvang D, Schnorr KM, Kruse T, Neve S, Joergensen B, et al. A novel approach to the antimicrobial activity of maggot debridement therapy. Journal of Antimicrobial Chemotherapy. 2010 August;65(8):1646-54.
34. Čeřovský V, Ždárek J, Fučík V, Monincová L, Voburka Z, Bém R. Lucifensin, the long-sought antimicrobial factor of medicinal maggots of the blowfly *Lucilia sericata*. Cell Mol Life Sci. 2010 Feb. ;67(3):455-66.
35. Čeřovský V, Slaninová J, Fučík V, Monincová L, Bednárová L, Maloň P, et al. Lucifensin, a Novel Insect Defensin of Medicinal Maggots: Synthesis and Structural Study. ChemBioChem. 2011;12:1352-61.
36. Pöppel A-K, Vogel H, Wiesner J, Vilcinskas A. Antimicrobial Peptides Expressed in Medicinal Maggots of the Blow Fly *Lucilia sericata* Show Combinatorial Activity against Bacteria. Antimicrobial Agents and Chemotherapy. 2015;59(5):2508-14.
37. Mandrioli M, Bugli S, Saltini S, Genedani S, Ottaviani E. Molecular characterization of a defensin in the IZD-MB-0503 cell line derived from immunocytes of the insect *Mamestera brassicae* (Lepidoptera). Biology of the Cell. 2003 Jan-Feb;95(1):53-7.

38. Gao B, Zhu S. Identification and characterization of the parasitic wasp *Nasonia* defensins: Positive selection targeting the functional region? Developmental and Comparative Immunology. 2010 June;34(6):659-68.
39. Ishibashi J, Saido-Sakanaka H, Yang J, Sagisaka A, Yamakawa M. Purification, cDNA cloning and modification of a defensin from the coconut rhinoceros beetle, *Oryctes rhinoceros*. Eur J Biochem. 1999 Dec.;266(2):616-23.
40. Boulanger N, Lowenberger C, Volf P, Ursic R, Sigutova L, Sabatier L, et al. Characterization of a Defensin from the Sand Fly *Phlebotomus duboscqi* Induced by Challenge with Bacteria or the Protozoan Parasite *Leishmania major*. Infection and Immunity. 2004 Dec.;72(12):7140-6.
41. Lambert J, Keppi E, Dimarcq J-L, Wicker C, Reichhart J-M, Dunbar B, et al. Insect immunity: Isolation from immune blood of the dipteran *Phormia terranovae* of two insect antibacterial peptides with sequence homology to rabbit lung macrophage bactericidal peptides. PNAS. 1989;86(262-266).
42. Lamberty M, Zachary D, Lanot R, Bordereau C, Robert A, Hoffmann JA, et al. Constitutive expression of a cysteine-rich antifungal and a linear antibacterial peptide in a termite insect. Journal of Biological Chemistry. 2001 Feb. 9;276(6):4085-92.
43. Cociancich S, Dupont A, Hegy G, Lanot R, Holder F, Hetru C, et al. Novel inducible antibacterial peptides from a hemipteran insect, the sap-sucking bug *Pyrrhocoris apterus*. Biochemical Journal. 1994 June 1;300(Pt. 2):567-75.
44. Matsuyama K, Natori S. Purification of Three Antibacterial Proteins from the Culture Medium of NIH-Sape-4, an Embryonic Cell Line of *Sarcophaga peregrina*. Journal of Biological Chemistry. 1988;263(32):17112-6.
45. Matsuyama K, Natori S. Molecular Cloning of cDNA for Sapecin and Unique Expression of the Sapecin Gene druring Development of *Sarcophaga peregrina*. Journal of Biological Chemistry. 1988;263(32):17117-21.
46. Yamada K, Natori S. Purification, sequence and antibacterial activity of two novel sapecin homologues from *Sarcophaga* embryonic cells: similarity of sapecin B to charybdotoxin. Biochemical Journal. 1993 April 1;291:275-9.
47. Yamada K, Natori S. Characterization of the antimicrobial peptide derived from sapecin B, an antibacterial protein of *Sarcophage peregrina* (flesh fly). Biochemical Journal. 1994;298:623-8.
48. Seufi AM, Hafez EE, Galal FH. Identification, phylogenetic analysis and expression profile of an anionc insect defensin gene, with antibacterial activity, from bacterial-challenged cotton leafworm, *Spodoptera littoralis*. BMC Molecular Biology. 2011;12(47).
49. Lehane MJ, Wu D, Lehane SM. Midgut-specific immune molecules are produced by the blood-sucking insect *Stomoxys calcitrans.* PNAS. 1997 October;94:11502-7.
50. Moon HJ, Lee SY, Kurata S, Natori S, Lee BL. Purification and Molecular Cloning of cDNA for an Inducible Antibacterial Protein from Larvae of the Coleopteran, *Tenebrio molitor* Journal of Biochemistry. 1994 July;116(1):53-8.
51. Lee KH, Hong SY, Oh JE, Kwon My, Yoon JH, Lee Jh, et al. Identification and characterization of the antimicrobial peptide corresponding to C-terminal b-sheet domain of tenecin 1, an antibacterial protein of larvae of *Tenebrio molitor*. Biochemical Journal. 1998;334:99-105.
52. Bulet P, Cociancich S, Dimarcq J-L, Lambert J, Reichhart J-M, Hoffmann D, et al. Isolation from a Coleopteran insect of a novel inducible antibacterial peptide and of new members of the insect defensin family. Journal of Biological Chemistry. 1991 December 25;266(36):24520-5.

53. Rodríguez de la Vega RC, García BI, Ambrosio CD, Diego-García E, Scaloni A, Possani LD. Antimicrobial peptide induction in the haemolymph of the Mexican scorpion *Centruroides limpidus limpidus* in response to septic injury. . Cell Mol Life Sci. 2004 June;61(12):1507-19.
54. Babin DR, Watt DD, Goos SM, Mlejnek RV. Amino acid sequences of neurotoxic protein variants from the venom of Centruroides sculpturatus Ewing. Archives of Biochemistry and Biophysics. 1974;164(2):694-706.
55. Johns R, Sonenshine DE, Hynes WL. Identification of a defensin from the hemolymph of the American dog tick, Dermacentor variabilis. Insect Biochem Molec Biol. 2001 July 26;31(9):857-65.
56. Tsuji N, Battsetseg B, Boldbaatar D, Miyoshi T, Xuan X, Oliver JH, Jr., et al. Babesial Vector Tick Defensin against *Babesia* sp. Parasites. Infection and Immunity. 2007 July;75(7):3633-40.
57. Rahman MM, Tsuji N, Boldbaatar D, Battur B, Liao M, Umemiya-Shirafiju R, et al. Structural characterization and cytolytic activity of a potent antimicrobial motif in longicin, a defensin-like peptide in the tick *Haemophysalis longicornis*. Parasitology. 2010;72(2):149-56.
58. Rudenko N, Golovchenko M, Grubhoffer L. Gene organization of a novel defensin of *Ixodes ricinus*: first annotation of an intron/exon structure in a hard tick defensin gene and first evidence of the occurrence of two isoforms of one member of the arthropod defensin family. Insect Molecular Biology. 2007;16(4):201-507.
59. Chrudimská T, Chrudimský T, Golovchenko M, Rudenko N, Grubhoffer L. New defensins from hard and soft ticks: Similarities, differences, and phylogenetic analyses. Veterinary Parasitology. 2010;167:298-303.
60. Tonk M, Cabezas-Cruz A, Valdés JJ, Rego ROM, Rudenko N, Golovchenko M, et al. Identification and partial characterisation of new members of the *Ixodes ricinius* defensin family. Gene. 2014 May 1;540(2):146-52.
61. Tonk M, Cabezas-Cruz A, Valdés JJ, Rego ROM, Grubhoffer L, Estrada-Peña A, et al. *Ixodes ricinus* defensins attack distantly-related pathogens. Developmental and Comparative Immunology. 2015 Dec.;53(2):358-65.
62. Hynes WL, Ceraul SM, Todd SM, Seguin KC, Sonenshine DE. A defensin-like gene expressed in the black-legged tick, *Ixodes scapularis*. Medical and Veterinary Entomology. 2005 Dec.;19(4):339-44.
63. Tonk M, Cabezas-Cruz A, Valdés JJ, Rego ROM, Chrudimská T, Strnad M, et al. Defensins from the tick *Ixodes scapularis* are effective against phytopathogenic fungi and the human bacterial pathogen *Listeria grayi*. Parasites & Vectors. 2014;7:554.
64. Wang Y, Zhu S. The defensin gene family expansion in the tick *Ixodes scapularis*. Developmental and Comparative Immunology. 2011;35:1128-34.
65. Miller C, Moczydlowski E, Latorre R, Phillips M. Charybdotoxin, a protein inhibitor of single Ca^{2+}-activated K^+ channels from mammalian skeletal muscle. Nature. 1985;313:316-8.
66. Gimenez-Gallego G, Navia MA, Reuben JP, Katz GM, Kaczorowski GJ, Garcia ML. Purification, sequence, and model structure of charybdotoxin, a potent selective inhibitor of calcium-activated potassium channels. Proceedings of the National Academy of Sciences, USA. 1988;85:3329-33.
67. Yount NY, Yeaman MR. Multidimensional signatures in antimicrobial peptides. Proc Natl Acad Sci USA. 2004;101(19):7363-8.

68. Cociancich S, Goyffon M, Bontems F, Bulet P, Bouet F, Menex A, et al. Purification and characterization of a scorpion defensin, a 4kDa antibacterial peptide presenting structural similarities with insect defensins and scorpion toxins. Biochemical an Biophysical Research Communications. 1993;194(1):17-22.
69. Romi-Lebrun R, Lebrun B, Martin-Eauclaire M-F, Ishiguro M, Escoubas P, Wu FQ, et al. Purification, Characterization, and Synthesis of Three Novel Toxins from the Chinese Scorpion *Buthus martensi*, which Act on K+ Channels. Biochemistry. 1997 Nov.;36:13473-82.
70. Zhou H, Kong Y, Wang H, Tianhua Y, Feng F, Bian J, et al. A defensin-like antimicrobial peptide from the venoms of spider, *Ornithoctonus hainana*. Journal of Peptide Science. 2011 April 2011;17:540-4.
71. Nakajima Y, van der Goes van Naters-Yasui A, Taylor D, Yamakawa M. Two isoforms of a member of the arthropod defensin family from the soft tick, *Ornithodoros moubata* (Acari: Argasidae). Insect Biochem Molec Biol. 2001 June 22;31(8):747-51.
72. Nakajima Y, Ishibashi J, Yukuhiro F, Asaoka A, Taylor D, Yamakawa M. Antibacterial activity and mechanism of action of tick defensin against Gram-positive bacteria. Biochimica et Biophysica Acta. 2003;1624:125-30.
73. Conde R, Zamudio FZ, Rodríguez MH, Possani LD. Scorpine, an anti-malaria and anti-bacterial agent purified from scorpion venom. FEBS Letters. 2000 April 14;471(2-3):165-8.
74. Zhang C, He X, Gu Y, Zhou H, Cao J, Gao Q. Recombinant scorpine produced using SUMO fusion partner in *Escherichia coli* has the activities against clinicially isolated bacteria and inhibits the *Plasmodium falciparum* parasitemia *in vitro*. PloS ONE. 2014;9(7):e103456.
75. Kato Y, Komasku S. ASABF, a novel cysteine-rich antibacterial peptide isolated from the nematode *Ascaris suum*. J Biol Chem. 1996 November 29, 1996;271(48):30493-8.
76. Kato Y, Aizawa T, Hoshino H, Kawano K, Nitta K, Zhang H. *abf-1* and *abf-2*, ASABF-type antimicrobial peptide genes in *Caenorhabditis elegans*. Biochem J. 2002 Jan. 15;361(Pt2):221-30.
77. Zhu S, Gao B. Nematode-derived drosomycin-type antifungal peptdies provide evidence for plant-to-ecdysozoan horizontal transfer of a disease resistance gene. Nature Communications. 2014;5.
78. Zhong J, Wang W, Yang X, Yan X, Liu R. A novel cysteine-rich antimicroial peptide from the mucus of the snail of *Achatina fulica*. Peptides. 2013;39:1-5.
79. Gueguen Y, Herpin A, Aumelas A, Garnier J, Fievet J, Escoubas J-M, et al. Characterization of a Defensin from the Oyster Crassostrea gigas: recombinant production, folding solution structure, antimicrobial activities, and gene expression. Journal of Biological Chemistry. 2006 January 6;281:313-23.
80. Seo J-K, Crawford JM, Stonge KL, Noga EJ. Purification of a novel arthropod defensin from the American oyster, *Crassostrea virginica*. Biochemical an Biophysical Research Communications. 2005 Dec. 30;338(4):1998-2004.
81. Xu W, Faisal M. Defensin of the zebra mussel (*Dreissena polymorpha*): Molecular structure, *in vitro* expression, antimicrobial activity, and potential functions. Mol Immunol. 2010 July;47(11-12):2138-47.
82. Charlet M, Chernysh S, Philippe H, Hetru C, Hoffmann JA, Bulet P. Innate immunity. Isolation of several cyteine-rich antimicrobial peptides from the blood of a mollusc, *Mytilus edulis.* J Biol Chem. 1996;271(36):21808-13.
83. Hubert F, Noël T, Roch P. A member of the arthropod defensin family from edible Mediterranean mussels (*Mytilus galloprovincialis)*. Eur J Biochem. 1996 Aug. 15;240(1):302-6.

84. Mitta G, Vandenbulcke F, Hubert F, Roch P. Mussel defensins are synthesised and processed in granulocytes then released into the plasma after bacterial challenge. J Cell Sci. 1999;112:4233-42.
85. Yang Y-S, Mitta G, Chavanieu A, Calas B, Sanchez JF, Roch P, et al. Solution structure and activity of the synthetic four-disulfide bond Mediterranean mussel defensin (MGD-1). Biochemistry. 2000;39:14436-47.
86. Romestand B, Molina F, Richard V, Roch P, Granier C. Key role of the loop connecting the two beta strands of mussel defensin in its antimicrobial activity. Eur J Biochem. 2003;270:2805-13.
87. Roch P, Beschin A, Bernard E. Antiprotozoan and antiviral activities of non-cytotoxic truncated and variant analogues of mussel defensin. Evidence-based Complementary and Alternative Medicine. 2004 August;1(2):167-74.
88. Mitta G, Hubert F, Noël T, Roch P. Myticin, a novel cystein-rich antimicrobial peptide isolated from haemocytes and plasma of the mussel *Mytilus galloprovincialis.* Eur J Biochem. 1999 Oct. 1;265(1):71-8.
89. Mitta G, Vandenbulcke F, Hubert F, Salzet M, Roch P. Involvement of Mytilins in Mussel Antimicrobial Defense. Journal of Biological Chemistry. 2000 April 28;275(17):12954-62.
90. Mitta G, Hubert F, Dyrynda EA, Boudry P, Roch P. Mytilin B and MGD2, two antimicrobial peptides of marine mussels: gene structure and expression analysis. Developmental and Comparative Immunology. 2000 June;24(4):381-93.
91. Tasiemski A, Vandenbulcke F, Mitta G, Lemoine J, Lefebvre C, Sautière P-E, et al. Molecular Characterization of Two Novel Antibacterial Peptdies Inducivle upon Bacterial Challenge in an Annelid, the Leech *Theromyzon tessulatum*. Journal of Biological Chemistry. 2004 April 21;279(30):30973-82.
92. Osborn RW, De Samblanx GW, Thevissen K, Goderis I, Torrekens S, Van Leuven F, et al. Isolation and characterisation of plant defensins from seeds of Asteraceae, Fabaceae, Hippocastanaceae, and Saxigragaceae. FEBS Letters. 1995 July 17;368(2):257-62.
93. Mirouze M, Sels J, Richard O, Czernic P, Loubet S, Jacquier A, et al. A putative novel role for plant defensins: a defensin from the zinc hyper-accumulating plant, Arabidopsis halleri, confers zinc tolerance. The Plant Journal. 2006 August;47(3):329-42.
94. Marquès L, Oomen RJFJ, Aumelas A, Le Jean M, Berthomieu P. Production of an *Arabidopsis halleri* foliar defensin in *Escherichia coli*. Journal of Applied Microbiology. 2009;106:1640-8.
95. Terras FRG, Torrekens S, Van Leuven F, Osborn RW, Vanderleyden J, Cammue BPA, et al. A new family of basic cysteine-rich plant antifungal proteins from Brassicaceae species. FEBS. 1993 Feb.;3:233-40.
96. Sels J, Delauré SL, Aerts AM, Proost P, Cammue BPA, De Bolle MFC. Use of PTGS-MAR expression system for efficient in planta production of bioactive *Arabidopsis thaliana* plant defensins. Transgenic Research. 2007 August;16(4):531-8.
97. Penninckx IAMA, Eggermont K, Terras FRG, Thomma BPHJ, De Samblanx GW, Buchala A, et al. Pathogen-Induced Systemic Activation of a Plant Defensin Gene in Arabidopsis Follows a Salicylic Acid-Independent Pathway. The Plant Cell. 1996;8:2309-23.
98. Kragh KM, Nielsen JE, Nielsen KK, Dreboldt S, Mikkelsen JD. Characterizaton and Localization of New Antifungal Cysteine-Rich Proteins from *Beta vulgaris*. Molecular Plant-Microbe Interactions. 1995 May-June;8(3):424-34.
99. Park HC, Kang YH, Chun HJ, Koo JC, Cheong YH, Kim CY, et al. Characterization of a stamen-specific cDNA encoding a novel plant defensin in Chinese cabbage. Plant Molecular Biology. 2002 Sept.;50(1):59-69.

100. Meyer B, Houlné G, Pozueta-Romero J, Schantz M-L, Schantz R. Fruit-specific expression of a defensin-type gene family in bell pepper. Plant Physiology. 1996;112:615-22.
101. Li D, Li J. Antifungal activity of a recombinant defensin CADEF1 produced by *Escherichia coli*. World J Microbiol Biotechnol. 2009 November;25(11):1911-8.
102. Anaya-López JL, López-Meza JE, Baizabal-Aguirre VM, Cano-Camacho H, Ochoa-Zarzosa A. Fungicidal and cytotoxic activity of a *Capsicum chinense* defensin expressed by endothelial cells. Biotechnol Lett. 2006 July;28(14):1101-8.
103. Odintsova TI, Rogozhin EA, Baranov Y, Musolyamov AK, Yalpani N, Egorov TA, et al. Seed defensins of barnyard grass *Echinochloa crusgalli* (L.) Beauv. Biochimie. 2008 Nov-Dec;90(11-12):1667-73.
104. Harrison SJ, Marcus JP, Goulter KC, Green JL, Maclean DJ, Manners JM. An Antimicrobial Peptide from the Australian Native *Hardenbergia violacea* Provides the First Functionally Characterised Member of a Subfamily of Plant Defensins. Australian Journal of Plant Physiology. 1997;24(5):571-8.
105. de Zélicourt A, Letousey P, Thoiron S, Campion C, Simoneau P, Elmorjani K, et al. Ha-DEF1, a sunflower defensin, induces cell death in *Orobanche* parasitic plants. Planta. 2007 August;226(3):591-600.
106. Huang G-J, Lai H-C, Chang Y-S, Sheu M-J, Lu T-L, Huang S-S, et al. Antimicrobial, Dehydroascorbate Reductase, and Monodehydroascorbate Reductase Activities of Defensin from Sweet Potato [*Ipomoea batatas* (L.) Lam. 'Tainong 57'] Storage Roots. Journal of Agricultural and Food Chemistry. 2008 May 14;56(9):2989-95.
107. Finkina EI, Shramova EI, Tagaev AA, Ovchinnikova TV. A novel defensin from the lentil *Lens culinaris* seeds. Biochemical an Biophysical Research Communications. 2008 July 11;371(4):860-5.
108. Solis J, Medrano G, Ghislain M. Inhibitory effect of a defensin gene from the Andean crop maca (*Lepidium meyenii*) against *Phytophthora infestans*. Journal of Plant Physiology. 2007 August;164(8):1071-82.
109. Gao A-G, Hakimi SM, Mittanck CA, Wu Y, Woerner BM, Stark DM, et al. Fungal pathogen protection in potato by expression of a plant defensin peptide. Nature Biotechnology. 2000 December;18:1307-10.
110. Spelbrink RG, Dilmac N, Allen A, Smith TJ, Shah DM, Hockerman GH. Differential Antifungal and Calcium Channel-Blocking Activity among Structurally Related Plant Defensins. Plant Physiology. 2004 August;135:2055-67.
111. Allen A, Snyder AK, Preuss M, Nielsen EE, Shah DM, Smith TJ. Plant defensins and virally encoded fungal toxin KP4 inhibit plant root growth. Planta. 2008 Jan;227(2):331-9.
112. Lay FT, Brugliera F, Anderson MA. Isolation and Properties of Floral Defensins from Ornamental Tobacco and Petunia. Plant Physiology. 2003 March;131:1283-93.
113. Portieles R, Ayra C, Gonzalez E, Gallo A, Rodriguez R, Chacón O, et al. *NmDef02*, a novel antimicrobial gene isolated from *Nicotiana megalosiphon* confers high-level pathogen resistance under greenhouse and field conditions. Plant Biotechnology Journal. 2010 August;8(6):678-90.
114. Rogozhin EA, Oshchepkova YI, Odintsova TI, Khadeeva NV, Veshkurova ON, Egorov TA, et al. Novel antifungal defensin from *Nigella sativa* L. seeds. Plant Physiology and Biochemistry. 2011 Feb.;49(2):131-7.
115. Song X, Zhou Z, Wang J, Wu F, Gong W. Purification, characterization and preliminary crystallographic studies of a novel plant defensin from *Pachyrrhizus erosus* seeds. Acta Crystallographica 2004 June;D60(Pt 6):1121-4.

116. Song X, Wang J, Wu F, Li X, Teng M, Gong W. cDNA cloning, functional expression and antifungal activities of a dimeric plant defensin SPE10 from *Pachyrrhizus erosus* seeds. Plant Molecular Biology. 2005;57:13-20.
117. Ming D, Hellekant G. Brazzein, a new high-potency thermostable sweet protein from *Pentadiplandra brazzeana* B. FEBS Letters. 1994;355:106-8.
118. Wang HX, Ng TB. An antifungal peptide from baby lima bean. Applied Microbiology and Biotechnology. 2006 Dec.;73(3):576-81.
119. Wong JH, Zhang XQ, Wang HX, Ng TB. A mitogenic defensin from white cloud beans (*Phaseolus vulgaris*). Peptides. 2006 Sept.;27(9):2075-81.
120. Games PD, dos Santos IS, Mello ÉO, Diz MSS, Carvalho AO, de Souza-Filho GA, et al. Isolation, characterization and cloning of a cDNA encoding a new antifungal defensin from *Phaseolus vulgaris* L. seeds. Peptides. 2008 Dec.;29(12):2090-100.
121. Kovaleva V, Kiyamova R, Cramer R, Krynytskyy H, Gout I, Filonenko V, et al. Purification and molecular cloning of antimicrobial peptides from Scots pine seedlings. Peptides. 2009 Dec.;30(12):2136-43.
122. Kovaleva V, Krynytskyy H, Gout I, Gout R. Recombinant expression, affinity purification and functional characterization of Scots pine defensin 1. Applied Microbiology and Biotechnology. 2011;89:1093-101.
123. Almeida MS, Cabral KMS, Zingali RB, Kurtenbach E. Characterization of Two Novel Defense Peptides from Pea *(Pisum sativum)* Seeds. Archives of Biochemistry and Biophysics. 2000 June 15;378(2):278-86.
124. Wisniewski ME, Bassett CL, Artlip TS, Webb RP, Janisiewicz WJ, Norelli JL, et al. Characterizaton of a defensins in bark and fruit tissues of peach and antimicrobial activity of a recombinant defensin in the yeast, *Pichia pastoris*. Physiologia Plantarum. 2003 Dec.;119(4):563-72.
125. Terras FRG, Schoofs HME, De Bolle MFC, van Leuven F, Rees SB, Vanderleyden J, et al. Analysis of Two Novel Classes of Plant Antifungal Proteins from Radish (*Raphanus sativus* L.) Seeds. Journal of Biological Chemistry. 1992;267(22):15301-9.
126. De-Paula VS, Razzera G, Medeiros L, Miyamoto CA, Almeida MS, Kurtenbach E, et al. Evolutionary relationship between defensins in the Poaceae family strengthened by the characterization of new sugarcane defensins. Plant Molecular Biology. 2008 Nov.;68(4-5):321-35.
127. Stotz HU, Spence B, Wang Y. A defensin from tomato with dual function in defense and development. Plant Molecular Biology. 2009 Sept.;71(1-2):131-43.
128. Segura A, Moreno M, Molina A, García-Olmedo F. Novel defensin subfamily from spinach (*Spinacia oleracea*). FEBS Letters. 1998 Sept. 18;435(2-3):159-62.
129. Slavokhotova AA, Odintsova TI, Rogozhin EA, Musolyamov AK, Andreev YA, Grishin EV, et al. Isolation, molecular cloning and antimicrobial activity of novel defensins from common chickweed (*Stellaria media* L.) seeds. Biochimie. 2011 March;93(3):450-6.
130. Vijayan S, Guruprasad L, Kirti PB. Prokaryotic expression of a constitutively expresssed *Tephrosia villosa* defensin and its potent antifungal activity. Applied Microbiology and Biotechnology. 2008 October;80(6):1023-32.
131. Da-Hui L, Gui-Liang J, Ying-Tao Z, Tie-Min A. Bacterial expression of a *Trichosanthes kirilowii* defensin (TDEF1) and its antufungal activity on *Fusarium oxysporum*. Applied Microbiology and Biotechnology. 2007 Feb.;74(1):146-51.
132. Olli S, Kirti PB. Cloning, Characterization and Antifungal Activity of Defensin Tfgd1 from *Trigonella foenum-graecum* L. Journal of Biochemistry and Molecular Biology. 2006;39(3):278-83.

133. Koike M, Okamoto T, Tsuda S, Imai R. A novel plant defensin-like gene of winter wheat is specifically induced during cold acclimation. Biochemical an Biophysical Research Communications. 2002 Oct. 18;298(1):46-53.
134. Chen G-H, Hsu M-P, Tan C-H, Sung H-Y, Kuo CG, Fan M-J, et al. Cloning and Characterization of a Plant Defensin VaD1 from Azuki Bean. Journal of Agricultural and Food Chemistry. 2005 Feb. 23;53(4):982-8.
135. Chen K-C, Lin C-Y, Kuan C-C, Sung H-Y, Chen C-S. A Novel Defensin Encoded by a Mungbean cDNA Exhibits Insecticida Activity against Bruchid. Journal of Agricultural and Food Chemistry. 2002 Dec. 4;50(25):7258-63.
136. Chen J-J, Chen G-H, Hsu H-C, Li S-S, Chen C-S. Cloning and Functional Expression of a Mungbean Defensin VrD1 in *Pichia pastoris*. Journal of Agricultural and Food Chemistry. 2004 April 21;52(8):2256-61.
137. Franco OL, Murad AM, Leite JR, Mendes PAM, Prates MV, Bloch C, Jr. Identification of a cowpea g-thionin with bactericidal activity. FEBS Journal. 2006 August;273(15):3489-97.
138. de Beer A, Vivier MA. Vv-AMPI, a ripening induced peptide from *Vitis vinifera* shows strong antifungal activity. BMC Plant Biology. 2008 July 8;8:75.
139. Saitoh H, Kiba A, Nishihara M, Yamamura S, Suzuki K, Terauchi R. Production of Antimicrobial Defensin in *Nicotiana benthamiana* with a Potato Virus X Vector. MPMI. 2001 Feb.;14(2):111-5.
140. Kushmerick C, de Souza Castro M, Cruz JS, Bloch C, Jr., Beirão PSL. Functional and structural features of g-zeathionins, a new class of sodium channel blockers. FEBS Letters. 1998 Dec. 4;440(3):302-6.
141. Kant P, Liu W-Z, Pauls KP. PDC1, a corn defensin peptide expressed in *Escherichia coli* and *Pichia pastoris* inhibits the growth of *Fusarium graminearum*. Peptides. 2009 Sept.;30(9):1593-9.
142. Balandin M, Royo J, Gómez E, Muniz LM, Molina A, Hueros G. A protective role for the embryo surrounding region of the maize endosperm, as evidened by the characterisation of *ZmESR-6,* a defensin gene specifically expressed in this region. Plant Molecular Biology. 2005 May;58(2):269-82.
143. Zhu S, Gao B, Harvey PJ, Craik DJ. Dermatophytic defensin with antiinfective potential. PNAS. 2012;109(22):8495-500.
144. Mygind PH, Fischer RL, Schnorr KM, Hansen MT, Sönksen CP, Ludvigsen S, et al. Plectasin is a peptide antibiotic with therapeutic potential from a saprophytic fungus. Nature. 2005 October;437:975-80.
145. Simon A, Kullberg BJ, Tripet B, Boerman OC, Zeeuwen P, van der Ven-Jongekrijg J, et al. Drosomycin-Like Defensin, a Human Homologue of *Drosophila melanogaster* Drosomycin with Antifungal Activity. Antimicrobial Agents and Chemotherapy. 2008 April;52(4):1407-12.
