# Supplementary material for: Establishing a reference array for the CS-αβ superfamily of defensive peptides
Source: BMC Res Notes. 2016 Nov 18;9:490. doi: 10.1186/s13104-016-2291-0 (PMC5116183; doi:10.1186/s13104-016-2291-0)
Supplement: Supplementary file 3 — Additional file 3: Table S2. Representative CS-αβ sequences used for phylogenetic analysis. Accession numbers are shown for each sequence used in the analysis. The “Tree Label” column corresponds to the names shown in Fig. 2 and 3. The “Sequences Represented” column shows information for sequences excluded from the analysis based on similarity to the representative sequence. In cases where a sequence is represented by more than one accession number, the sequence used is shown in bold. [file 13104_2016_2291_MOESM3_ESM.pdf]

Additional File 3: Table S2. Representative CS-αβ sequences used for phylogenetic analysis. Accession numbers are shown for each sequence used in the analysis. The “Tree Label” column corresponds to the names shown in Figure 2. The “Sequences Represented” column shows information for sequences excluded from the analysis based on similarity to the representative sequence. In cases where a sequence is represented by more than one accession number, the sequence used is shown in bold.

**Species (by major taxonomic group) and name of peptide (if one has been given)**	**Accession number**	**Tree Label**	**Sequences Represented**
**Bacteria**			
Anaeromyxobacter dehalogenans AdDLP	[NCBI Reference Sequence: WP_011422871]	AdDLP	
**Cnidaria**			
Acropora palmata	[GenBank: **GW203080,** GW203081, GW208862, GW208863]	Acropora	
Anemonia viridis	[GenBank: **FK731853**, FK754575, FK756642, FK751627, FK747619, FK737168]	Anemonia	*Anemonia viridis* [GenBank: FK740859]
Clytia hemisphaerica	[GenBank: **FP967549**, FK985645]	Clytia	*Clytia hemisphaerica* [GenBank: FP943555, FP966428]
Hydra magnipapillata Hydramacin	[GenBank: ABE26989]	Hydramacin	
Metridium senile	[GenBank: **FC831074**, FC832453, FC840147, FC826793, FC834614, FC826372, FC837794]	Metridium	
Montastraea feveolata	[GenBank: GW256715]	Montastraea	*Montastraea faveolata* [GenBank: GW270650, GW270651]
Podocoryna carnea	[GenBank: DY451332]	Podocoryna	
**Porifera**			
Leucetta chagosensis	[GenBank: GO092849]	Leucetta GO092849	
Leucetta chagosensis	[GenBank: GO091294]	Leucetta GO091294	
Subiteres domuncula ASABF-related peptide	[GenBank: CCC55928]	ASABF-RP	
**Hexapoda**			
Acalolepta luxuriosa AlCRP	[GenBank: AB104817]	AlCRP	

Aedes aegypti Defensin A	[Swiss-Prot: P91793]	Aedes DefA	*Aedes aegypti* Defensins B, C [Swiss-Prot: P81602, P81603]; *Anopheles gambiae* Defensin [Swiss-Prot: Q17027]
Aeschna cyanea Defensin	[Swiss-Prot: P80154]	Aeschna	
Allomyrina dichotoma (Trypoxylus dichotomus) Defensin	[Swiss-Prot: Q10745]	Allomyrina	*Anomala cuprea* Defensins A, B [Swiss-Prot: P83669, P83668]; *Oryctes rhinoceros* Defensin [Swiss-Prot: O96049]
Apis mellifera Royalisin	[Swiss-Prot: P17722]	Royalisin	*Bombus pascuorum* Defensin [Swiss-Prot: P81462]
Bombyx mori Defensin A	[NCBI Reference Sequence: NP_001037370]	Bombyx DefA	*Bombyx mori* Defensin-like protein [Swiss-Prot: Q45RF8]; *Spodoptera frugiperda* Spodoptericin [GenBank: AAQ18895]; *Spodoptera littoralis* SpliDef [GenBank: HQ603825]
Bombyx mori Defensin B	[GenBank: BAG71131]	Bombyx DefB	
Chironomus plumosus Defensin A	[1]	Chironomus DefA	*Chironomus plumosus* Defensin B [1]
Copris tripartitus Coprisin	[GenBank: ABP97087]	Coprisin	
Drosophila melanogaster Defensin	[Swiss-Prot: P36192]	Drosophila	
Drosophila melanogaster Drosomycin	[Swiss-Prot: P41964]	Drosomycin	*Drosophila melanogaster* Drosomycin 2 [GenBank: ABY84135]
Eristalis tenax Eristalin	[GenBank: AM706420]	Eristalin	

Galleria mellonella Gallerimycin	[Swiss-Prot: Q8MVY9]	Gallerimycin	*Samia cynthia ricini* Scr-gallerimycin [GenBank: BAG12297]; *Spodoptera frugiperda* Gallerimycin [Swiss-Prot: Q6XD81]; *Spodoptera litura* Sl-gallerimycin [GenBank: JG406586]
Galleria mellonella Defensin	[Swiss-Prot: P85213]	Galleria	*Archeoprepona demophon* ARD1 [Swiss-Prot: P84156]; *Heliothis virescens* Heliomicin [GenBank: ACR78445]
Glossina morsitans Defensin A	[Swiss-Prot: Q8WTD4]	Glossina	
Holotrichia diomphalia Holotricin 1	[Swiss-Prot: Q7M426]	Holotricin 1	
Lucilia sericata Lucifensin 2	[GenBank: ADI87383]	Lucifensin 2	*Lucilia sericata* Lucifensin 3 [2]
Lucilia sericata Lucifensin 1	[2]	Lucifensin 1	
Lucilia sericata Lucifensin 6	[2]	Lucifensin 6	
Lucilia sericata Lucifensin 7	[2]	Lucifensin 7	
Mamestra brassicae Defensin	[GenBank: AAL69980]	Mamestra	
Nasonia vitripennis Navidefensin2-2	[3]	Navidefensin2-2	*Apis mellifera* Defensin 2 [NCBI Reference Sequence: NP_001011638]
Phlebotomus duboscqi Defensin	[Swiss-Prot: P83404]	Phlebotomus	
Protophormia terraenovae Phormicin A	[Swiss-Prot: P10891]	Phormicin A	*Protophormia terraenovae* Phormicin B (G86R) [Swiss-Prot: P10891]
Pseudacanthotermes spiniger Termicin	[Swiss-Prot: P82321]	Termicin	*Macrotermes barneyi* Termicin [GenBank: ACO90349]

Pyrrhocoris apterus Defensin 1	[Swiss-Prot: P37364]	Pyrrhocoris Def1	*Pyrrhocoris apterus* Defensin 2 [GenBank: AGI17576]; *Rhodnius prolixus* Defensins A, B, C [GenBank: AAO74624, AAO74625, AAO74626]; *Triatoma brasiliensis* Defensin 1 [Swiss-Prot: Q4VSI0]
Sarcophaga peregrina Sapecin A	[Swiss-Prot: P18313]	Sapecin A	*Sarcophaga peregrina* Sapecin C [Swiss-Prot: P31530]
Sarcophaga peregrina Sapecin B	[Swiss-Prot: P31529]	Sapecin B	*Lucilia sericata* Lucifensin 4 [2]
Spodoptera frugiperda Sf-cobatoxin	[GenBank: AAP69839]	Sf-cobatoxin	
Stomoxys calcitrans Smd1	[Swiss-Prot: O16136]	Smd1	
Stomoxys calcitrans Smd2	[Swiss-Prot: O16137]	Smd2	*Formica aquilonia* Defensin [GenBank: AAX20157]; *Formica rufa* Defensin [4]
Tenebrio molitor Tenecin 1	[Swiss-Prot: Q27023]	Tenecin 1	*Acalolepta luxuriosa* Defensin 1 [Swiss-Prot: Q9BK52]; *Zophobas atratus* Peptides B, C [GenBank: AAB20745, AAB20746]
**Arachnida**			
Androctonus australis hector Toxin II	[PDB: 1AHO]	Androctonus toxin II	
Androctonus mauritanicus mauritanicus Kaliotoxin	[PDB: 2KTX]	Kaliotoxin	*Leiurus quinquestriatus hebraeus* Agitoxin [PDB: 1AGT]; *Mesobuthus martensii (Buthus)* Bmktx [PDB: 1BKT]; *Orthochirus scrobiculosus* Osk1 toxin [PDB: 1SCO]
Androctonus mauritanicus mauritanicus P01	[PDB: 1ACW]	Androctonus P01	*Mesobuthus martensii (Buthus)* Bmp01 [PDB:

			1WM7]
Buthus occitanus tunetanus LVP1a	[Swiss-Prot: P84810]	LVP1a	*Buthus occitanus tunetanus* LVP1b [Swiss-Prot: P84809]
Centruroides exilicauda Neurotoxin	[PDB: 1NH5]	Neurotoxin 1NH5	
Centruroides limpidus Cll-dlp	[Swiss-Prot: Q6GU94]	Cll-dlp	
Centruroides margaritatus Margatoxin	[PDB: 1MTX]	Margatoxin	*Centruiroides limbatus* Hongotoxin [PDB: 1HLY]; *Centruroides noxius* Noxiustoxin [PDB: 1SXM]
Centruroides noxius Cobatoxin	[PDB: 1PJV]	Cobatoxin	
Centruroides noxius Ergtoxin	[PDB: 1PX9]	Ergtoxin	
Centruroides sculpturatus CsEv2 neurotoxin	[Swiss-Prot: P01493]	CsEv2	*Centruroides exilicauda* Scorpion toxin variant 3 [PDB: 2SN3]; *Centruroides noxius* Toxin 2 [PDB: 1CN2] and Toxin Cn12 [PDB: 1PE4]; *Centruroides sculpturatus* Variant 1 neurotoxin, beta-neurotoxin [PDB: 1VNA, 2B3C]
Centruroides sculpturatus Neurotoxin	[PDB: 1NRA]	Neurotoxin 1NRA	
Haemaphysalis longicornis Longicin	[Swiss-Prot: Q58A47]	Longicin	*Dermacentor andersoni* Defensin [GenBank: ABK62866]; *D. marginatus* Defensin [GenBank: ACJ04433]; *D. variabilis* Varisin A1 [Swiss-Prot: Q86QI5]; *Ixodes ricinus* Defensin MT6 [GenBank: JAA71516]; *I. scapularis* Scapularisin 1 [GenBank: EEC08934]
Heterometrus spinifer Hstx1	[PDB: 1QUZ]	Hstx1	
Hottentotta judaicus (Buthotus judaicus) Toxin Bjxtr-It	[PDB: 1BCG]	Toxin Bjxtr-It	

Ixodes ricinus Defensin 2	[GenBank: ABC88432]	Iricinus Def2	*Ixodes ricinus* Defensins 1, MT3, MT4 [GenBank: AAP94724, JAA71488, JAA71477]; *Ixodes scapularis* Scapularisins 19, 20, 22, 23, 24 [GenBank: EEC01374, EEC17844, EEC03289, ABJB010061346, ABJB010520452]
Ixodes ricinus Defensin MT2	[GenBank: JAA65352]	Iricinus DefMT2	*Ixodes ricinus* Defensin MT5 [GenBank: JAA66832]; *I. scapularis* Scapularisin 15 [GenBank: ABJB010719289]
Ixodes ricinus Defensin MT7	[GenBank: JAA69779]	Iricinus DefMT7	*Ixodes scapularis* Scapularisin 16 [GenBank: EEC17916]
Ixodes scapularis Scapularisin 3	[GenBank: EEC13914]	Scapularisin 3	*Ixodes scapularis* Scapularisin 2 [GenBank: ABJB010277423]
Ixodes scapularis Scapularisin 4	[GenBank: ABJB010377501]	Scapularisin 4	
Ixodes scapularis Scapularisin 6	[GenBank: AAV74387]	Scapularisin 6	*Ixodes scapularis* Scapularisins 5, 7-11 [GenBank: EEC08933, ABJB011101692, ABJB011096562, ABJB011065840, ABJB010379933, ABJB011080676]
Ixodes scapularis Scapularisin 25	[GenBank: ABJB010066055]	Scapularisin 25	
Ixodes scapularis Scasin 1	[GenBank: EEC18782]	Scasin 1	*Ixodes scapularis* Scasin 2 [GenBank: ABJB010977893]
Ixodes scapularis Scasin 4	[GenBank: ABJB010586641]		*Ixodes scapularis* Scasin 5 [GenBank: ABJB010739942]

Ixodes scapularis Scasin 7	[GenBank: ABJB010767832]	Scasin 7	
Ixodes scapularis Scasin 8	[GenBank: ABJB010495451]	Scasin 8	
Ixodes scapularis Scasin 9	[GenBank: ABJB010461586]	Scasin 9	
Ixodes scapularis Scasin 10	[GenBank: ABJB010760265]	Scasin 10	*Ixodes scapularis* Scasin 11 [GenBank: ABJB010086782]
Ixodes scapularis Scasin 13	[GenBank: ABJB010272194]	Scasin 13	*Ixodes scapularis* Scasin 14 [GenBank: ABJB010907808]
Ixodes scapularis Scasin 16	[GenBank: ABJB011065775]	Scasin 16	
Ixodes scapularis Scasin 18	[GenBank: ABJB010836816]	Scasin 18	*Ixodes scapularis* Scasins 17, 19, 21 [GenBank: ABJB010453558, ABJB011050833, ABJB010067897]
Leiurus quinquestriatus Chlorotoxin	[PDB: 1CHL]	Chlorotoxin	*Mesobuthus eupeus* Insectotoxin [PDB: 1SIS]
Leiurus quinquestriatus hebraeus Defensin	[Swiss-Prot: P41965]	Leiurus Defensin	
Leiurus quinquestriatus hebraeus Charybdotoxin	[Swiss-Prot: P13487]	Charybdotoxin	*Leiurus quinquestriatus hebraeus* Lq2 [PDB: 1LIR];<br>*Mesobuthus martensii (Buthus)* BmTx2 [PDB: 2BMT]
Leiurus quinquestriatus hebraeus Insecticidal alpha scorpion toxin	[PDB: 1LQI]	Leiurus 1LQI	*Leiurus quinquestriatus quinquestriatus* Anti-mammal and anti-insect LQQii toxin<br>[PDB: ILQQ]
Leiurus quinquestriatus hebraeus Alpha-like toxin Lgh Iii	[PDB: 1BMR]	Leiurus 1BMR	
Leiurus quinquestriatus hebraeus Scyllatoxin	[PDB: 1SCY]	Scyllatoxin	*Androctonus mauritanicus mauritanicus* Po-Nh2 [PDB: 1PNH]
Mesobuthus eupeus Herg-specific scorpion toxin Bekm-1	[PDB: 1LGL]	Bekm-1	
*Mesobuthus martensii (Buthus)*K-channel toxin	[Swiss-Prot: Q9NII6]	Mesobuthus K-toxin	

Mesobuthus martensii (Buthus) Neurotoxin Bmk M1	[PDB: 1SN1]	Bmk M1	*Mesobuthus martensii (Buthus)* Neurotoxin and Alpha-like toxin Bmk M7 [PDB: 1CHZ, KV0]
Mesobuthus martensii (Buthus) Neurotoxin Bmk M4	[PDB: 1SN4]	Bmk M4	
Mesobuthus martensii (Buthus) Neurotoxin Bmk M8	[PDB: 1SNB]	Bmk M8	
Mesobuthus martensii (Buthus) Alpha-insect toxin Bmkait 1	[PDB: 1OMY]	Bmkait1	
Mesobuthus martensii (Buthus) Bmbktx1	[PDB: 1Q2K]	Bmbktx1	
Mesobuthus martensii (Buthus) BmTx3	[PDB: 1M2S]	BmTx3	
Mesobuthus martensii (Buthus) Bmp02	[PDB: 1DU9]	Bmp02	*Mesobuthus martensii (Buthus)* Bmp03 [PDB: 1WM8]
Mesobuthus martensii (Buthus) Bmp07	[PDB: 1PVZ]	Bmp07	
Mesobuthus martensii (Buthus) Mesotoxin	[Swiss-Prot: A0F0C2]	Mesotoxin	
Mesobuthus tamulus Neurotoxin	[PDB: 1DQ7]	Mt Neurotoxin	
Ornithoctonus hainana Defensin	Sequence from [5]	Ornithoctonus	
Ornithodoros moubata Defensin A	[Swiss-Prot: Q9BLJ3]	Omoubata DefA	*Ornithodoros moubata* Defensins B, C, D [GenBank: BAB41027, BAC22074, BAC22073]; *O. papillipes* Defensins A, B, D [GenBank: ACJ04425, ACJ04426, ACJ04427]; *O. puertoricensis* Defensins A, B [6]; *O. rostratus* Defensin A [GenBank: ACJ04428]; *O. tartakovskyi* Defensins A, B [GenBank: ACJ04431; ACJ04432]
Pandinus imperator Scorpine	[Swiss-Prot: P56972]	Scorpine	
Pandinus imperator Toxin K-A (Pitx-Ka)	[PDB: 2PTA]	Pitx-Ka	*Pandinus imperator* Toxin K-beta [PDB: 1C49]

Phoneutria reidyi Defensin	[7]	Phoneutria	*Argiope* sp. Defensin [GenBank: AAW01790]; *Cupiennius salei* Defensin, *Polybetes pythagoricus* Defensin, *Tegenaria atrica* Defensin [7]
Scorpio maurus Maurotoxin	[PDB: 1TXM]	Maurotoxin	*Pandinus imperator* Pi4, Pi7 non-toxic peptide [PDB: 1N8M, 1QKY]
Tityus serrulatus Neurotoxin Ts1	[PDB: 1B7D]	Neurotoxin Ts1	*Schistosoma mansoni* [GenBank: EX499261, EFX499213]
Tityus serrulatus Tityustoxin	[PDB: 1HP2]	Tityustoxin	
Tityus serrulatus Ts kappa	[PDB: 1TSK]	Ts kappa	
Tityus serrulatus Butantoxin	[PDB: 1C56]	Butantoxin	
**Crustacea**			
Daphnia pulex	[GenBank: EFX83704]	Daphnia EFX83704	
Daphnia pulex	[GenBank: FE421610]	Daphnia FE421610	*Daphnia pulex* [GenBank: EFX73794]
Daphnia pulex	[GenBank: FE339229]	Daphnia FE339229	
Daphnia pulex	[GenBank: FE339755]	Daphnia FE339755	
Litopenaeus vannamei	[GenBank: FE088090]	Litopenaeus FE088090	
**Myriapoda**			
Archispirostreptus gigas	[GenBank: FN197329]	Agigas	
Scolopendra canidens Defensin	[GenBank: AAW01788]	Scanidens	
**Tardigrada**			
Hypsibius dujardini Defensin 1	[TardiBASE: **HDC02701**; GenBank: CK326474, CK326631, CK326718, CK326749, CO741228]	Hd-defensin 1	
Hypsibius dujardini Defensin 2	[GenBank: **CO741284**, CD449294, CD449920, CO508161]	Hd-defensin 2	*Hypsibius dujardini* Defensin 7 [GenBank: CO741157]
Hypsibius dujardini Defensin 3	[GenBank: **CO741737**, CO742078, CK326534]	Hd-defensin 3	
Hypsibius dujardini Defensin 4	[GenBank: **CD449538,** CO501858]	Hd-defensin 4	

Hypsibius dujardini Defensin 5	[GenBank: CO507946]	Hd-defensin 5	*Hypsibius dujardini* Defensin 6 [GenBank: CO508499]
Hypsibius dujardini Defensin 8	[GenBank: CO741522]	Hd-defensin 8	
Milnesium tardigradum Defensin 1	[GenBank: **GR863249,** GR861903]	Mt-defensin 1	
Milnesium tardigradum Defensin 2	[GenBank: GR861547]	Mt-defensin 2	
Milnesium tardigradum Defensin 3	[GenBank: GR860666]	Mt-defensin 3	
Milnesium tardigradum Defensin 4	[GenBank: GR862104]	Mt-defensin 4	
Milnesium tardigradum Defensin 5	[GenBank: GR861858]	Mt-defensin 5	
Milnesium tardigradum Defensin 6	[GenBank: GR861612]	Mt-defensin 6	
Milnesium tardigradum Defensin 8	[GenBank: GR864191]	Mt-defensin 8	*Milnesium tardigradum* Defensin 7 [GenBank: GR865187]
**Onychophora**			
Peripatopsis sedgwicki	[GenBank: FN237260]	Ps_FN237260	
Peripatopsis sedgwicki	[GenBank: FN236522]	Ps_FN236522	*Peripatopsis sedgwicki* [GenBank: FN243200]
Peripatopsis sedgwicki	[GenBank: FN241528]	Ps_FN241528	
Peripatopsis sedgwicki	[GenBank: FN239939]	Ps_FN239939	
Peripatopsis sedgwicki	[GenBank: **FN233877**, FN242990, FN242919]	Ps_FN233877	
Peripatopsis sedgwicki	[GenBank: **FN239399**, FN243038, FN242834]	Ps_FN239399	
Peripatopsis sedgwicki	[GenBank: FN243111]	Ps_FN243111	*Peripatopsis sedgwicki* [GenBank: FN242191]
**Nematoda**			
Ancylostoma caninum	[NEMBASE: ACC17687]	ACC17687	
Ancylostoma ceylanicum	[NEMBASE: AYC00542]	AYC00542	
Ascaris lumbricoides	[NEMBASE: ALC00177]	ALC00177	*Ascaris suum* [NEMBASE: ASC03066]; *Toxocara canis* [NEMBASE: TCC00565contig2]

Ascaris suum ASABF-alpha	[GenBank: BAA89497]	ASABF-alpha	*Ascaris suum* ASABF-beta [GenBank: BAC00497]; *Ascaris lumbricoides* [NEMBASE: ALC00086]; *Toxocara canis* [NEMBASE: TCC00437]
Ascaris suum ASABF-6Cys-alpha	[GenBank: BAC41496]	ASABF-6Ca	
Ascaris suum ASABF-gamma	[GenBank: BAC00498]	ASABF-gamma	*Ascaris lumbricoides* [NEMBASE: ALC00180]; *Parastrongyloides trichosuri* [NEMBASE: PTC03950]
Ascaris suum ASABF-delta	[GenBank: BAC00499]	ASABF-delta	*Toxocara canis* [NEMBASE: TCC02268]
Ascaris suum ASABF-epsilon	[GenBank: BAC41495]	ASABF-epsilon	
Ascaris suum ASABF-zeta	[GenBank: BAC57992]	ASABF-zeta	
Ascaris suum	[NEMBASE: ASC03701]	ASC03701	
Ascaris suum	[NEMBASE: ASC17142]	ASC17142	*Parastrongyloides trichosuri* [NEMBASE: PTC03906]
Bursaphelenchus xylophilus	[NEMBASE: BXC00487]	BXC00487	
Caenorhabditis brenneri	[WormBase: CBN15906]	CBN15906	
Caenorhabditis brenneri	[WormBase: CBN16351]	CBN16351	
Caenorhabditis brenneri	[WormBase: CBN22403]	CBN22403	
Caenorhabditis brenneri	[WormBase: CBN00173]	CBN00173	*Caenorhabditis brenneri* [WormBase: CBN15401]
Canorhabditis briggsae CbrABF2	[WormBase: CBG11981]	CbrABF2	
Canorhabditis briggsae	[WormBase: CBG09786]	CBG09786	*Canorhabditis briggsae* [WormBase: CBG09224]
Canorhabditis briggsae	[WormBase: CBG21681]	CBG21681	
Caenorhabditis elegans CeABF1	[NCBI Reference Sequence: NP_491253]	CeABF1	
Caenorhabditis elegans CeABF2	[NCBI Reference Sequence: NP_491252]	CeABF2	

Caenorhabditis elegans CeABF4	[NCBI Reference Sequence: NP_507965]	CeABF4	*Caenorhabditis elegans* CeABF3, CeABF5 [NCBI Reference Sequence: NP_506950, NP_510136]; *C. elegans* [WormBase: Y38H6C.23]; *C. brenneri* [WormBase: CBN14150]; *C. briggsae* CbrABF5 [WormBase: CBG00070]; *C. japonica* [WormBase: CJA09228]; *C. remanei* CreABF5 [WormBase: CRE24112], [NEMBASE: CRC00359]; *C.sp. 5 AC-2008* [NEMBASE: CSC00683]
Caenorhabditis elegans CeABF6	[NCBI Reference Sequence: NP_741914]	CeABF6	*Caenorhabditis brenneri* CbnABF6 [WormBase: CBN15115]; *C. briggsae* CbrABF6 [WormBase: CBG00069]; *C. japonica* CjpABF6 [WormBase: CJA03860]; *C. remanei* CreABF6 [WormBase: CRE24111]; *C. sp. 5 AC-2008* [NEMBASE: CSC01351]
Caenorhabditis elegans fipr-29	[WormBase: H06I04.7]	Ce_fipr29	
Caenorhabditis japonica	[NEMBASE: CJC05393]	CJC05393	
Caenorhabditis japonica	[NEMBASE: CJC08906]	CJC08906	
Caenorhabditis remanei	[WormBase: CRE07955]	CRE07955	*Caenorhabditis brenneri* [NEMBASE: CBC04439]
Caenorhabditis remanei	[WormBase: CRE25171]	CRE25171	
Caenorhabditis remanei	[WormBase: CRE02801]	CRE02801	

Caenorhabditis remanei Cremycin 5	[GenBank: AEM44806]	Cremycin 5	*Caenorhabditis remanei* Cremycins 1-3, 6, 7, 9-13 [GenBank: AEM44803, AEM44804, AEM44805, AEM44807, AEM44808, AEM44809, CRE14278, AEM44810, CRE14279, AEM44811], Cremycins 3, 8, 14 [8], [WormBase: CRE14287]
Caenorhabditis remanei Cremycin 15 mislabeled as Cremycin 14	[GenBank: AEM44812]	Cremycin 15	
Meloidogyne hapla Mehamycin	[NEMBASE: MHC07481]	Mehamycin	*Meloidogyne hapla* [NEMBASE: MHC08122]
Meloidogyne hapla	[NEMBASE: MHC08639]	MHC08639	
Meloidogyne hapla	[NEMBASE: MHC07477]	MHC07477	*Meloidogyne hapla* [NEMBASE: MHC08815]
Meloidogyne javanica	[NEMBASE: MJC04218]	MJC04218	
Necator americanus	[NEMBASE: NAC00042]	NAC00042	*Ancylostoma caninum* [NEMBASE: ACC38241]; *Ancylostoma ceylanicum* [NEMBASE: AYC00474]; *Haemonchus contortus* [NEMBASE: HCC02002, HCC02570, HCC06231]; *Nippostrongylus brasiliensis* [NEMBASE: NBC02237]
Necator americanus	[GenBank: GE625123]	Necator_GE625123	
Panagrolaimus superbus	[NEMBASE: PSC02929]	PSC02929	
Pratylenchus penetrans	[NEMBASE: PEC00189]	PEC00189	
Pristionchus pacificus	[WormBase: PPA22162]	PPA22162	*Pristionchus pacificus* [WormBase: PPA01653]
Pristionchus pacificus	[NEMBASE: PPC03474]	PPC03474	
Rotylenchulus reniformis	[GenBank: GT737647]	Rotylenchus_GT737647	
Teladorsagia circumcincta	[NEMBASE: TDC00905]	TDC00905	

Toxascaris leonina	[NEMBASE: TLC00068]	TLC00068	
Toxocara canis	[NEMBASE: TCC02370]	TCC02370	
Toxocara canis	[NEMBASE: TCC00389]	TCC00389	*Toxascaris leonina* [NEMBASE: TLC00308]
Xiphinema index	[NEMBASE: XIC00639]	XIC00639	*Xiphinema index* [NEMBASE: XIC04611]
**Bivalvia**			
Crassostrea gigas Defensin	[GenBank: AJ565499]	Cg Defensin	*Cristaria plicata* Defensin [GenBank: AGG86914]; *Hyriopsis cumingii* HcDef1, HcDef5 [9]
Crassostrea virginica Defensin	[Swiss-Prot: P85008]	Cv Defensin	*Mytilus edulis* Defensins A, B [Swiss-Prot: P81610, P81611]
Dreissena polymorpha Defensin (Dpd)	[GenBank: ACZ02692]	Dpd	*Haliotis discus discus* Defensin [Swiss-Prot: D3UAH2]
Hyriopsis cumingii HcDef3	[GenBank: AEX88475]	HcDef3	*Hyriopsis cumingii* HcDef2, HcDef4, HcDef6 [9]
Hyriopsis cumingii Hc theromacin	[GenBank: GU123628]	Hc theromacin	
Mytilus edulis Mytilin A	[Swiss-Prot: P81612]	Mytilin A	*Mytilus edulis* Mytilin B [Swiss-Prot: P81613]; *M. galloprovincialis* Mytilins B, D [GenBank: AAD45013, GenBank: ACF21701], Mytilins C, G1 [10]
Mytilus galloprovincialis MGD-1	[Swiss-Prot: P80571]	MGD-1	*Mytilus galloprovincialis* MGD2 [Swiss-Prot: Q9U6U0]
Mytilus galloprovincialis Myticin A	[Swiss-Prot: P82103]	Myticin A	*Mytilus galloprovincialis* Myticin B [Swiss-Prot: P82102]
Mytilus galloprovincialis Mytimacin 1	[GenBank: CCC15015]	Mytimacin 1	*Mytilus galloprovincialis* Mytimacin 4

			[GenBank: CCC15018]
Mytilus galloprovincialis Mytimacin 2	[GenBank: CCC15016]	Mytimacin 2	
Mytilus galloprovincialis Mytimacin 3	[GenBank: CCC15017]	Mytimacin 3	
Mytilus galloprovincialis Mytimacin 5	[GenBank: CCC15019]	Mytimacin 5	
Ruditapes philippinarum Defensin	[GenBank: AEK78067]	Rp Defensin	
**Gastropoda**			
Achatina fulica Mytimacin-AF	[GenBank: AFR36920]	Mytimacin-AF	
Aplysia californica	[NCBI Reference Sequence: XP_005103372]	Aplysia XP_005103372	
Aplysia californica	[NCBI Reference Sequence: NP_001191629]	Aplysia NP_001191629	
Aplysia californica	[NCBI Reference Sequence: XP_005095540]	Aplysia XP_005095540	
Littorina saxatalis	[GenBank: FR864485]	Littorina	
**Echinodermata**			
Asterina pectinifera/Patiria pectinifera	[GenBank: DB418655]	Asterina	*Asterina pectinifera/Patiria pectinifera* [GenBank: DB424270]
Patiria miniata	[GenBank: EX453588]	Patiria	
**Annelida**			
Hirudo medicinalis Neuromacin	[Swiss-Prot: A8V0B3]	Neuromacin	
Theromyzon tessulatum Theromacin	[GenBank: AAR12065]	Tt-theromacin	*Hirudo medicinalis* Theromacin [Swiss-Prot: A8I0L8]
**Platyhelminthes**			
Schistosoma mansoni	[GenBank: **EX499237**, EX499238, EX499230]	Schisto EX499237	
Schistosoma mansoni	[GenBank: **EX499243**, EX499221, EX499222, EX499244]	Schisto EX499243	*Schistosoma mansoni* [GenBank: EX499256]
**Plantae**			
Arabidopsis halleri Ah-PDF1.1	[GenBank: AAY27736]	Ah-PDF1.1	
Arabidopsis thaliana PDF1.1	[NCBI Reference Sequence: NP_565119]	PDF1.1	*Arabidopsis thaliana* PDF1.2 [Swiss-Prot: Q9FI23], PDF 1.3 [NCBI Reference

			Sequence: NP_180171]
Arabidopsis thaliana Trypsin inhibitor	[PDB: 1JXC]	Trypsin inhibitor	
Beta vulgaris AX1	[Swiss-Prot: P81493]	AX1	*Beta vulgaris* AX2 [Swiss-Prot: P82010]
Brassica campestris BSD1	Sequence from [11]	BSD1	
Brassica oleracea PCP-A1	[GenBank: CAA06464]	PCP-A1	
Capsicum annuum CaDEF1	[GenBank: AAL35366]	CaDEF1	
Capsicum annuum J1-1	[Swiss-Prot: Q43413]	J1-1	
Cassia fistula Peptide 5144	[12]	Cassia 5144	
Dahlia merckii Dm-AMP1	[GenBank: AAB34972]	DmAMP1	*Aesculus hippocastanum* Ah-AMP1 [GenBank: AAB34970]; *Clitoria ternatea* Ct-AMP1 [GenBank: AAB34971]; *Helianthus annuus* HaDEF1 [GenBank: AF364865]; *Stellaria media* Sm-AMP-D1 [13]
Echinocloa crusgalli Ec-AMP-D1	[Swiss-Prot: P86518]	Ec-AMP-D1	*Echinocloa crusgalli* Ec-AMP-D2 [14]
Elaeis guineensis EGAD1	[GenBank: AF322914]	EGAD1	
Glycine max Defensin	[Swiss-Prot: Q07502]	Soybean defensin	*Vigna unguiculata* Cp-thionin I [Swiss-Prot: P83399]
Helianthus annuus SD2	[Swiss-Prot: P82659]	Helianthus SD2	
Heuchera sanguinea Hs-AMP1	[Swiss-Prot: P0C8Y5]	Hs-AMP1	

Hordeum vulgare Gamma-hordothionin	[Swiss-Prot: P20230]	Gamma-hordothionin	*Nicotiana megalosiphon* Nm-Def02/Nmdef2 [GenBank: ACR46857]; *Triticum turgidum* Gamma1-purothionin, Gamma-2 purothionin [PRF: 227202, 227203]
Ipomoea batatas SPD1	[GenBank: AY552546]	SPD1	
Mendicago trucatula MtDEF2	[GenBank: AAQ91290]	MtDEF2	*Arachis hypogaea* Peanut allergens Arah12 and Arah 13 [GenBank: EY396089, EY396019]; *Cassia fistula* Peptide 5459 [12]; *Hardenbergia violacea* HvAMP1 [15]; *Lens culinaris* LcDef [GenBank: ABP04037]; *Medicago sativa* MsDEF1 [GenBank: AAG40321]; *Pachyrrhizus erosus* SPE10 [GenBank: AAT80338]; *Phaseolus limensis* BLBAFP [16]; *Phaseolus vulgaris* PvD1 [GenBank: ADR30066], WCBAFP [17]; *Pisum sativum* PsD1/Psd1, PsD2/Psd2 [Swiss-Prot: P81929, P81930]; *Tephrosia villosa* TvD1 [GenBank: AAX86993]; *Trigonella foenum-graecum* Tfgd1[GenBank: AAO72632]; *Vicia faba* Defensin [GenBank: ACI02059]; *Vigna unguiculata* VUDEF [18]

Nicotiana alata NaD1	[Swiss-Prot: Q8GTM0]	NaD1	*Capsicum chinense* Gamma-thionin [GenBank: AAD21200]; *Petunia hybrida* PhD1, PhD2 [Swiss-Prot: Q8H6Q1, Q8H6Q0]; *Solanum lycopersicum* DEF2 [GenBank: AW623541]
Nigella sativa Ns-D1	[Swiss-Prot: P86972]	Ns-D1	*Nigella sativa* Ns-D2 [Swiss-Prot: P86973]
Pentadiplandra brazzeana Brazzein (sweet tasting protein)	[Swiss-Prot: P56552]	Brazzein	
Pinus sylvestris PsDef1	[Swiss-Prot: A4L7R7]	PsDef1	*Ginkgo biloba* Gbd [GenBank: AAU04859]
Prunus persica Pp-def1	[GenBank: AAL85480]	Pp-def1	
Raphanus sativus RsAFP1	[GenBank: AAA69541]	RsAFP1	*Raphanus sativus* RsAFP2 [GenBank: AAA69540]
Saccharum officinarum Sd1	[GenBank: CA112870]	Sd1	*Saccharum officinarum* Sd3 [GenBank: CA259771]
Saccharum officinarum Sd2	[GenBank: CA095771]	Sd2	*Sorghum bicolor* SIalpha2, SIalpha3 [Swiss-Prot: P21924, P21925]; *Zea mays* Gamma-1-zeathionin [Swiss-Prot: P81008]
Saccharum officinarum Sd4	[GenBank: CA259589]	Sd4	
Saccharum officinarum Sd5	[GenBank: CA297803]	Sd5	
Spinacea oleracea SoD2	[Swiss-Prot: P81571]	SoD2	
Trichosanthes kirilowii TDEF	[GenBank: ABF74600]	TDEF	
Triticum aestivum TAD1	[GenBank: BAC10287]	TAD1	*Triticum kiharae* Tk-AMP-D1 [Swiss-Prot: P84963]; *Triticum monococcum* Tm-AMP-D1.2 [Swiss-Prot: P84964]

Vigna radiata VrD1	[GenBank: AAR08912]	VrD1	*Vigna angularis* VaD1 [19]; *Vigna radiata* VrCRP [20]; *Vigna unguiculata* Cp-thionin II [21]
Vitis vinifera Vv-AMP1	[22]	VvAMP1	
Wasabia japonica WT1	[GenBank: AB012871]	WT1	*Lepidium meyenii* Lm-def [GenBank: AAV85992]
Zea mays Gamma-2-zeathionin, PDC-1	[Swiss-Prot: P81009]	Gamma2-zeathionin	*Hordeum vulgare* Omega-hordothionin Sequence from [23]; *Saccharum officinarum* Sd6 [GenBank: CA188998]; *Sorghum bicolor* SIalpha1 [Swiss-Prot: P21923]
Zea mays ZmESR-6	[NCBI Reference Sequence: NP_001105777]	ZmESR6	
**Fungi**			
Ajellomyces capsulatus/Histoplasma capsulatum Acapsin 1, 2	[24]	Acapsin 1	*Ajellomyces capsulatus/Histoplasma capsulatum* Acasin [GenBank: AAJI01002159]; *Ajellomyces dermatitidis/Blastomyces dermatitidis* Adersin 1, 2 [24]
Arthroderma otae/Microsporum canis Micasin	[GenBank: JN014007]	Micasin	*Arthroderma benhamiae* Arbesin, *Trichophyton rubrum* Trirusin, *T. tonsurans* Tritosin, *T. verrucosum* Trivesin [24]
Arthroderma otae/Microsporum canis Micasin 1	[GenBank: JN014008]	Micasin 1	
Aspergillus nidulans Anisin 1	[NCBI Reference Sequence: XP_662650]	Anisin 1	*Aspergillus nidulans* Anisin 2 [GenBank: AACD01000108]; *Laccaria bicolor* Labisin

			[24]
Aspergillus terreus Atesin 1	[GenBank: AAJN01000157]	Atesin 1	*Aspergillus terreus* Atesin 2 [NCBI Reference Sequence: XP_001209108]
Aspergillus terreus Atesin 3	[GenBank: EF541165]	Atesin 3	
Chaetomium globosum Cglosin2	[GenBank: AAFU01000488]	Cglosin 2	*Arthroderma gypseum* Agysin [24]; *Aspergillus flavus* Aflasin 3 [25]
Neosartorya fischeri Nefisin	[24]	Nefisin	*Aspergillus clavatus* Aclasin [24]
Neosartorya fischeri Nefisin 2, N-term	[GenBank: AAKE03000016]	Nefisin 2N	*Neosartorya fischeri* Nefisin 1N [25]; *Aspergillus clavatus* Aclasin N [GenBank: AAKD03000014]; *A. flavus* Aflasin 1N [25]; *A. fumigatus* Afusin N [NCBI Reference Sequence: XP_748991]; *A. oryzae* Aorsin N [GenBank: BAE56652]; *Chaetomium globosum* Cglosin 1N [GenBank: EAQ86375]

Neosartorya fischeri Nefisin 2, C-term	[GenBank: AAKE03000016]	Nefisin 2C	*Neosartorya fischeri* Nefisin 1C [25]; *Aspergillus clavatus* Aclasin C [GenBank: AAKD03000014]; *A. flavus* Aflasin 1C [25]; *A. fumigatus* Afusin C [NCBI Reference Sequence: XP_748991]; *A. oryzae* Aorsin C [GenBank: BAE56652]; *Chaetomium globosum* Cglosin 1C [GenBank: EAQ86375]
Penicillium chrysogenum Pechrysin	[24]	Pechrysin	
Pseudoplectania nigrella Plectasin	[Swiss-Prot: Q53I06]	Plectasin	
Rhizopus oryzae Rorsin 1	[GenBank: AACW02000043]	Rorsin 1	*Rhizopus oryzae* Rorsin 2 [GenBank: AACW02000259]
Verticillium albo-atrum Vasin	[24]	Vasin	*Aspergillus fumigatus (flavus?)* Aflasin 2 [24, 25] *Verticillium dahliae* Vedasin [24]
**Human**			
Homo sapiens Drosomycin-like defensin (DLD)	[GenBank: AK024601]	Hs-DLD	

References

1. Lauth X, Nesin A, Briand J-P, Roussel J-P, Hetru C. Isolation, characterization and chemical synthesis of a new insect defensin from *Chironomus plumosus* (Diptera). Insect Biochem Molec Biol. 1998 December;28(12):1059-66.
2. Pöppel A-K, Vogel H, Wiesner J, Vilcinskas A. Antimicrobial Peptides Expressed in Medicinal Maggots of the Blow Fly *Lucilia sericata* Show Combinatorial Activity against Bacteria. Antimicrobial Agents and Chemotherapy. 2015;59(5):2508-14.
3. Gao B, Zhu S. Identification and characterization of the parasitic wasp *Nasonia* defensins: Positive selection targeting the functional region? Developmental and Comparative Immunology. 2010 June;34(6):659-68.
4. Taguchi S, Bulet P, Hoffmann JA. A novel insect defnsin from the ant *Formica rufa*. Biochimie. 1998 April;80(4):343-6.
5. Zhou H, Kong Y, Wang H, Tianhua Y, Feng F, Bian J, et al. A defensin-like antimicrobial peptide from the venoms of spider, *Ornithoctonus hainana*. Journal of Peptide Science. 2011 April 2011;17:540-4.

6. Chrudimská T, Slaninová J, Rudenko N, Růžek D, Grubhoffer L. Functional characterization of two defensin isoforms of the hard tick *Ixodes ricinus*. Parasites & Vectors. 2011;4:63.
7. Baumann T, Kuhn-Nentwig L, Largiadèr CR, Nentwig W. Expression of defensins in non-infected araneomorph spiders. Cell Mol Life Sci. 2010 Aug;67(15):2643-51.
8. Zhu S, Gao B. Nematode-derived drosomycin-type antifungal peptdies provide evidence for plant-to-ecdysozoan horizontal transfer of a disease resistance gene. Nature Communications. 2014;5.
9. Ren Q, Li M, Zhang C-Y, Chen K-P. Six defensins from the triangle-shell pearl mussel *Hyriopsis cumingii*. Fish and Shellfish Immunology. 2011;31:1232-8.
10. Mitta G, Vandenbulcke F, Hubert F, Salzet M, Roch P. Involvement of Mytilins in Mussel Antimicrobial Defense. Journal of Biological Chemistry. 2000 April 28;275(17):12954-62.
11. Park HC, Kang YH, Chun HJ, Koo JC, Cheong YH, Kim CY, et al. Characterization of a stamen-specific cDNA encoding a novel plant defensin in Chinese cabbage. Plant Molecular Biology. 2002 Sept.;50(1):59-69.
12. Wijaya R, Neumann GM, Condron R, Hughes AB, Polya GM. Defense proteins from seed of Cassia fistula include a lipid transfer protein homologue and a protease inhibitory defensin. Plant Science. 2000;159:243-55.
13. Slavokhotova AA, Odintsova TI, Rogozhin EA, Musolyamov AK, Andreev YA, Grishin EV, et al. Isolation, molecular cloning and antimicrobial activity of novel defensins from common chickweed (*Stellaria media* L.) seeds. Biochimie. 2011 March;93(3):450-6.
14. Odintsova TI, Rogozhin EA, Baranov Y, Musolyamov AK, Yalpani N, Egorov TA, et al. Seed defensins of barnyard grass *Echinochloa crusgalli* (L.) Beauv. Biochimie. 2008 Nov-Dec;90(11-12):1667-73.
15. Harrison SJ, Marcus JP, Goulter KC, Green JL, Maclean DJ, Manners JM. An Antimicrobial Peptide from the Australian Native *Hardenbergia violacea* Provides the First Functionally Characterised Member of a Subfamily of Plant Defensins. Australian Journal of Plant Physiology. 1997;24(5):571-8.
16. Wang HX, Ng TB. An antifungal peptide from baby lima bean. Applied Microbiology and Biotechnology. 2006 Dec.;73(3):576-81.
17. Wong JH, Zhang XQ, Wang HX, Ng TB. A mitogenic defensin from white cloud beans (*Phaseolus vulgaris*). Peptides. 2006 Sept.;27(9):2075-81.
18. Carvalho AO, Machado OLT, Da Cunha M, Santos IS, Gomes VM. Antimicrobial peptides and immunolocalization of a LTP in *Vigna unguiculata* seeds. Plant Physiology and Biochemistry. 2001 Feb.;39(2):137-46.
19. Chen G-H, Hsu M-P, Tan C-H, Sung H-Y, Kuo CG, Fan M-J, et al. Cloning and Characterization of a Plant Defensin VaD1 from Azuki Bean. Journal of Agricultural and Food Chemistry. 2005 Feb. 23;53(4):982-8.
20. Chen K-C, Lin C-Y, Kuan C-C, Sung H-Y, Chen C-S. A Novel Defensin Encoded by a Mungbean cDNA Exhibits Insecticida Activity against Bruchid. Journal of Agricultural and Food Chemistry. 2002 Dec. 4;50(25):7258-63.
21. Franco OL, Murad AM, Leite JR, Mendes PAM, Prates MV, Bloch C, Jr. Identification of a cowpea g-thionin with bactericidal activity. FEBS Journal. 2006 August;273(15):3489-97.
22. de Beer A, Vivier MA. Vv-AMPI, a ripening induced peptide from *Vitis vinifera* shows strong antifungal activity. BMC Plant Biology. 2008 July 8;8:75.

23. Méndez E, Rocher A, Calero M, Girbés T, Citores L, Soriano F. Primary structure of ω-hordothionin, a member of a novel family of thionins from barley endosperm, and its inhibition of protein synthesis in eukaryotic and prokaryotic cell-free systems. Eur J Biochem. 1996;239:67-73.
24. Zhu S, Gao B, Harvey PJ, Craik DJ. Dermatophytic defensin with antiinfective potential. PNAS. 2012;109(22):8495-500.
25. Zhu S. Discovery of six families of fungal defensin-like peptides provides insights into origin and evolution of the CSab defensins. Mol Immunol. 2008;45:828-38.
