# Supplementary material for: Establishing a reference array for the CS-αβ superfamily of defensive peptides
Source: BMC Res Notes. 2016 Nov 18;9:490. doi: 10.1186/s13104-016-2291-0 (PMC5116183; doi:10.1186/s13104-016-2291-0)
Supplement: Supplementary file 4 — Additional file 4: Table S3. Accession numbers for query sequences used in BLAST searches. [file 13104_2016_2291_MOESM4_ESM.pdf]

Additional File 4: Table S3.
Accession numbers for query sequences used in BLAST searches.

**Species (by major taxonomic group) and name of peptide (if one has been given)**	**Accession**
**Bacteria**	
Anaeromyxobacter dehalogenans AdDLP	[NCBI Reference Sequence: WP_011422871]
**Cnidaria**	
Hydra magnipapillata Hydramacin	[GenBank: ABE26989]
**Porifera**	
Subiteres domuncula ASABF-related peptide	[GenBank: CCC55928]
**Hexapoda**	
Aeschna cyanea Defensin	[Swiss-Prot: P80154]
Apis mellifera Royalisin	[Swiss-Prot: P17722]
Drosophila melanogaster Defensin	[Swiss-Prot: P36192]
Drosophila melanogaster Drosomycin	[Swiss-Prot: P41964]
Heliothis virescens Heliomicin	[GenBank: ACR78445]
Macrotermes barneyi Termicin	[GenBank: ACO90349]
Protophormia terraenovae Phormicin A	[Swiss-Prot: P10891]
Triatoma brasiliensis Defensin 1	[Swiss-Prot: Q4VSI0]
**Arachnida**	
Centruroides limpidus Cll-dlp	[Swiss-Prot: Q6GU94]
Centruroides sculpturatus CsEv2 neurotoxin	[Swiss-Prot: P01493]
Haemaphysalis longicornis Longicin	[Swiss-Prot: Q58A47]
Leiurus quinquestriatus Defensin	[Swiss-Prot: P41965]
*Mesobuthus martensii (Buthus)*K-channel toxin	[Swiss-Prot: Q9NII6]
Ornithodoros moubata Defensin A	[Swiss-Prot: Q9BLJ3]
Pandinus imperator Scorpine	[Swiss-Prot: P56972]
**Nematoda**	
Ascaris suum ASABF-alpha	[GenBank: BAA89497]
Ascaris suum ASABF-6Cys-alpha	[GenBank: BAC41496]
Ascaris suum ASABF-epsilon	[GenBank: BAC41495]
Caenorhabditis elegans CeABF1	[NCBI Reference Sequence: NP_491253]
Caenorhabditis elegans CeABF2	[NCBI Reference Sequence: NP_491252]
Caenorhabditis elegans CeABF4	[NCBI Reference Sequence: NP_507965]
Caenorhabditis elegans CeABF5	[NCBI Reference Sequence: NP_510136]
Caenorhabditis elegans CeABF6	[NCBI Reference Sequence: NP_741914]
**Bivalvia**	
Crassostrea gigas Defensin	[GenBank: AJ565499]
Crassostrea virginica Defensin	[Swiss-Prot: P85008]
Mytilus galloprovincialis MGD-1	[Swiss-Prot: P80571]
Mytilus galloprovincialis Myticin A	[Swiss-Prot: P82103]
**Gastropoda**	
Haliotis discus discus Defensin	[Swiss-Prot: D3UAH2]
**Annelida**	
Hirudo medicinalis Neuromacin	[Swiss-Prot: A8V0B3]
Hirudo medicinalis Theromacin	[Swiss-Prot: A8I0L8]
**Plantae**	
Nicotiana alata NaD1	[Swiss-Prot: Q8GTM0]
**Fungi**	
Pseudoplectania nigrella Plectasin	[Swiss-Prot: Q53I06]
