# Supplementary material for: Establishing a reference array for the CS-αβ superfamily of defensive peptides
Source: BMC Res Notes. 2016 Nov 18;9:490. doi: 10.1186/s13104-016-2291-0 (PMC5116183; doi:10.1186/s13104-016-2291-0)
Supplement: Supplementary file 5 — Additional file 5: Table S4. CS-αβ sequences considered in this study. In cases where a sequence is represented by more than one accession number, the sequence used is shown in bold. [file 13104_2016_2291_MOESM5_ESM.pdf]

Additional File 5: Table S4. CS-αβ sequences considered in this study. In cases where a sequence is represented by more than one accession number, the sequence used is shown in bold.

**Species (grouped by major taxonomic group) and name of peptide (if one has been given)**	**Accession number**
**Bacteria**	
Anaeromyxobacter dehalogenans AdDLP	[NCBI Reference Sequence: WP_011422871]
**Cnidaria**	
Acropora palmata	[GenBank: **GW203080,** GW203081, GW208862, GW208863]
Anemonia viridis	[GenBank: FK740859]
Anemonia viridis	[GenBank: **FK731853**, FK754575, FK756642, FK751627, FK747619, FK737168]
Clytia hemisphaerica	[GenBank: **FP967549**, FK985645]
Clytia hemisphaerica	[GenBank: FP943555]
Clytia hemisphaerica	[GenBank: FP966428]
Hydra magnipapillata Hydramacin	[GenBank: ABE26989]
Metridium senile	[GenBank: **FC831074**, FC832453, FC840147, FC826793, FC834614, FC826372, FC837794]
Montastraea faveolata	[GenBank: **GW270650**, GW270651]
Montastraea feveolata	[GenBank: GW256715]
Podocoryna carnea	[GenBank: DY451332]
**Porifera**	
Leucetta chagosensis	[GenBank: GO092849]
Leucetta chagosensis	[GenBank: GO091294]
Subiteres domuncula ASABF-related peptide	[GenBank: CCC55928]
**Hexapoda**	
Acalolepta luxuriosa AlCRP	[GenBank: AB104817]
Acalolepta luxuriosa Defensin 1	[Swiss-Prot: Q9BK52]
Aedes aegypti Defensin A	[Swiss-Prot: P91793]
Aedes aegypti Defensin B	[Swiss-Prot: P81602]
Aedes aegypti Defensin C	[Swiss-Prot: P81603]
Aeschna cyanea Defensin	[Swiss-Prot: P80154]
Allomyrina dichotoma (Trypoxylus dichotomus) Defensin	[Swiss-Prot: Q10745]
Anomala cuprea Defensin A	[Swiss-Prot: P83669]
Anomala cuprea Defensin B	[Swiss-Prot: P83668]
Anopheles gambiae Defensin	[Swiss-Prot: Q17027]
Apis mellifera Royalisin	[Swiss-Prot: P17722]
Apis mellifera Defensin 2	[NCBI Reference Sequence: NP_001011638]
Archeoprepona demophon ARD1	[Swiss-Prot: P84156]
Bombus pascuorum Defensin	[Swiss-Prot: P81462]
Bombyx mori Defensin A	[NCBI Reference Sequence: NP_001037370]

Bombyx mori Defensin B	[GenBank: BAG71131]
Bombyx mori Defensin-like protein	[Swiss-Prot: Q45RF8]
Chironomus plumosus Defensin A	[1]
Chironomus plumosus Defensin B	[1]
Copris tripartitus Coprisin	[GenBank: ABP97087]
Drosophila melanogaster Defensin	[Swiss-Prot: P36192]
Drosophila melanogaster Drosomycin	[Swiss-Prot: P41964]
Drosophila melanogaster Drosomycin 2	[GenBank: ABY84135]
Eristalis tenax Eristalin	[GenBank: AM706420]
Formica aquilonia Defensin	[GenBank: AAX20157]
Formica rufa Defensin	[2]
Galleria mellonella Gallerimycin	[Swiss-Prot: Q8MVY9]
Galleria mellonella Defensin	[Swiss-Prot: P85213]
Glossina morsitans Defensin A	[Swiss-Prot: Q8WTD4]
Heliothis virescens Heliomicin	[GenBank: ACR78445]
Holotrichia diomphalia Holotricin 1	[Swiss-Prot: Q7M426]
Lucilia sericata Lucifensin 2	[GenBank: ADI87383]
Lucilia sericata Lucifensin 1	[3]
Lucilia sericata Lucifensin 3	[3]
Lucilia sericata Lucifensin 4	[3]
Lucilia sericata Lucifensin 6	[3]
Lucilia sericata Lucifensin 7	[3]
Macrotermes barneyi Termicin	[GenBank: ACO90349]
Mamestra brassicae Defensin	[GenBank: AAL69980]
Nasonia vitripennis Navidefensin2-2	[4]
Oryctes rhinoceros Defensin	[Swiss-Prot: O96049]
Phlebotomus duboscqi Defensin	[Swiss-Prot: P83404]
Protophormia terraenovae Phormicin A	[Swiss-Prot: P10891]
Protophormia terraenovae Phormicin B (G86R)	[Swiss-Prot: P10891]
Pseudacanthotermes spiniger Termicin	[Swiss-Prot: P82321]
Pyrrhocoris apterus Defensin	[Swiss-Prot: P37364]
Pyrrhocoris apterus Defensin 2	[GenBank: AGI17576]
Rhodnius prolixus Defensin A	[GenBank: AAO74624]
Rhodnius prolixus Defensin B	[GenBank: AAO74625]
Rhodnius prolixus Defensin C	[GenBank: AAO74626]
Samia cynthia ricini Scr-gallerimycin	[GenBank: BAG12297]
Sarcophaga peregrina Sapecin A	[Swiss-Prot: P18313]
Sarcophaga peregrina Sapecin B	[Swiss-Prot: P31529]
Sarcophaga peregrina Sapecin C	[Swiss-Prot: P31530]
Spodoptera frugiperda Gallerimycin	[Swiss-Prot: Q6XD81]
Spodoptera frugiperda Spodoptericin	[GenBank: AAQ18895]
Spodoptera frugiperda Sf-cobatoxin	[GenBank: AAP69839]
Spodoptera littoralis SpliDef	[GenBank: HQ603825]
Spodoptera litura Sl-gallerimycin	[GenBank: JG406586]
Stomoxys calcitrans Smd1	[Swiss-Prot: O16136]
Stomoxys calcitrans Smd2	[Swiss-Prot: O16137]

Tenebrio molitor Tenecin 1	[Swiss-Prot: Q27023]
Triatoma brasiliensis Defensin 1	[Swiss-Prot: Q4VSI0]
Zophobas atratus Peptide B	[GenBank: AAB20745]
Zophobas atratus Peptide C	[GenBank: AAB20746]
**Arachnida**	
Androctonus australis hector Toxin II	[PDB: 1AHO]
Androctonus mauritanicus mauritanicus Po-Nh2	[PDB: 1PNH]
Androctonus mauritanicus mauritanicus Kaliotoxin	[PDB: 2KTX]
Androctonus mauritanicus mauritanicus P01	[PDB: 1ACW]
Argiope sp. Defensin	[GenBank: AAW01790]
Buthus occitanus tunetanus LVP1a	[Swiss-Prot: P84810]
Buthus occitanus tunetanus LVP1b	[Swiss-Prot: P84809]
Centruroides exilicauda Scorpion toxin variant 3	[PDB: 2SN3]
Centruroides exilicauda Neurotoxin	[PDB: 1NH5]
Centruiroides limbatus Hongotoxin	[PDB: 1HLY]
Centruroides limpidus Cll-dlp	[Swiss-Prot: Q6GU94]
Centruroides margaritatus Margatoxin	[PDB: 1MTX]
Centruroides noxius Toxin 2	[PDB: 1CN2]
Centruroides noxius Toxin Cn12	[PDB: 1PE4]
Centruroides noxius Cobatoxin	[PDB: 1PJV]
Centruroides noxius Noxiustoxin	[PDB: 1SXM]
Centruroides noxius Ergtoxin	[PDB: 1PX9]
Centruroides sculpturatus CsEv2 neurotoxin	[Swiss-Prot: P01493]
Centruroides sculpturatus Variant 1 neurotoxin	[PDB: 1VNA]
Centruroides sculpturatus Beta-neurotoxin	[PDB: 2B3C]
Centruroides sculpturatus Neurotoxin	[PDB: 1NRA]
Cupiennius salei Defensin	[5]
Dermacentor andersoni Defensin	[GenBank: ABK62866]
Dermacentor marginatus Defensin	[GenBank: ACJ04433]
Dermacentor variabilis Varisin A1	[Swiss-Prot: Q86QI5]
Haemaphysalis longicornis Longicin	[Swiss-Prot: Q58A47]
Heterometrus spinifer Hstx1	[PDB: 1QUZ]
Hottentotta judaicus (Buthotus judaicus) Toxin Bjxtr-It	[PDB: 1BCG]
Ixodes ricinus Defensin 1	[GenBank: AAP94724]
Ixodes ricinus Defensin 2	[GenBank: ABC88432]
Ixodes ricinus Defensin MT2	[GenBank: JAA65352]
Ixodes ricinus Defensin MT3	[GenBank: JAA71488]
Ixodes ricinus Defensin MT4	[GenBank: JAA71477]
Ixodes ricinus Defensin MT5	[GenBank: JAA66832]
Ixodes ricinus Defensin MT6	[GenBank: JAA71516]
Ixodes ricinus Defensin MT7	[GenBank: JAA69779]
Ixodes scapularis Scapularisin 1	[GenBank: EEC08934]
Ixodes scapularis Scapularisin 2	[GenBank: ABJB010277423]
Ixodes scapularis Scapularisin 3	[GenBank: EEC13914]
Ixodes scapularis Scapularisin 4	[GenBank: ABJB010377501]
Ixodes scapularis Scapularisin 5	[GenBank: EEC08933]

Ixodes scapularis Scapularisin 6	[GenBank: AAV74387]
Ixodes scapularis Scapularisin 7	[GenBank: ABJB011101692]
Ixodes scapularis Scapularisin 8	[GenBank: ABJB011096562]
Ixodes scapularis Scapularisin 9	[GenBank: ABJB011065840]
Ixodes scapularis Scapularisin 10	[GenBank: ABJB010379933]
Ixodes scapularis Scapularisin 11	[GenBank: ABJB011080676]
Ixodes scapularis Scapularisin 15	[GenBank: ABJB010719289]
Ixodes scapularis Scapularisin 16	[GenBank: EEC17916]
Ixodes scapularis Scapularisin 19	[GenBank: EEC01374]
Ixodes scapularis Scapularisin 20	[GenBank: EEC17844]
Ixodes scapularis Scapularisin 22	[GenBank: EEC03289]
Ixodes scapularis Scapularisin 23	[GenBank: ABJB010061346]
Ixodes scapularis Scapularisin 24	[GenBank: ABJB010520452]
Ixodes scapularis Scapularisin 25	[GenBank: ABJB010066055]
Ixodes scapularis Scasin 1	[GenBank: EEC18782]
Ixodes scapularis Scasin 2	[GenBank: ABJB010977893]
Ixodes scapularis Scasin 4	[GenBank: ABJB010586641]
Ixodes scapularis Scasin 5	[GenBank: ABJB010739942]
Ixodes scapularis Scasin 7	[GenBank: ABJB010767832]
Ixodes scapularis Scasin 8	[GenBank: ABJB010495451]
Ixodes scapularis Scasin 9	[GenBank: ABJB010461586]
Ixodes scapularis Scasin 10	[GenBank: ABJB010760265]
Ixodes scapularis Scasin 11	[GenBank: ABJB010086782]
Ixodes scapularis Scasin 13	[GenBank: ABJB010272194]
Ixodes scapularis Scasin 14	[GenBank: ABJB010907808]
Ixodes scapularis Scasin 16	[GenBank: ABJB011065775]
Ixodes scapularis Scasin 17	[GenBank: ABJB010453558]
Ixodes scapularis Scasin 18	[GenBank: ABJB010836816]
Ixodes scapularis Scasin 19	[GenBank: ABJB011050833]
Ixodes scapularis Scasin 21	[GenBank: ABJB010067897]
Leiurus quinquestriatus Chlorotoxin	[PDB: 1CHL]
Leiurus quinquestriatus hebraeus Defensin	[Swiss-Prot: P41965]
Leiurus quinquestriatus hebraeus Charybdotoxin	[Swiss-Prot: P13487]
Leiurus quinquestriatus hebraeus Insecticidal alpha scorpion toxin	[PDB: 1LQI]
Leiurus quinquestriatus hebraeus Alpha-like toxin Lgh Iii	[PDB: 1BMR]
Leiurus quinquestriatus hebraeus Scyllatoxin	[PDB: 1SCY]
Leiurus quinquestriatus hebraeus Lq2	[PDB: 1LIR]
Leiurus quinquestriatus hebraeus Agitoxin	[PDB: 1AGT]
*Leiurus quinquestriatus quinquestriatus*Anti-mammal and anti-insect LQQii toxin	[PDB: ILQQ]
Mesobuthus eupeus Herg-specific scorpion toxin Bekm-1	[PDB: 1LGL]
Mesobuthus eupeus Insectotoxin	[PDB: 1SIS]
*Mesobuthus martensii (Buthus)*K-channel toxin	[Swiss-Prot: Q9NII6]
Mesobuthus martensii (Buthus) Neurotoxin Bmk M1	[PDB: 1SN1]
Mesobuthus martensii (Buthus) Neurotoxin Bmk M4	[PDB: 1SN4]

Mesobuthus martensii (Buthus) Alpha-like toxin Bmk M7	[PDB: 1KV0]
Mesobuthus martensii (Buthus) Neurotoxin Bmk M8	[PDB: 1SNB]
Mesobuthus martensii (Buthus) Alpha-insect toxin Bmkait 1	[PDB: 1OMY]
Mesobuthus martensii (Buthus) Neurotoxin	[PDB: 1CHZ]
Mesobuthus martensii (Buthus) Bmbktx1	[PDB: 1Q2K]
Mesobuthus martensii (Buthus) BmTx2	[PDB: 2BMT]
Mesobuthus martensii (Buthus) Bmktx	[PDB: 1BKT]
Mesobuthus martensii (Buthus) BmTx3	[PDB: 1M2S]
Mesobuthus martensii (Buthus) Bmp01	[PDB: 1WM7]
Mesobuthus martensii (Buthus) Bmp02	[PDB: 1DU9]
Mesobuthus martensii (Buthus) Bmp03	[PDB: 1WM8]
Mesobuthus martensii (Buthus) Bmp07	[PDB: 1PVZ]
Mesobuthus martensii (Buthus) Mesotoxin	[Swiss-Prot: A0F0C2]
Mesobuthus tamulus Neurotoxin	[PDB: 1DQ7]
Ornithoctonus hainana Defensin	[6]
Ornithodoros moubata Defensin A	[Swiss-Prot: Q9BLJ3]
Ornithodoros moubata Defensin B	[GenBank: BAB41027]
Ornithodoros moubata Defensin C	[GenBank: BAC22074]
Ornithodoros moubata Defensin D	[GenBank: BAC22073]
Ornithodoros papillipes Defensin A	[GenBank: ACJ04425]
Ornithodoros papillipes Defensin B	[GenBank: ACJ04426]
Ornithodoros papillipes Defensin D	[GenBank: ACJ04427]
Ornithodoros puertoricensis Defensin A	[7]
Ornithodoros puertoricensis Defensin B	[7]
Ornithodoros rostratus Defensin A	[GenBank: ACJ04428]
Ornithodoros tartakovskyi Defensin A	[GenBank: ACJ04431]
Ornithodoros tartakovskyi Defensin B	[GenBank: ACJ04432]
Orthochirus scrobiculosus Osk1 toxin	[PDB: 1SCO]
Pandinus imperator Scorpine	[Swiss-Prot: P56972]
Pandinus imperator Toxin K-beta	[PDB: 1C49]
Pandinus imperator Toxin K-A (Pitx-Ka)	[PDB: 2PTA]
Pandinus imperator Pi7 non-toxic peptide	[PDB: 1QKY]
Pandinus imperator Pi4	[PDB: 1N8M]
Phoneutria reidyi Defensin	[5]
Polybetes pythagoricus Defensin	[5]
Scorpio maurus Maurotoxin	[PDB: 1TXM]
Tegenaria atrica Defensin	[5]
Tityus serrulatus Neurotoxin Ts1	[PDB: 1B7D]
Tityus serrulatus Tityustoxin	[PDB: 1HP2]
Tityus serrulatus Ts kappa	[PDB: 1TSK]
Tityus serrulatus Butantoxin	[PDB: 1C56]
**Crustacea**	
Daphnia pulex	[GenBank: EFX83704]
Daphnia pulex	[GenBank: EFX73794]
Daphnia pulex	[GenBank: FE421610]
Daphnia pulex	[GenBank: FE339229]

Daphnia pulex	[GenBank: FE339755]
Litopenaeus vannamei	[GenBank: FE088090]
**Myriapoda**	
Archispirostreptus gigas	[GenBank: FN197329]
Scolopendra canidens Defensin	[GenBank: AAW01788]
**Tardigrada**	
Hypsibius dujardini Defensin 1	[TardiBASE: **HDC02701**; GenBank: CK326474, CK326631, CK326718, CK326749, CO741228]
Hypsibius dujardini Defensin 2	[GenBank: **CO741284**, CD449294, CD449920, CO508161]
Hypsibius dujardini Defensin 3	[GenBank: **CO741737**, CO742078, CK326534]
Hypsibius dujardini Defensin 4	[GenBank: **CD449538,** CO501858]
Hypsibius dujardini Defensin 5	[GenBank: CO507946]
Hypsibius dujardini Defensin 6	[GenBank: CO508499]
Hypsibius dujardini Defensin 7	[GenBank: CO741157]
Hypsibius dujardini Defensin 8	[GenBank: CO741522]
Milnesium tardigradum Defensin 1	[GenBank: **GR863249,** GR861903]
Milnesium tardigradum Defensin 2	[GenBank: GR861547]
Milnesium tardigradum Defensin 3	[GenBank: GR860666]
Milnesium tardigradum Defensin 4	[GenBank: GR862104]
Milnesium tardigradum Defensin 5	[GenBank: GR861858]
Milnesium tardigradum Defensin 6	[GenBank: GR861612]
Milnesium tardigradum Defensin 7	[GenBank: GR865187]
Milnesium tardigradum Defensin 8	[GenBank: GR864191]
**Onychophora**	
Peripatopsis sedgwicki	[GenBank: FN237260]
Peripatopsis sedgwicki	[GenBank: FN236522]
Peripatopsis sedgwicki	[GenBank: FN243200]
Peripatopsis sedgwicki	[GenBank: FN241528]
Peripatopsis sedgwicki	[GenBank: FN239939]
Peripatopsis sedgwicki	[GenBank: **FN233877**, FN242990, FN242919]
Peripatopsis sedgwicki	[GenBank: **FN239399**, FN243038, FN242834]
Peripatopsis sedgwicki	[GenBank: FN243111]
Peripatopsis sedgwicki	[GenBank: FN242191]
**Nematoda**	
Ancylostoma caninum	[NEMBASE: ACC38241]
Ancylostoma caninum	[NEMBASE: ACC17687]
Ancylostoma ceylanicum	[NEMBASE: AYC00474]
Ancylostoma ceylanicum	[NEMBASE: AYC00542]
Ascaris lumbricoides	[NEMBASE: ALC00086]
Ascaris lumbricoides	[NEMBASE: ALC00177]
Ascaris lumbricoides	[NEMBASE: ALC00180]
Ascaris suum ASABF-alpha	[GenBank: BAA89497]

Ascaris suum ASABF-6Cys-alpha	[GenBank: BAC41496]
Ascaris suum ASABF-beta	[GenBank: BAC00497]
Ascaris suum ASABF-gamma	[GenBank: BAC00498]
Ascaris suum ASABF-delta	[GenBank: BAC00499]
Ascaris suum ASABF-epsilon	[GenBank: BAC41495]
Ascaris suum ASABF-zeta	[GenBank: BAC57992]
Ascaris suum	[NEMBASE: ASC03701]
Ascaris suum	[NEMBASE: ASC03066]
Ascaris suum	[NEMBASE: ASC17142]
Bursaphelenchus xylophilus	[NEMBASE: BXC00487]
Caenorhabditis brenneri	[NEMBASE: CBC04439]
Caenorhabditis brenneri	[WormBase: CBN15906]
Caenorhabditis brenneri	[WormBase: CBN16351]
Caenorhabditis brenneri	[WormBase: CBN14150]
Caenorhabditis brenneri CbnABF6	[WormBase: CBN15115]
Caenorhabditis brenneri	[WormBase: CBN22403]
Caenorhabditis brenneri	[WormBase: CBN15401]
Caenorhabditis brenneri	[WormBase: CBN00173]
Canorhabditis briggsae CbrABF2	[WormBase: CBG11981]
Canorhabditis briggsae	[WormBase: CBG09224]
Canorhabditis briggsae CbrABF5	[WormBase: CBG00070]
Canorhabditis briggsae CbrABF6	[WormBase: CBG00069]
Canorhabditis briggsae	[WormBase: CBG09786]
Canorhabditis briggsae	[WormBase: CBG21681]
Caenorhabditis elegans CeABF1	[NCBI Reference Sequence: NP_491253]
Caenorhabditis elegans CeABF2	[NCBI Reference Sequence: NP_491252]
Caenorhabditis elegans CeABF3	[NCBI Reference Sequence: NP_506950]
Caenorhabditis elegans CeABF4	[NCBI Reference Sequence: NP_507965]
Caenorhabditis elegans CeABF5	[NCBI Reference Sequence: NP_510136]
Caenorhabditis elegans CeABF6	[NCBI Reference Sequence: NP_741914]
Caenorhabditis elegans	[WormBase: Y38H6C.23]
Caenorhabditis elegans fipr-29	[WormBase: H06I04.7]
Caenorhabditis japonica	[WormBase: CJA09228]
Caenorhabditis japonica CjpABF6	[WormBase: CJA03860]
Caenorhabditis japonica	[NEMBASE: CJC05393]
Caenorhabditis japonica	[NEMBASE: CJC08906]
Caenorhabditis remanei	[WormBase: CRE07955]
Caenorhabditis remanei CreABF5	[WormBase: CRE24112]
Caenorhabditis remanei CreABF6	[WormBase: CRE24111]
Caenorhabditis remanei	[WormBase: CRE25171]
Caenorhabditis remanei	[WormBase: CRE02801]
Caenorhabditis remanei	[NEMBASE: CRC00359]

Caenorhabditis remanei Cremycin 1	[GenBank: AEM44803]
Caenorhabditis remanei Cremycin 2	[GenBank: AEM44804]
Caenorhabditis remanei Cremycin 3	[GenBank: AEM44805]
Caenorhabditis remanei Cremycin 4	[8]
Caenorhabditis remanei Cremycin 5	[GenBank: AEM44806]
Caenorhabditis remanei Cremycin 6—paper sequence doesn't match	[GenBank: AEM44807]
Caenorhabditis remanei Cremycin 7	[GenBank: AEM44808]
Caenorhabditis remanei Cremycin 8	[8]
Caenorhabditis remanei Cremycin 9	[GenBank: AEM44809]
Caenorhabditis remanei Cremycin 10	[WormBase: CRE14278]
Caenorhabditis remanei Cremycin 11	[GenBank: AEM44810]
Caenorhabditis remanei Cremycin 12	[WormBase: CRE14279]
Caenorhabditis remanei Cremycin 13	[GenBank: AEM44811]
Caenorhabditis remanei Cremycin 14	[8]
Caenorhabditis remanei Cremycin 15—mislabeled as Cremycin 14	[GenBank: AEM44812]
Caenorhabditis remanei	[WormBase: CRE14287]
Caenorhabditis sp. 5 AC-2008	[NEMBASE: CSC00683]
Caenorhabditis sp. 5 AC-2008	[NEMBASE: CSC01351]
Haemonchus contortus	[NEMBASE:HCC06231]
Haemonchus contortus	[NEMBASE: HCC02570]
Haemonchus contortus	[NEMBASE: HCC02002]
Meloidogyne hapla Mehamycin	[NEMBASE: MHC07481]
Meloidogyne hapla	[NEMBASE: MHC08122]
Meloidogyne hapla	[NEMBASE: MHC08639]
Meloidogyne hapla	[NEMBASE: MHC07477]
Meloidogyne hapla	[NEMBASE: MHC08815]
Meloidogyne javanica	[NEMBASE: MJC04218]
Necator americanus	[NEMBASE: NAC00042]
Necator americanus	[GenBank: GE625123]
Nippostrongylus brasiliensis	[NEMBASE: NBC02237]
Panagrolaimus superbus	[NEMBASE: PSC02929]
Parastrongyloides trichosuri	[NEMBASE: PTC03950]
Parastrongyloides trichosuri	[NEMBASE: PTC03906]
Pratylenchus penetrans	[NEMBASE: PEC00189]
Pristionchus pacificus	[WormBase: PPA22162]
Pristionchus pacificus	[WormBase: PPA01653]
Pristionchus pacificus	[NEMBASE: PPC03474]
Rotylenchulus reniformis	[GenBank: GT737647]
Teladorsagia circumcincta	[NEMBASE: TDC00905]
Toxascaris leonina	[NEMBASE: TLC00308]
Toxascaris leonina	[NEMBASE: TLC00068]
Toxocara canis	[NEMBASE: TCC00437]
Toxocara canis	[NEMBASE: TCC02268]
Toxocara canis	[NEMBASE: TCC00565contig2]

Toxocara canis	[NEMBASE: TCC02370]
Toxocara canis	[NEMBASE: TCC00389]
Xiphinema index	[NEMBASE: XIC00639]
Xiphinema index	[NEMBASE: XIC04611]
**Bivalvia**	
Crassostrea gigas Defensin	[GenBank: AJ565499]
Crassostrea virginica Defensin	[Swiss-Prot: P85008]
Cristaria plicata Defensin	[GenBank: AGG86914]
Dreissena polymorpha Defensin (Dpd)	[GenBank: ACZ02692]
Hyriopsis cumingii HcDef1	[9]
Hyriopsis cumingii HcDef2	[9]
Hyriopsis cumingii HcDef3	[GenBank: AEX88475]
Hyriopsis cumingii HcDef4	[9]
Hyriopsis cumingii HcDef5	[9]
Hyriopsis cumingii HcDef6	[9]
Hyriopsis cumingii Hc theromacin	[GenBank: GU123628]
Mytilus edulis Defensin A	[Swiss-Prot: P81610]
Mytilus edulis Defensin B	[Swiss-Prot: P81611]
Mytilus edulis Mytilin A	[Swiss-Prot: P81612]
Mytilus edulis Mytilin B	[Swiss-Prot: P81613]
Mytilus galloprovincialis MGD-1	[Swiss-Prot: P80571]
Mytilus galloprovincialis MGD2	[Swiss-Prot: Q9U6U0]
Mytilus galloprovincialis Myticin A	[Swiss-Prot: P82103]
Mytilus galloprovincialis Myticin B	[Swiss-Prot: P82102]
Mytilus galloprovincialis Mytilin B	[GenBank: AAD45013]
Mytilus galloprovincialis Mytilin C	[10]
Mytilus galloprovincialis Mytilin D	[GenBank: ACF21701]
Mytilus galloprovincialis Mytilin G1	[10]
Mytilus galloprovincialis Mytimacin 1	[GenBank: CCC15015]
Mytilus galloprovincialis Mytimacin 2	[GenBank: CCC15016]
Mytilus galloprovincialis Mytimacin 3	[GenBank: CCC15017]
Mytilus galloprovincialis Mytimacin 4	[GenBank: CCC15018]
Mytilus galloprovincialis Mytimacin 5	[GenBank: CCC15019]
Ruditapes philippinarum Defensin	[GenBank: AEK78067]
**Gastropoda**	
Achatina fulica Mytimacin-AF	[GenBank: AFR36920]
Aplysia californica	[NCBI Reference Sequence: XP_005103372]
Aplysia californica	[NCBI Reference Sequence: NP_001191629]
Aplysia californica	[NCBI Reference Sequence: XP_005095540]
Haliotis discus discus Defensin	[Swiss-Prot: D3UAH2]
Littorina saxatalis	[GenBank: FR864485]
**Echinodermata**	
Asterina pectinifera/Patiria pectinifera	[GenBank: DB418655]
Asterina pectinifera/Patiria pectinifera	[GenBank: DB424270]

Patiria miniata	[GenBank: EX453588]
**Annelida**	
Hirudo medicinalis Neuromacin	[Swiss-Prot: A8V0B3]
Hirudo medicinalis Theromacin	[Swiss-Prot: A8I0L8]
Theromyzon tessulatum Theromacin	[GenBank: AAR12065]
**Platyhelminthes**	
Schistosoma mansoni	[GenBank: **EX499237**, EX499238, EX499230]
Schistosoma mansoni	[GenBank: **EX499261**, EFX499213]
Schistosoma mansoni	[GenBank: **EX499243**, EX499221, EX499222, EX499244]
Schistosoma mansoni	[GenBank: EX499256]
**Plantae**	
Aesculus hippocastanum Ah-AMP1	[GenBank: AAB34970]
Arabidopsis halleri Ah-PDF1.1	[GenBank: AAY27736]
Arabidopsis thaliana PDF1.1	[NCBI Reference Sequence: NP_565119]
Arabidopsis thaliana PDF1.2	[Swiss-Prot: Q9FI23]
Arabidopsis thaliana PDF1.3	[NCBI Reference Sequence: NP_180171]
Arabidopsis thaliana Trypsin inhibitor	[PDB: 1JXC]
Arachis hypogaea Peanut allergen Arah12	[GenBank: EY396089]
Arachis hypogaea Peanut allergen Arah13	[GenBank: EY396019]
Beta vulgaris AX1	[Swiss-Prot: P81493]
Beta vulgaris AX2	[Swiss-Prot: P82010]
Brassica campestris BSD1	[11]
Brassica oleracea PCP-A1	[GenBank: CAA06464]
Capsicum annuum CaDEF1	[GenBank: AAL35366]
Capsicum annuum J1-1	[Swiss-Prot: Q43413]
Capsicum chinense Gamma-thionin	[GenBank: AAD21200]
Cassia fistula Peptide 5459	[12]
Cassia fistula Peptide 5144	[12]
Clitoria ternatea Ct-AMP1	[GenBank: AAB34971]
Dahlia merckii Dm-AMP1	[GenBank: AAB34972]
Echinocloa crusgalli Ec-AMP-D1	[Swiss-Prot: P86518]
Echinocloa crusgalli Ec-AMP-D2	[13]
Elaeis guineensis EGAD1	[GenBank: AF322914]
Ginkgo biloba Gbd	[GenBank: AAU04859]
Glycine max Defensin	[Swiss-Prot: Q07502]
Hardenbergia violacea HvAMP1	[14]
Helianthus annuus HaDEF1	[GenBank: AF364865]
Helianthus annuus SD2	[Swiss-Prot: P82659]
Heuchera sanguinea Hs-AMP1	[Swiss-Prot: P0C8Y5]
Hordeum vulgare Gamma-hordothionin	[Swiss-Prot: P20230]
Hordeum vulgare Omega-hordothionin	[15]
Ipomoea batatas SPD1	[GenBank: AY552546]
Lens culinaris LcDef	[GenBank: ABP04037]

Lepidium meyenii Lm-def	[GenBank: AAV85992]
Medicago sativa MsDEF1	[GenBank: AAG40321]
Mendicago trucatula MtDEF2	[GenBank: AAQ91290]
Nicotiana alata NaD1	[Swiss-Prot: Q8GTM0]
Nicotiana megalosiphon Nm-Def02, Nmdef2	[GenBank: ACR46857]
Nigella sativa Ns-D1	[Swiss-Prot: P86972]
Nigella sativa Ns-D2	[Swiss-Prot: P86973]
Pachyrrhizus erosus SPE10	[GenBank: AAT80338]
Pentadiplandra brazzeana Brazzein (sweet tasting protein)	[Swiss-Prot: P56552]
Petunia hybrida PhD1	[Swiss-Prot: Q8H6Q1]
Petunia hybrida PhD2	[Swiss-Prot: Q8H6Q0]
Phaseolus limensis BLBAFP	[16]
Phaseolus vulgaris PvD1	[GenBank: ADR30066]
Phaseolus vulgaris WCBAFP	[17]
Pinus sylvestris PsDef1	[Swiss-Prot: A4L7R7]
Pisum sativum PsD1, Psd1	[Swiss-Prot: P81929]
Pisum sativum PsD2, Psd2	[Swiss-Prot: P81930]
Prunus persica Pp-def1	[GenBank: AAL85480]
Raphanus sativus RsAFP1	[GenBank: AAA69541]
Raphanus sativus RsAFP2	[GenBank: AAA69540]
Saccharum officinarum Sd1	[GenBank: CA112870]
Saccharum officinarum Sd2	[GenBank: CA095771]
Saccharum officinarum Sd3	[GenBank: CA259771]
Saccharum officinarum Sd4	[GenBank: CA259589]
Saccharum officinarum Sd5	[GenBank: CA297803]
Saccharum officinarum Sd6	[GenBank: CA188998]
Solanum lycopersicum DEF2	[GenBank: AW623541]
Sorghum bicolor SIalpha1	[Swiss-Prot: P21923]
Sorghum bicolor SIalpha2	[Swiss-Prot: P21924]
Sorghum bicolor SIalpha3	[Swiss-Prot: P21925]
Spinacea oleracea SoD2	[Swiss-Prot: P81571]
Stellaria media Sm-AMP-D1	[18]
Tephrosia villosa TvD1	[GenBank: AAX86993]
Trichosanthes kirilowii TDEF	[GenBank: ABF74600]
Trigonella foenum- graecum Tfgd1	[GenBank: AAO72632]
Triticum aestivum TAD1	[GenBank: BAC10287]
Triticum kiharae Tk-AMP-D1	[Swiss-Prot: P84963]
Triticum monococcum Tm-AMP-D1.2	[Swiss-Prot: P84964]
Triticum turgidum Gamma1-purothionin	[PRF: 227202]
Triticum turgidum Gamma2-purothionin	[PRF: 227203]
Vicia faba Defensin	[GenBank: ACI02059]
Vigna angularis VaD1	[19]
Vigna radiata VrD1	[GenBank: AAR08912]
Vigna radiata VrCRP	[20]
Vigna unguiculata Cp-thionin I	[Swiss-Prot: P83399]
Vigna unguiculata Cp-thionin II	[21]

Vigna unguiculata VUDEF	[22]
Vitis vinifera Vv-AMP1	[23]
Wasabia japonica WT1	[GenBank: AB012871]
Zea mays Gamma-1-zeathionin	[Swiss-Prot: P81008]
Zea mays Gamma-2-zeathionin, PDC-1	[Swiss-Prot: P81009]
Zea mays ZmESR-6	[NCBI Reference Sequence: NP_001105777]
**Fungi**	
Ajellomyces capsulatus/Histoplasma capsulatum Acasin	[GenBank: AAJI01002159]
Ajellomyces capsulatus/Histoplasma capsulatum Acapsin 1, 2	[24]
Ajellomyces dermatitidis/Blastomyces dermatitidis Adersin 1, 2	[24]
Arthroderma otae/Microsporum canis Micasin	[GenBank: JN014007]
Arthroderma otae/Microsporum canis Micasin 1	[GenBank: JN014008]
Arthroderma benhamiae Arbesin	[24]
Arthroderma gypseum Agysin	[24]
*Aspergillus clavatus*Aclasin	[24]
Aspergillus clavatus Aclasin, N-term	[GenBank: AAKD03000014]
Aspergillus clavatus Aclasin, C-term	[GenBank: AAKD03000014]
Aspergillus flavus Aflasin 1, N-term	[25]
Aspergillus flavus Aflasin 1, C-term	[25]
Aspergillus fumigatus (flavus?) Aflasin 2	[24, 25]
Aspergillus flavus Aflasin 3	[25]
Aspergillus fumigatus Afusin, N-term	[NCBI Reference Sequence: XP_748991]
Aspergillus fumigatus Afusin, C-term	[NCBI Reference Sequence: XP_748991]
Aspergillus nidulans Anisin 1	[NCBI Reference Sequence: XP_662650]
Aspergillus nidulans Anisin 2	[GenBank: AACD01000108]
Aspergillus oryzae Aorsin, N-term	[GenBank: BAE56652]
Aspergillus oryzae Aorsin, C-term	[GenBank: BAE56652]
Aspergillus terreus Atesin 1	[GenBank: AAJN01000157]
Aspergillus terreus Atesin 2	[NCBI Reference Sequence: XP_001209108]
Aspergillus terreus Atesin 3	[GenBank: EF541165]
Chaetomium globosum Cglosin 1, N-term	[GenBank: EAQ86375]
Chaetomium globosum Cglosin 1, C-term	[GenBank: EAQ86375]
Chaetomium globosum Cglosin2	[GenBank: AAFU01000488]
Laccaria bicolor Labisin	[24]
Neosartorya fischeri Nefisin	[24]
Neosartorya fischeri Nefisin 2, N-term	[GenBank: AAKE03000016]
Neosartorya fischeri Nefisin 2, C-term	[GenBank: AAKE03000016]
Neosartorya fischeri Nefisin 1, N-term	[25]
Neosartorya fischeri Nefisin 1, C-term	[25]
Penicillium chrysogenum Pechrysin	[24]
Pseudoplectania nigrella Plectasin	[Swiss-Prot: Q53I06]

Rhizopus oryzae Rorsin 1	[GenBank: AACW02000043]
Rhizopus oryzae Rorsin 2	[GenBank: AACW02000259]
Trichophyton rubrum Trirusin	[24]
Trichophyton tonsurans Tritosin	[24]
Trichophyton verrucosum Trivesin	[24]
Verticillium albo-atrum Vasin	[24]
Verticillium dahliae Vedasin	[24]
**Human**	
Homo sapiens Drosomycin-like defensin (DLD)	[GenBank: AK024601]

Note: Scapularisins 12, 13, 17,18, 21 and Scasins 3, 6, 12, 15, 20 from [26] were not included because the Accession numbers could not be found in the database.

References

1. Lauth X, Nesin A, Briand J-P, Roussel J-P, Hetru C. Isolation, characterization and chemical synthesis of a new insect defensin from *Chironomus plumosus* (Diptera). Insect Biochem Molec Biol. 1998 December;28(12):1059-66.
2. Taguchi S, Bulet P, Hoffmann JA. A novel insect defnsin from the ant *Formica rufa*. Biochimie. 1998 April;80(4):343-6.
3. Pöppel A-K, Vogel H, Wiesner J, Vilcinskas A. Antimicrobial Peptides Expressed in Medicinal Maggots of the Blow Fly *Lucilia sericata* Show Combinatorial Activity against Bacteria. Antimicrobial Agents and Chemotherapy. 2015;59(5):2508-14.
4. Gao B, Zhu S. Identification and characterization of the parasitic wasp *Nasonia* defensins: Positive selection targeting the functional region? Developmental and Comparative Immunology. 2010 June;34(6):659-68.
5. Baumann T, Kuhn-Nentwig L, Largiadèr CR, Nentwig W. Expression of defensins in non-infected araneomorph spiders. Cell Mol Life Sci. 2010 Aug;67(15):2643-51.
6. Zhou H, Kong Y, Wang H, Tianhua Y, Feng F, Bian J, et al. A defensin-like antimicrobial peptide from the venoms of spider, *Ornithoctonus hainana*. Journal of Peptide Science. 2011 April 2011;17:540-4.
7. Chrudimská T, Chrudimský T, Golovchenko M, Rudenko N, Grubhoffer L. New defensins from hard and soft ticks: Similarities, differences, and phylogenetic analyses. Veterinary Parasitology. 2010;167:298-303.
8. Zhu S, Gao B. Nematode-derived drosomycin-type antifungal peptdies provide evidence for plant-to-ecdysozoan horizontal transfer of a disease resistance gene. Nature Communications. 2014;5.
9. Ren Q, Li M, Zhang C-Y, Chen K-P. Six defensins from the triangle-shell pearl mussel *Hyriopsis cumingii*. Fish and Shellfish Immunology. 2011;31:1232-8.
10. Mitta G, Vandenbulcke F, Hubert F, Salzet M, Roch P. Involvement of Mytilins in Mussel Antimicrobial Defense. Journal of Biological Chemistry. 2000 April 28;275(17):12954-62.
11. Park HC, Kang YH, Chun HJ, Koo JC, Cheong YH, Kim CY, et al. Characterization of a stamen-specific cDNA encoding a novel plant defensin in Chinese cabbage. Plant Molecular Biology. 2002 Sept.;50(1):59-69.
12. Wijaya R, Neumann GM, Condron R, Hughes AB, Polya GM. Defense proteins from seed of Cassia fistula include a lipid transfer protein homologue and a protease inhibitory defensin. Plant Science. 2000;159:243-55.
13. Odintsova TI, Rogozhin EA, Baranov Y, Musolyamov AK, Yalpani N, Egorov TA, et al. Seed defensins of barnyard grass *Echinochloa crusgalli* (L.) Beauv. Biochimie. 2008 Nov-Dec;90(11-12):1667-73.

14. Harrison SJ, Marcus JP, Goulter KC, Green JL, Maclean DJ, Manners JM. An Antimicrobial Peptide from the Australian Native *Hardenbergia violacea* Provides the First Functionally Characterised Member of a Subfamily of Plant Defensins. Australian Journal of Plant Physiology. 1997;24(5):571-8.
15. Méndez E, Rocher A, Calero M, Girbés T, Citores L, Soriano F. Primary structure of ω-hordothionin, a member of a novel family of thionins from barley endosperm, and its inhibition of protein synthesis in eukaryotic and prokaryotic cell-free systems. Eur J Biochem. 1996;239:67-73.
16. Wang HX, Ng TB. An antifungal peptide from baby lima bean. Applied Microbiology and Biotechnology. 2006 Dec.;73(3):576-81.
17. Wong JH, Zhang XQ, Wang HX, Ng TB. A mitogenic defensin from white cloud beans (*Phaseolus vulgaris*). Peptides. 2006 Sept.;27(9):2075-81.
18. Slavokhotova AA, Odintsova TI, Rogozhin EA, Musolyamov AK, Andreev YA, Grishin EV, et al. Isolation, molecular cloning and antimicrobial activity of novel defensins from common chickweed (*Stellaria media* L.) seeds. Biochimie. 2011 March;93(3):450-6.
19. Chen G-H, Hsu M-P, Tan C-H, Sung H-Y, Kuo CG, Fan M-J, et al. Cloning and Characterization of a Plant Defensin VaD1 from Azuki Bean. Journal of Agricultural and Food Chemistry. 2005 Feb. 23;53(4):982-8.
20. Chen K-C, Lin C-Y, Kuan C-C, Sung H-Y, Chen C-S. A Novel Defensin Encoded by a Mungbean cDNA Exhibits Insecticida Activity against Bruchid. Journal of Agricultural and Food Chemistry. 2002 Dec. 4;50(25):7258-63.
21. Franco OL, Murad AM, Leite JR, Mendes PAM, Prates MV, Bloch C, Jr. Identification of a cowpea g-thionin with bactericidal activity. FEBS Journal. 2006 August;273(15):3489-97.
22. Carvalho AO, Machado OLT, Da Cunha M, Santos IS, Gomes VM. Antimicrobial peptides and immunolocalization of a LTP in *Vigna unguiculata* seeds. Plant Physiology and Biochemistry. 2001 Feb.;39(2):137-46.
23. de Beer A, Vivier MA. Vv-AMPI, a ripening induced peptide from *Vitis vinifera* shows strong antifungal activity. BMC Plant Biology. 2008 July 8;8:75.
24. Zhu S, Gao B, Harvey PJ, Craik DJ. Dermatophytic defensin with antiinfective potential. PNAS. 2012;109(22):8495-500.
25. Zhu S. Discovery of six families of fungal defensin-like peptides provides insights into origin and evolution of the CSab defensins. Mol Immunol. 2008;45:828-38.
26. Wang Y, Zhu S. The defensin gene family expansion in the tick *Ixodes scapularis*. Developmental and Comparative Immunology. 2011;35:1128-34.
